# Supplementary material for: Operational complexities in international clinical trials: a systematic review of challenges and proposed solutions
Source: BMJ Open. 2024 Apr 15;14(4):e077132. doi: 10.1136/bmjopen-2023-077132 (PMC11029458; doi:10.1136/bmjopen-2023-077132)
Supplement: Supplementary data [file bmjopen-2023-077132supp001.pdf]

Supplementary Material

1 LITERATURE SEARCH STRATEGY

A summary of the search and selection strategy is first depicted for quick reference (a); examples of the kinds of search terms that were used to identify each of four concepts, as described in the main manuscript are then provided in (b). Full and detailed database search strategies used in Medline, Embase and HMIC are then also provided (c) for reference.

a) Search and selection strategy

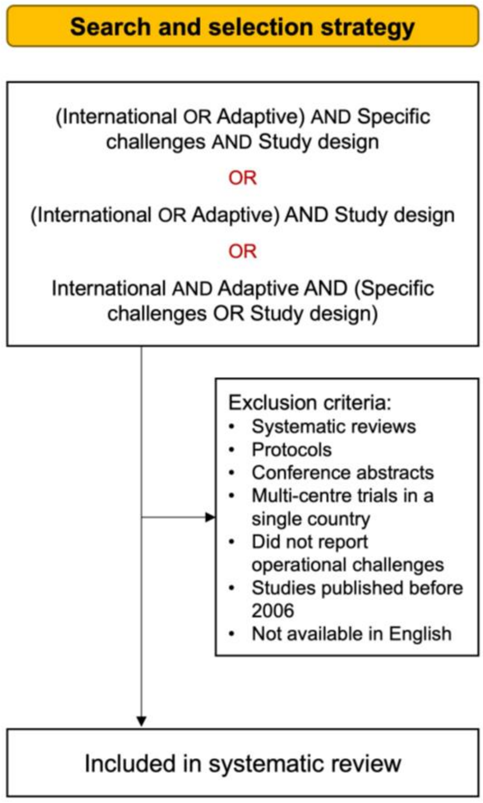

b) Examples of search terms used for each concept

International
International, multinational, intercontinental, multicentre, multisite, pan-European.

Adaptive
Adaptive, platform, umbrella, basket, bucket, master protocol.

Specific challenges
legal, operational, workforce, data management, insurance, indemnity, add or remove arms, gdpr, infrastructure, recruitment, sponsor, contract research, law, regulation, budget, finance, resource allocation, resource management, policy, Brexit, intervention, investigational, supply, logistics, distribution, delivery, monitoring, safety, capacity building, contract, funding, delegation, agreement, ethics, insurance, data sharing.

Study design
lessons, pitfalls, study design, methodology, challenge, operational, practical, guidelines, recommendations, rationale, implement, workflow, framework.

c) Detailed database search strategy.

Database: MEDLINE (Ovid), 31 January 2023

1	adaptive clinical trial/	37
2	((adaptive or umbrella or basket or bucket) adj2 (design or trial or trials or study or studies or protocol$)).mp.	4428
3	(trial$ platform$ or platform design or platform trial$ or platform clinical trial$ or platform study or platform studies or platform protocol$).mp.	856
4	((adaptive adj6 design$) and (trial or trials or study or studies or protocol$)).mp.	3077
5	(complex innovative adj3 (trial$ or design$ or protocol$)).mp.	17
6	master protocol$.mp.	242
7	response adaptive randomi$.mp.	139
8	(single centre or single site).mp.	33986
9	(or/1-7) not 8 [adaptive design - not single-site]	6597
10	(international adj5 (trial or trials or protocol$ or multicent$ or multi-cent$ or multisite or multi-site or multi-arm or multiarm or multi-stage or multistage)).mp.	19111
11	(international clinical$ trial$ regist$ or international clinical$ trial$ platform$ or international standard randomi$ control$ trial$ number$).mp.	3897
12	international.mp. /freq=2	93924
13	10 not (11 not 12) [gets rid of results with 'international trial' that only refer to the WHO ICTRP]	15678
14	((multinational or multi-national or intercontinental or inter-continental or pan-europ$) adj8 (trial or trials or protocol$ or multicent$ or multi-cent$ or multisite or multi-site)).mp.	3011
15	((trial or trials or protocol$) and (((multicent$ or multi-cent$ or multisite or multi-site) and sites) or centres or centers) and countries).mp.	5427
16	((UK or United Kingdom) and (EU or Europ$) and (trial$ or protocol$)).mp.	2982
17	(europe/ or european alpine region/ or andorra/ or austria/ or balkan peninsula/ or belgium/ or exp europe, eastern/ or exp france/ or exp germany/ or gibraltar/ or greece/ or ireland/ or exp italy/ or liechtenstein/ or luxembourg/ or exp mediterranean region/ or monaco/ or netherlands/ or portugal/ or san marino/ or exp "scandinavian and nordic countries"/ or spain/ or switzerland/ or exp transcaucasia/ or exp ussr/ or vatican city/) and exp United Kingdom/ and (trial$ or protocol$).mp.	1404
18	(exp africa/ or exp americas/ or exp asia/ or exp oceania/) and exp europe/ and (trial$ or protocol$).mp.	9851
19	or/13-18 [main international trial requirement]	35699
20	Methods/	231754
21	Research Design/	122769
22	challenge$.mp.	870696
23	(((trial$ or design) adj5 efficiency) or efficiencies).mp.	54394
24	((complexit$ or guidance or guidelines or recommendations or considerations or issues or obstacles or barriers) and trial$).ti.	5122
25	((lesson or lessons or pitfall or pitfalls) and (design$ or plan$)).mp.	27395
26	(trial$ adj5 (manag$ or run or running or conducting)).mp.	15671
27	(design$ or rationale$ or implement$ or methodol$).ti.	292767
28	(trial$ and (framework$ or regulations)).ti.	488
29	(trials and (Europ$ or EU or multinational or multi-national or intercontinental or inter-continental or international or multi-cent$ or multicent$ or multi-site or multisite)).ti.	2853
30	(trial$ adj1 (approval or authorisation or authorization or regulation)).mp.	341
31	"organization and administration"/ or capacity building/ or decision making, organizational/ or efficiency/ or efficiency, organizational/ or organizational culture/ or workforce/ or models, organizational/ or organizational innovation/ or change management/ or organizational objectives/ or personnel management/ or leadership/ or personnel selection/ or "personnel staffing and scheduling"/ or staff development/ or planning techniques/ or strategic planning/ or program development/ or total quality management/	306062
32	Multicenter Studies as Topic/	22031
33	exp *Clinical Trials as Topic/ or exp Clinical Trials as Topic/mt, st	68990
34	(trial$ and design$).kf.	2312

35	(((good or best) adj3 practice$) and trial$).ti.	204
36	(trial$ adj2 (authoris$ or authoriz$)).mp.	110
37	or/20-36 [general study design/methodology terms]	1888239
38	((trial$ adj5 insur$) or insuring or indemni$).mp.	2674
39	((add$ or remov$) adj5 (arm or arms)).mp.	3631
40	gdpr.mp.	365
41	exp *ethics/ or ethic$.ti.	121779
42	((data adj8 secur$) or (data adj3 (share$ or sharing)) or data management or data governance).mp. or computer security/ or data anonymization/	33659
43	data accuracy/	3708
44	(trial$ adj3 infrastructure).mp.	197
45	exp *models, statistical/ or (exp models, statistical/ and ((statistical adj3 (model$ or framework$ or plan$)) or estimand$).ti,ab.) or (statistical adj1 design$).mp.	59261
46	software/ or database management systems/ or software design/ or software validation/	133275
47	(reporting guideline$ or outcome reporting).kf. or reporting.ti. or (reporting adj4 (quality or guideline$)).mp.	37866
48	terminology/	0
49	(recruit$ adj4 (effective$ or model$ or pause$ or pausing)).mp. or recruit$.ti. or *Patient Selection/	50660
50	(decision$ adj2 algorithm$).mp.	2965
51	sponsor$.ti.	3863
52	fund$.ti.	43682
53	resourcing.mp.	1190
54	exp financial management/ or financing, organized/ or taxes/ or (financ$ not incentiv$).ti.	118701
55	economic$.ti.	57184
56	(trial$ adj1 cost$).mp. or (costs or costing).ti.	39270
57	contract research org$.mp.	428
58	(regulations or (regulat$ adj3 framework$)).mp.	60029
59	Government Regulation/	21840
60	jurisprudence/ or confidentiality/ or personally identifiable information/ or international law/ or legal services/ or liability, legal/ or mandatory reporting/ or legislation, drug/	80153
61	((legal$ or law or laws or legislat$ or policy or policies or rule or rules) adj5 (UK or United Kingdom or brit$ or EU or Europe$ or framework$ or national or government$)).mp.	50560
62	(clinical trial regulation or "536/2014").mp.	95
63	((EU or Europe or European) adj4 representative$).mp.	810
64	collaborat$.ti.	37346
65	(harmonis$ or harmoniz$).mp.	20656
66	brexit.mp.	771
67	(safety adj3 (monitor$ or governance)).mp. or Patient Safety/	31531
68	(((intervention or IMP or investigational medicin$ or drug$) adj6 (supply$ or inventory or export$ or import$)) or procur$).mp.	130269
69	(treatment selection or site selection).mp.	8819
70	workload$.mp.	51329
71	Contracts/ or (contract or contracts).mp.	37008
72	((fund$ or collaborat$ or delegat$ or site or sites or research or sponsor$) adj5 (agreement or agreements)).mp.	3553
73	((operational or practical or legal or administrative or financial or procedural or ethical or methodolog$ or statistical or recruitment or sponsor$ or logistic$ or design) adj4 (complexit$ or guidance or guidelines or recommendations or considerations or issues or challenges or obstacles or barriers or difficulties or advantages or disadvantages)).mp.	135867
74	exp *Clinical Trials as Topic/es, lj, og, st, sd [Ethics, Legislation & Jurisprudence, Organization & Administration, Supply & Distribution]	11020
75	or/38-74 [any of the various specific challenges]	1238686
76	(9 or 19) and 37 and 75	2818
77	(9 or 19) and (*Research Design/ or *Methods/)	1335
78	9 and 19 and (37 or 75)	72
79	76 or 77 or 78	3675
80	limit 79 to yr="2006 -Current"	3065
81	80 and (exp clinical trial/ or trial$.mp.)	2686

82	limit 81 to ("review" or "scientific integrity review" or "systematic review")	498
83	81 not (82 and (systematic and review).ti.)	2630
84	83 not (exp animals/ not humans.sh.)	2625
85	limit 84 to english language	2555

Database: EMBASE, 31 January 2023

1	adaptive clinical trial/	377
2	((adaptive or umbrella or basket or bucket) adj2 (design or trial or trials or study or studies or protocol$)).mp.	6949
3	(trial$ platform$ or platform design or platform trial$ or platform clinical trial$ or platform study or platform studies or platform protocol$).mp.	1567
4	((adaptive adj6 design$) and (trial or trials or study or studies or protocol$)).mp.	4842
5	(complex innovative adj3 (trial$ or design$ or protocol$)).mp.	29
6	master protocol$.mp.	439
7	response adaptive randomi$.mp.	207
8	(single centre or single site).mp.	66742
9	(or/1-7) not 8 [adaptive design - not single-site]	10465
10	(international adj5 (trial or trials or protocol$ or multicent$ or multi-cent$ or multisite or multi-site or multi-arm or multiarm or multi-stage or multistage)).mp.	32583
11	(international clinical$ trial$ regist$ or international clinical$ trial$ platform$ or international standard randomi$ control$ trial$ number$).mp.	4034
12	international.mp. /freq=2	165479
13	10 not (11 not 12) [gets rid of results with 'international trial' that only refer to the WHO ICTRP]	29033
14	((multinational or multi-national or intercontinental or inter-continental or pan-europ$) adj8 (trial or trials or protocol$ or multicent$ or multi-cent$ or multisite or multi-site)).mp.	5901
15	((trial or trials or protocol$) and (((multicent$ or multi-cent$ or multisite or multi-site) and sites) or centres or centers) and countries).mp.	9439
16	((UK or United Kingdom) and (EU or Europ$) and (trial$ or protocol$)).mp.	8673
17	(western europe/ or austria/ or exp belgium/ or benelux/ or exp france/ or germany/ or ireland/ or liechtenstein/ or luxembourg/ or monaco/ or netherlands/ or exp scandinavia/ or switzerland/ or exp Eastern Europe/ or exp Southern Europe/) and exp United Kingdom/ and (trial$ or protocol$).mp.	5785
18	((exp Eastern Europe/ and exp Southern Europe/) or (exp Eastern Europe/ and exp Western Europe/) or (exp Western Europe/ and exp Southern Europe/)) and (trial$ or protocol$).mp.	6768
19	(exp western hemisphere/ or exp africa/ or exp asia/ or exp "australia and new zealand"/) and exp Europe/ and (trial$ or protocol$).mp.	25734
20	or/13-19 [main international trial requirement]	77781
21	methodology/	1630438
22	study design/	54726
23	challenge$.mp.	1076548
24	(((trial$ or design) adj5 efficiency) or efficiencies).mp.	59423
25	((complexit$ or guidance or guidelines or recommendations or considerations or issues or obstacles or barriers) and trial$).ti.	6537
26	((lesson or lessons or pitfall or pitfalls) and (design$ or plan$)).mp.	35412
27	(trial$ adj5 (manag$ or run or running or conducting)).mp.	25255
28	(design$ or rationale$ or implement$ or methodol$).ti.	345887
29	(trial$ and (framework$ or regulations)).ti.	629
30	(trials and (Europ$ or EU or multinational or multi-national or intercontinental or inter-continental or international or multi-cent$ or multicent$ or multi-site or multisite)).ti.	4222
31	(trial$ adj1 (approval or authorisation or authorization or regulation)).mp.	579
32	management/ or joint venture/ or total quality management/ or work schedule/ or workflow/	183307
33	"multicenter study (topic)"/	38773
34	exp *"clinical trial (topic)"/	21048
35	(trial$ and design$).kf.	4092
36	(((good or best) adj3 practice$) and trial$).ti.	301

37	(trial$ adj2 (authoris$ or authoriz$)).mp.	223
38	or/21-37 [general study design/methodology terms]	3300880
39	((trial$ adj5 insur$) or insuring or indemni$).mp.	3566
40	((add$ or remov$) adj5 (arm or arms)).mp.	6264
41	gdpr.mp.	528
42	exp *ethics/ or ethic$.ti.	134708
43	((data adj8 secur$) or (data adj3 (share$ or sharing)) or data management or data governance).mp. or data protection/ or anonymization/ or data privacy/ or encryption/	37340
44	data quality/ or data accuracy/ or data availability/ or data completeness/ or data consistency/ or data validity/	8842
45	(trial$ adj3 infrastructure).mp.	316
46	*statistical model/ or *statistical analysis/ or ((statistical model/ or statistical analysis/) and ((statistical adj3 (model$ or framework$ or plan$)) or estimand$).ti,ab.) or statistical design/ or (statistical adj1 design$).mp.	55949
47	software/ or software design/ or general software/ or exp data analysis software/	203465
48	(reporting guideline$ or outcome reporting).kf. or reporting.ti. or (reporting adj4 (quality or guideline$)).mp.	48775
49	nomenclature/	60593
50	(recruit$ adj4 (effective$ or model$ or pause$ or pausing)).mp. or recruit$.ti. or *patient selection/	51069
51	(decision$ adj2 algorithm$).mp.	4194
52	sponsor$.ti.	4495
53	funding/ or fund$.ti.	114234
54	resourcing.mp.	1588
55	financial management/ or accounting/ or "billing and claims"/ or budget/ or finance/ or corporate finance/ or public finance/ or purchasing/ or exp tax/ or resource management/ or resource allocation/ or (financ$ not incentiv$).ti.	233201
56	economic evaluation/ or economic$.ti.	81922
57	(trial$ adj1 cost$).mp. or (costs or costing).ti.	52721
58	contract research org$.mp.	934
59	(regulations or (regulat$ adj3 framework$)).mp.	77414
60	*european medicines agency/ or *"medicines and healthcare products regulatory agency"/	604
61	legal aspect/ or government regulation/ or legal evidence/ or legal liability/ or legal procedure/ or legal service/ or medical liability/ or medicolegal aspect/ or jurisprudence/	281458
62	((legal$ or law or laws or legislat$ or policy or policies or rule or rules) adj5 (UK or United Kingdom or brit$ or EU or Europe$ or framework$ or national or government$)).mp.	68360
63	(clinical trial regulation or "536/2014").mp.	168
64	((EU or Europe or European) adj4 representative$).mp.	1071
65	collaborat$.ti.	45453
66	(harmonis$ or harmoniz$).mp.	31555
67	brexit.mp.	929
68	(safety adj3 (monitor$ or governance)).mp.	11693
69	(((intervention or IMP or investigational medicin$ or drug$) adj6 (supply$ or inventory or export$ or import$)) or procur$).mp.	114343
70	(treatment selection or site selection).mp.	12860
71	workload$.mp.	72251
72	contract/ or (contract or contracts).mp.	38623
73	((fund$ or collaborat$ or delegat$ or site or sites or research or sponsor$) adj5 (agreement or agreements)).mp.	5107
74	((operational or practical or legal or administrative or financial or procedural or ethical or methodolog$ or statistical or recruitment or sponsor$ or logistic$ or design) adj4 (complexit$ or guidance or guidelines or recommendations or considerations or issues or challenges or obstacles or barriers or difficulties or advantages or disadvantages)).mp.	168395
75	or/39-74 [any of the various specific challenges]	1787225
76	(9 or 20) and 38 and 75	4552
77	(9 or 20) and (*study design/ or *methodology/)	932
78	9 and 20 and (38 or 75)	192

79	76 or 77 or 78	5246
80	limit 79 to conference abstract	1834
81	79 not 80	3412
82	limit 81 to yr="2006 -Current"	3004
83	82 and (trial$.mp. or clinical research/ or exp clinical trial/)	2633
84	limit 83 to ("systematic review" or "review")	656
85	83 not (84 and (systematic and review).ti.)	2567
86	85 not ((exp animal/ or animal experiment/ or nonhuman/) not (exp human/ or human experiment/))	2557
87	limit 86 to english language	2478

Database: Health Management Information Consortium, 31 January 2023

1	((adaptive or umbrella or basket or bucket) adj2 (design or trial or trials or study or studies or protocol$)).mp.	15
2	(trial$ platform$ or platform design or platform trial$ or platform clinical trial$ or platform study or platform studies or platform protocol$).mp.	4
3	((adaptive adj6 design$) and (trial or trials or study or studies or protocol$)).mp.	8
4	(complex innovative adj3 (trial$ or design$ or protocol$)).mp.	0
5	master protocol$.mp.	1
6	response adaptive randomi$.mp.	0
7	(single centre or single site).mp.	129
8	(or/1-6) not 7 [adaptive design - not single-site]	22
9	(international adj5 (trial or trials or protocol$ or multicent$ or multi-cent$ or multisite or multi-site or multi-arm or multiarm or multi-stage or multistage)).mp.	114
10	(international clinical$ trial$ regist$ or international clinical$ trial$ platform$ or international standard randomi$ control$ trial$ number$).mp.	29
11	international.mp. /freq=2	3011
12	9 not (10 not 11) [gets rid of results with 'international trial' that only refer to the WHO ICTRP]	90
13	((multinational or multi-national or intercontinental or inter-continental or pan-europ$) adj8 (trial or trials or protocol$ or multicent$ or multi-cent$ or multisite or multi-site)).mp.	17
14	((trial or trials or protocol$) and (((multicent$ or multi-cent$ or multisite or multi-site) and sites) or centres or centers) and countries).mp.	26
15	((UK or United Kingdom) and (EU or Europ$) and (trial$ or protocol$)).mp.	113
16	(europe/ or albania/ or alps/ or andorra/ or austria/ or balkans/ or baltic countries/ or belarus/ or belgium/ or bosnia herzegovina/ or bulgaria/ or caucasus/ or central europe/ or croatia/ or cyprus/ or czechoslovakia/ or danube river/ or eastern europe/ or france/ or fyr macedonia/ or germany/ or gibraltar/ or greece/ or hungary/ or iberian peninsula/ or italy/ or liechtenstein/ or luxembourg/ or malta/ or mediterranean/ or moldova/ or monaco/ or montenegro/ or netherlands/ or papal states/ or poland/ or "republic of ireland"/ or romania/ or russia/ or san marino/ or scandinavia/ or serbia/ or slovenia/ or soviet union/ or switzerland/ or turkey/ or ukraine/ or yugoslavia/) and united kingdom/ and (trial$ or protocol$).mp.	22
17	(exp africa/ or exp asia/ or exp middle east/ or exp americas/ or exp oceania/) and exp europe/ and (trial$ or protocol$).mp.	64
18	or/12-17 [main international trial requirement]	303
19	methodology/	153
20	exp research strategies/	16557
21	challenge$.mp.	14704
22	(((trial$ or design) adj5 efficiency) or efficiencies).mp.	270
23	((complexit$ or guidance or guidelines or recommendations or considerations or issues or obstacles or barriers) and trial$).ti.	87
24	((lesson or lessons or pitfall or pitfalls) and (design$ or plan$)).mp.	1351
25	(trial$ adj4 (manag$ or run or running or conducting)).mp.	208
26	(design$ or rationale$ or implement$ or methodol$).ti.	7553
27	management/ or business management/ or corporate management/ or development management/ or joint management/ or office management/ or operational management/ or process management/ or programme management/ or project	9141

	management/ or quality management/ or research management/ or strategic management/ or team management/ or work organisation/ or administration/ or leadership/ or management communication/ or management operations/ or management planning/ or management practice/ or management process/ or management techniques/	
28	contract management/ or facilities management/ or financial management/ or human resources management/ or information management/ or knowledge management/ or materials & supplies management/ or physical distribution management/ or resource management/ or risk management/	10733
29	(((good or best) adj3 practice$) and trial$).ti.	13
30	(trial$ adj2 (authoris$ or authoriz$)).mp.	1
31	or/19-30	55720
32	((trial$ adj5 insur$) or insuring or indemni$).mp.	171
33	((add$ or remov$) adj5 (arm or arms)).mp.	7
34	gdpr.mp.	16
35	exp ethics/ or ethic$.ti.	4861
36	ethics committees/	135
37	((data adj8 secur$) or (data adj3 (share$ or sharing)) or data management or data governance).mp.	839
38	(trial$ adj3 infrastructure).mp.	1
39	statistical model/ or statistical analysis/ or statistical design/ or (statistical adj1 design$).mp.	551
40	software.mp.	1768
41	reporting.ti. or (reporting adj4 (quality or guideline$)).mp.	1107
42	(recruit$ adj4 (effective$ or model$ or pause$ or pausing)).mp. or recruit$.ti.	1058
43	(decision$ adj2 algorithm$).mp.	10
44	sponsor$.ti.	190
45	fund$.ti.	5155
46	exp financing/ or (financ$ not incentiv$).ti.	16452
47	resourcing.mp.	358
48	economic evaluation/ or economic.ti.	3423
49	(trial$ adj1 cost$).mp. or (costs or costing).ti.	3347
50	contract research org$.mp.	4
51	(regulations or (regulat$ adj3 framework$)).mp. or drug regulations/ or regulations/	6548
52	((legal$ or law or laws or legislat$ or policy or policies or rule or rules) adj5 (UK or United Kingdom or brit$ or EU or Europe$ or framework$ or national or government$)).mp.	14895
53	(clinical trial regulation or "536/2014").mp.	3
54	((EU or Europe or European) adj4 representative$).mp.	25
55	collaborat$.ti.	1607
56	(harmonis$ or harmoniz$).mp.	377
57	brexit.mp.	193
58	(safety adj3 (monitor$ or governance)).mp. or drug safety/	191
59	(((intervention or IMP or investigational medicin$ or drug$) adj6 (supply$ or inventory or export$ or import$)) or procur$).mp.	2378
60	(treatment selection or site selection).mp.	27
61	workload$.mp.	3445
62	exp contracts/ or (contract or contracts).mp.	7573
63	((fund$ or collaborat$ or delegat$ or site or sites or research or sponsor$) adj5 (agreement or agreements)).mp.	112
64	((operational or practical or legal or administrative or financial or procedural or ethical or methodolog$ or statistical or recruitment or sponsor$ or logistic$ or design) adj4 (complexit$ or guidance or guidelines or recommendations or considerations or issues or challenges or obstacles or barriers or difficulties or advantages or disadvantages)).mp.	5882
65	or/32-64	71491
66	(8 or 18) and (31 or 65)	186
67	limit 66 to yr="2006 -Current"	110
68	limit 67 to english	110
69	68 not systematic review.ti.	92

2 DATA EXTRACTION TEMPLATE

Data was extracted on the elements listed in Table S1 for all identified studies.

Table S1. Data extracted from included studies

- First author
- Publication year
- Study type
- Trial acronym
- Trial phase
- Blinding status
- Trial status
- Start date
- End date
- Number and location of sites
- Total sample size
- What condition was the study on?
- Type of intervention
- Number of arms and interventions in each arm
- What is the primary outcome?

Sponsorship
- Type of sponsor
- Was it the same sponsor across all sites?
- Details of additional sponsorship

Funding
- Main funding source
- Details of what was funded
- Did they fund all sites?
- Any funding challenges or solutions?
- Insurance details
- Was there a standard operating procedure?

Regulatory
- Were any guidelines used in oversight, if so what were they?
- Was there an EU representative?
- Details of GDPR
- Details of trial monitoring and oversight
- Details of auditing
- Were site specific documents prepared?

Trial management
- Name of randomisation system
- Process of adding and removing arms
- Process of adding and removing sites
- How were interventions chosen?
- Was there a trial steering committee present?
- Recruitment challenges and solutions
- Details of protocol amendments
- Staff training
- Name of data management system
- Any reported data management issues?

Intervention
- Who supplied the intervention?
- Was it the same suppler across all sites?
- Details of procurement and distribution
- Were pharmacies/pharmacists involved?
- How were adverse events monitored?
- Details of intervention licensing

Biospecimens
- What specimens were collected?
- Were they collected at all sites?
- Where were specimens processed?
- Details of specimen transport between sites

Agreements
- Was a data sharing agreement present?
- Who was the agreement between?
- Were site agreements present?
- Details of agreement challenges and solutions
- Who sought ethics approval?
- Details of ethics approval
- What was the contractual responsibility of site leads and stakeholders?
- Were materials translated?
- Details of translation and communication between sites

3 SUMMARY OF INCLUDED STUDIES

Table S2. Summary of included studies

First author (year)	Trial name	Trial design	Population	Number of sites	Site locations	Main coordinating centre(s)	Total participants	Intervention	Primary outcome	Enrolment period
Aban (2008)[25]	MGTX	Parallel	Myasthenia gravis	79	Global	USA	126	Other	Effectiveness	June 2006 – December 2015
Aitken (2008)[32]	PROMOTION	Parallel	Coronary artery disease	5	North America, Oceania	USA	3522	Behavioural	Prevention	February 2001 – June 2006
Angus (2020)[33]	REMAP-CAP	Adaptive-platform	Severe pneumonia and COVID-19		UK/EU, North America, Oceania, Asia	Australia, Thailand		Drug	Effectiveness	April 2016 – present
Aryal (2021)[38]	REMAP-CAP	Adaptive-platform	Severe pneumonia and COVID-19		UK/EU, North America, Oceania, Asia	RCC in Australia and Thailand		Drug	Effectiveness	April 2016 – present
Antic (2015)[42]	SAVE	Parallel	Obstructive sleep apnoea	89	Oceania, North America, South America, Asia, UK/EU	RCC in Australia, Brazil, China, India and Spain	2717	Device	Prevention	September 2008 – December 2015
Babiker (2013)[18]	START	Parallel	HIV	237	North America, South America, UK/EU, Oceania, Africa	RCC in Denmark, UK, Australia, USA	4000	Drug	Efficacy	April 2009 – July 2022
Berthon-Jones (2015)[26]	ALTAIR	Parallel	HIV	36	Asia, Oceania, UK/EU, North America, South America		322	Drug	Effectiveness	February 2007 – November 2011
Bryant (2021)[49]	TBTC Study 31	Parallel	Tuberculosis	34	North America, South America, Asia, Africa	USA	2516	Drug	Effectiveness	January 2016 – May 2021
Carli (2013)[43]	SEYLE	Cluster	Suicide	11	UK/EU	Sweden	11110	Behavioural	Prevention	September 2009 – January 2012
Clasen (2020)[53]	HAPIN	Parallel	Low birth weight		Asia, North America, South America, Africa	USA		Device	Prevention	September 2017 – present
Coomarasamy (2016)[50]	PROMISE	Parallel	Recurrent miscarriage	45	UK/EU	UK	836	Drug	Efficacy	June 2008 – May 2012
Crow (2018)[19]	FOR-DMD	Parallel	Duchenne muscular dystrophy	40	North America, UK/EU	USA	196	Drug	Effectiveness	January 2013 – November 2019
del Álamo (2022)[20]										
Denholm (2022)[51]	ASCOT ADAPT	Adaptive-platform	COVID-19		Oceania, Asia			Drug	Effectiveness	February 2021 – present
Dutton (2009)[44]	CONTROL	Parallel	Trauma	75	North America, South America, UK/EU, Asia, Africa	USA	576	Drug	Efficacy	October 2005 – September 2008
Eikelboom (2022)[39]	ACT	Factorial	COVID-19	62	North America, South America, Africa, Asia		6528	Drug	Effectiveness	April 2020 – February 2022
Fogelholm (2017)[45]	PREVIEW	Factorial	Pre-diabetes	8	UK/EU, Oceania		2326	Behavioural	Prevention	June 2013 – December 2018
Franciscus (2014)[52]	TRIGR	Parallel	Type 1 diabetes	77	North America, Oceania, UK/EU	USA	5156	Other	Prevention	May 2002 – December 2006
Fulda (2023)[48]	REPRIEVE	Parallel	HIV		North America, South America, Africa, Asia, UK/EU	USA		Drug	Prevention	March 2015 – present
Goossens (2021)[27]	REMAP-CAP	Adaptive-platform	Severe pneumonia and COVID-19		UK/EU, North America, Oceania, Asia	Australia, Thailand		Drug	Effectiveness	April 2016 – present

Garup (2015)[31]	START	Parallel	HIV	237	North America, South America, UK/EU, Oceania, Africa	RCC in Denmark, UK, Australia, USA	4000	Drug	Efficacy	April 2009 – July 2022
Hata (2020)[40]	PATHWAY	Parallel	Breast cancer	23	Asia		185	Drug	Efficacy	February 2018 – July 2022
Herrick (2012)[34]	FDTT	Parallel	Functional dyspepsia	8	North America		292	Drug	Efficacy	October 2006 – July 2013
Jeon (2016)[46]	CLEAR III	Parallel	Intracerebral haemorrhage	73	North America, South America, UK/EU, Asia	USA	500	Drug	Efficacy	September 2009 – January 2015
Kenyon (2011)[21]	STICH II	Parallel	Intracerebral haemorrhage	126	North America, Oceania, UK/EU, Asia, Africa	UK	601	Other	Efficacy	January 2007 – August 2015
Kesho Bora Study Group (2011)[35]	Kesho-Bora	Parallel	HIV	5	Africa	Switzerland	824	Drug	Efficacy	June 2005 – August 2008
Kolitsopoulos (2013)[47]	ZODIAC	Parallel	Schizophrenia	226	North America, South America, UK/EU, Asia		18240	Drug	Effectiveness	February 2002 – April 2007
Larson (2016)[22]	INSIGHT trials					RCC in UK, Denmark, USA and Australia				
Lingor (2021)[23]	ROCK-ALS / ROCK-ALS-US	Parallel	Amyotrophic lateral sclerosis		North America, UK/EU			Drug	Efficacy	February 2019 – present
Minisman (2012)[5]	MGTX	Parallel	Myasthenia gravis	79	Global	USA	126	Other	Effectiveness	June 2006 – December 2015
Murray (2022)[24]	TICO	Adaptive-platform	COVID-19		North America, UK/EU, Asia, Africa	USA with 8 RCC		Drug	Effectiveness	August 2020 – present
Neaton (2010)[9]	INSIGHT trials					RCC in UK, Denmark, USA and Australia				
Ravinetto (2013)[36]	4ABC	Parallel	Malaria	12	Africa	Belgium	4112	Drug	Efficacy	July 2007 – December 2009
Reams (2018)[41]	DOVE	Parallel	Sickle cell disease	51	North America, South America, UK/EU, Asia, Africa		341	Drug	Efficacy	April 2013 – December 2015
Seal (2006)[28]		Factorial	Endophthalmitis	24	UK/EU, Asia	UK	35000	Drug	Efficacy	September 2003 – May 2006
Spencer (2012)[54]	AWARD-5	Adaptive	Type 2 diabetes	111	North America, UK/EU, Asia		1202	Drug	Safety	August 2008 – July 2012
Sydes (2011)[29]	STAMPEDE	Adaptive-platform	Prostate cancer		UK/EU	UK		Drug	Efficacy	July 2005 – present
Zimmer (2010)[37]	BAMSG 3-01	Parallel	Cryptococcal meningitis	13	North America, Asia	USA	143	Drug	Efficacy	May 2005 – April 2008

RCC: Regional Coordinating Centre; RCT: Randomised Controlled Trial.

4 OPERATIONAL COMPLEXITIES REPORTED BY PUBLICATION

Table S3. Operational complexities in conducting international trials

Major barriers	Source
**Study set-up**	
Sponsorship, insurance and need for EU legal representative	Aban (2008); Babiker (2013); Berthon-Jones (2015); Crow (2018); del Álamo (2022); Goossens (2021); Kenyon (2011); Lingor (2021); Minisman (2012); Murray (2022); Neaton (2010); Seal (2006); Sydes (2011)
Funding	Aban (2008); Aitken (2008); Angus (2020); Babiker (2013); Crow (2018); del Álamo (2022); Goossens (2021); Herrick (2012); Kenyon (2011); Kesho Bora Study Group (2011); Larson (2016); Lingor (2021); Ravinetto (2013); Zimmer (2010)
Lack of harmonisation in ethics and regulatory approvals	Aban (2008); Aitken (2008); Aryal (2021); Babiker (2013); Berthon-Jones (2015); del Álamo (2022); Eikelboom (2022); Goossens (2021); Grarup (2015); Hata (2020); Kenyon (2011); Minisman (2012); Murray (2022); Neaton (2010); Ravinetto (2013); Reams (2018); Zimmer (2010)
**Site set-up**	
Training	Aitken (2008); Antic (2015); Aryal (2021); Carli (2013); Crow (2018); Dutton (2009); Fogelholm (2017); Jeon (2016); Kesho Bora Study Group (2011); Kolitsopoulos (2013); Minisman (2012)
Contracts	Babiker (2013); Crow (2018); del Álamo (2022); Goossens (2021); Grarup (2015); Larson (2016); Lingor (2021); Minisman (2012)
Site monitoring	Angus (2020); Aryal (2021); Bryant (2021); Carli (2013); Coomarasamy (2016); Denholm (2022); Dutton (2009); Franciscus (2014); Fulda (2023); Larson (2016); Ravinetto (2013); Seal (2006); Sydes (2011); Zimmer (2010)
Communication	Aitken (2008); Angus (2020); Antic (2015); Crow (2018); Denholm (2022); Fulda (2023); Herrick (2012); Minisman (2012)
Translation of materials	Aban (2008); Babiker (2013); Berthon-Jones (2015); Carli (2013); Crow (2018); Franciscus (2014); Lingor (2021); Zimmer (2010)
**Trial management**	
Trial oversight	Antic (2015); Babiker (2013); Coomarasamy (2016); Dutton (2009); Franciscus (2014); Herrick (2012); Larson (2016); Murray (2022); Spencer (2012)
Site and intervention selection	Angus (2020); Antic (2015); Coomarasamy (2016); Denholm (2022); Dutton (2009); Eikelboom (2022); Fulda (2023); Herrick (2012); Kesho Bora Study Group (2011); Kolitsopoulos (2013); Minisman (2012); Murray (2022); Reams (2018); Zimmer (2010)
Recruitment	Aitken (2008); Antic (2015); Berthon-Jones (2015); Eikelboom (2022); Franciscus (2014); Herrick (2012); Kesho Bora Study Group (2011); Kolitsopoulos (2013); Reams (2018)
**Data management**	Aitken (2008); Aryal (2021); Berthon-Jones (2015); Carli (2013); Coomarasamy (2016); Ravinetto (2013); Seal (2006)
**Intervention management**	
Drug procurement and distribution	Babiker (2013); Bryant (2021); Coomarasamy (2016); Crow (2018); del Álamo (2022); Goossens (2021); Grarup (2015); Hata (2020); Herrick (2012); Jeon (2016); Lingor (2021); Minisman (2012); Murray (2022); Ravinetto (2013); Reams (2018); Seal (2006); Spencer (2012); Zimmer (2010)
Pharmacy involvement	Aban (2008); Bryant (2021); Coomarasamy (2016); Herrick (2012); Jeon (2016); Kolitsopoulos (2013); Minisman (2012)
Monitoring safety and adverse events	Antic (2015); Bryant (2021); Coomarasamy (2016); Hata (2020); Herrick (2012); Minisman (2012); Murray (2022); Zimmer (2010)
Biospecimen processing and transport	Berthon-Jones (2015); Bryant (2021); Denholm (2022); Fogelholm (2017); Franciscus (2014); Fulda (2023); Hata (2020); Lingor (2021)
**Adaptive-specific**	Angus (2020); Aryal (2021); Denholm (2022); Murray (2022); Spencer (2012)

5 PRISMA CHECKLIST

Section and Topic	Item #	Checklist item	Location where item is reported
**TITLE**			
Title	1	Identify the report as a systematic review.	Page 1
**ABSTRACT**			
Abstract	2	See the PRISMA 2020 for Abstracts checklist.	Page 2
**INTRODUCTION**			
Rationale	3	Describe the rationale for the review in the context of existing knowledge.	Page 4
Objectives	4	Provide an explicit statement of the objective(s) or question(s) the review addresses.	Page 4
**METHODS**			
Eligibility criteria	5	Specify the inclusion and exclusion criteria for the review and how studies were grouped for the syntheses.	Page 5
Information sources	6	Specify all databases, registers, websites, organisations, reference lists and other sources searched or consulted to identify studies. Specify the date when each source was last searched or consulted.	Page 5
Search strategy	7	Present the full search strategies for all databases, registers and websites, including any filters and limits used.	Page 5
Selection process	8	Specify the methods used to decide whether a study met the inclusion criteria of the review, including how many reviewers screened each record and each report retrieved, whether they worked independently, and if applicable, details of automation tools used in the process.	Page 5, Figure 1
Data collection process	9	Specify the methods used to collect data from reports, including how many reviewers collected data from each report, whether they worked independently, any processes for obtaining or confirming data from study investigators, and if applicable, details of automation tools used in the process.	Page 5-6
Data items	10a	List and define all outcomes for which data were sought. Specify whether all results that were compatible with each outcome domain in each study were sought (e.g. for all measures, time points, analyses), and if not, the methods used to decide which results to collect.	Page 6
	10b	List and define all other variables for which data were sought (e.g. participant and intervention characteristics, funding sources). Describe any assumptions made about any missing or unclear information.	Page 6
Study risk of bias assessment	11	Specify the methods used to assess risk of bias in the included studies, including details of the tool(s) used, how many reviewers assessed each study and whether they worked independently, and if applicable, details of automation tools used in the process.	NA
Effect measures	12	Specify for each outcome the effect measure(s) (e.g. risk ratio, mean difference) used in the synthesis or presentation of results.	Page 5-6
Synthesis methods	13a	Describe the processes used to decide which studies were eligible for each synthesis (e.g. tabulating the study intervention characteristics and comparing against the planned groups for each synthesis (item #5)).	Page 5-6
	13b	Describe any methods required to prepare the data for presentation or synthesis, such as handling of missing summary statistics, or data conversions.	NA
	13c	Describe any methods used to tabulate or visually display results of individual studies and syntheses.	NA
	13d	Describe any methods used to synthesize results and provide a rationale for the choice(s). If meta-analysis was performed, describe the model(s), method(s) to identify the presence and extent of statistical heterogeneity, and software package(s) used.	Page 5-6
	13e	Describe any methods used to explore possible causes of heterogeneity among study results (e.g. subgroup analysis, meta-regression).	NA
	13f	Describe any sensitivity analyses conducted to assess robustness of the synthesized results.	NA
Reporting bias assessment	14	Describe any methods used to assess risk of bias due to missing results in a synthesis (arising from reporting biases).	NA
Certainty assessment	15	Describe any methods used to assess certainty (or confidence) in the body of evidence for an outcome.	NA
**RESULTS**			
Study selection	16a	Describe the results of the search and selection process, from the number of records identified in the search to the number of studies included in the review, ideally using a flow diagram.	Page 6-7
	16b	Cite studies that might appear to meet the inclusion criteria, but which were excluded, and explain why they were excluded.	Page 6-7 / Figure 1
Study characteristics	17	Cite each included study and present its characteristics.	Page 6-7 / Table 1
Risk of bias in studies	18	Present assessments of risk of bias for each included study.	NA
Results of individual studies	19	For all outcomes, present, for each study: (a) summary statistics for each group (where appropriate) and (b) an effect estimate and its precision (e.g. confidence/credible interval), ideally using structured tables or plots.	Table 2, Page 6-16

Section and Topic	Item #	Checklist item	Location where item is reported
Results of syntheses	20a	For each synthesis, briefly summarise the characteristics and risk of bias among contributing studies.	NA
	20b	Present results of all statistical syntheses conducted. If meta-analysis was done, present for each the summary estimate and its precision (e.g. confidence/credible interval) and measures of statistical heterogeneity. If comparing groups, describe the direction of the effect.	NA
	20c	Present results of all investigations of possible causes of heterogeneity among study results.	NA
	20d	Present results of all sensitivity analyses conducted to assess the robustness of the synthesized results.	NA
Reporting biases	21	Present assessments of risk of bias due to missing results (arising from reporting biases) for each synthesis assessed.	NA
Certainty of evidence	22	Present assessments of certainty (or confidence) in the body of evidence for each outcome assessed.	NA
**DISCUSSION**			
Discussion	23a	Provide a general interpretation of the results in the context of other evidence.	Page 13-15
	23b	Discuss any limitations of the evidence included in the review.	Page 15
	23c	Discuss any limitations of the review processes used.	Page 15
	23d	Discuss implications of the results for practice, policy, and future research.	Page 13-15
**OTHER INFORMATION**			
Registration and protocol	24a	Provide registration information for the review, including register name and registration number, or state that the review was not registered.	Page 5
	24b	Indicate where the review protocol can be accessed, or state that a protocol was not prepared.	NA
	24c	Describe and explain any amendments to information provided at registration or in the protocol.	NA
Support	25	Describe sources of financial or non-financial support for the review, and the role of the funders or sponsors in the review.	Page 3
Competing interests	26	Declare any competing interests of review authors.	Page 3
Availability of data, code and other materials	27	Report which of the following are publicly available and where they can be found: template data collection forms; data extracted from included studies; data used for all analyses; analytic code; any other materials used in the review.	

6 PROTOCOL

Operational best practice in international clinical trials: a systematic review protocol

Support and registration:
This systematic review has been funded by the NIHR (Application Accelerator Award reference number: NIHR153955). The funder will have no further role in any aspect of the review. This protocol has been registered with the Open Science Framework (registration DOI: https://doi.org/10.17605/OSF.IO/YVTJB) (1).

Aim:
The aim of this systematic review is to identify models of best practice and lessons learned from the set up and delivery of international clinical trials, specifically in regard to management of cross-border regulatory and logistical requirements.

Rationale:
Research practices have transformed over the last decade, evolving from large single centred trials to more global trials that promote international collaboration. International trials offer numerous advantages over single-centre including reduced operational costs particularly in developing countries, faster recruitment and expand the horizon for the availability of treatments. Despite their benefits, the process of expanding to multiple sites raises logistical challenges due to the diversity in laws, ethics, guidelines and regulations between countries.

Search strategy:
The search strategy will be designed in collaboration with an experienced information specialist and will be peer-reviewed by another information specialist using the PRESS checklist. Thesaurus headings and keywords have been used as appropriate, and the search will be translated to other sources accordingly. The search strategy will cover four areas: "international trials", "adaptive design trials", "study design", and "specific challenges" and requirements of designing and running trials. Multiple approaches to combining these aspects will be used to achieve a practical quantity of specific, relevant results, while also mitigating risks of missing relevant material. A wide range of subject headings and search terms in appropriate fields will be used for all concepts in the search, including proximity searching to cover the many variations on phrases applicable to these topics. We will also apply a date filter to include studies published after 2005 as international trials have become prominent only in the last 15-20 years. We will search Medline, Embase and Health Management Information Consortium (HMIC) for appropriate studies.

Participants or population:
As the focus is on the implementation and infrastructure of the trial, any clinical population and any condition will be considered of interest.

Intervention:
Clinical trials on any drug, device or therapeutic intervention will be considered if they are multi-centre and trial site locations are in more than one country.

Comparator:
Trials will be eligible regardless of comparator.

Study designs to be included:
Any multi-centre randomised trial design with sites in at least two different countries will be included.

Eligibility criteria:
The following types of studies will be included:

1. Any study reporting operational challenges to conducting international trials (involving two or more countries)
2. Available in English
3. Published after 2005

Conference abstracts, systematic reviews and protocols will be excluded.

Main outcome:
Any outcomes related to the best practice models and lessons learned in aspects of set up and delivery of international trials.

We will specifically look at:

- Sponsorship and funding: balancing UK, EU and other international trial regulations, policies and procedures in sponsorship of the overall trial
- Trial management structure: legal, ethical and regulatory matters, safety and monitoring standards, processes for adding and/or removing trial arms, GDPR and clinical data management system requirements
- Intervention management: pharmacy involvement, procurement, distribution, delivery of interventions

- Contracting: agreements of collaboration, sites, delegation, sample transfer logistics arrangements and data sharing.

Data management:
Zotero will be used to manage the studies throughout the review. Rayyan will be used to screen records.

Strategy for data synthesis:
Two reviewers will independently screen the titles and abstracts of the studies retrieved by the search. For studies deemed eligible, or studies where it is impossible to decide eligibility from the abstract, the full text will be retrieved, and two reviewers will independently assess for inclusion. Any disagreements will be resolved through discussion or if necessary, by reference to a third reviewer.

Data extraction will be undertaken by one reviewer and checked by a second, with discrepancies resolved by consultation with a third. Where studies are reported in multiple publications, we will extract relevant data from all publications but consider as one study. Where data is missing or unclear, we will contact authors to request details or clarification. The following data will be extracted from included studies:

- Citation information
- Study design variables: sample size, phase
- Study population and objective variables: age, condition, experimental interventions, primary outcome
- Regulatory information: status, location, availability of results, start and completion date
- Outcome data: Sponsorship information; funding information; trial management process information; intervention/pharmacy process information; detail on the contracting processes

In the first instance, we will present a summary of study characteristics and outcome data in a series of structured tables to give a clear picture of the available evidence.

Language:
Only studies published in English will be included.

References

1. Gumber L, Pratt A, Bardgett M, Inskip A, Still M, Phillipson J, et al. Operational best practice in international clinical trials: a systematic review protocol 2023. osf.io/yvtjb.
