# Supplementary material for: Operational complexities in international clinical trials: a systematic review of challenges and proposed solutions
Source: BMJ Open. 2024 Apr 15;14(4):e077132. doi: 10.1136/bmjopen-2023-077132 (PMC11029458; doi:10.1136/bmjopen-2023-077132)
Supplement: Author's manuscript [file bmjopen-2023-077132.draft_revisions.pdf]

BMJ Open is committed to open peer review. As part of this commitment we make the peer review history of every article we publish publicly available.

When an article is published we post the peer reviewers' comments and the authors' responses online. We also post the versions of the paper that were used during peer review. These are the versions that the peer review comments apply to.

The versions of the paper that follow are the versions that were submitted during the peer review process. They are not the versions of record or the final published versions. They should not be cited or distributed as the published version of this manuscript.

BMJ Open is an open access journal and the full, final, typeset and author-corrected version of record of the manuscript is available on our site with no access controls, subscription charges or pay-per-view fees (http://bmjopen.bmj.com).

If you have any questions on BMJ Open's open peer review process please email info.bmjopen@bmj.com

BMJ Open

Operational complexities in international clinical trials: a systematic review of challenges and proposed solutions

Journal:	*BMJ Open*
Manuscript ID	bmjopen-2023-077132
Article Type:	Original research
Date Submitted by the Author:	26-Jun-2023

Complete List of Authors:	Gumber, Leher; Newcastle University, Translational and Clinical Research Institute; Gateshead Health NHS Foundation Trust,<br>Agbeleye, Opeyemi; Newcastle University, Population Health Sciences Institute<br>Inskip, Alex; Newcastle University, Population Health Sciences Institute<br>Fairbairn, Ross; Newcastle University, Population Health Sciences Institute<br>Still, Madeleine; Newcastle University<br>Ouma, Luke; Newcastle University, Population Health Sciences Institute<br>Lozano-Kuehne, Jingky; Newcastle University, Population Health Sciences Institute<br>Bardgett, Michelle; Newcastle University<br>Isaacs, John; Newcastle University, Translational and Experimental Medicine Institute; Newcastle Upon Tyne Hospitals NHS Foundation Trust, Musculoskeletal Unit<br>Wason, James; Newcastle University, Population Health Sciences Institute; University of Cambridge, MRC Biostatistics Unit<br>Craig, Dawn; Newcastle University, Health Economics Group, Institute of Health and Society<br>Pratt, Arthur; Newcastle University; Newcastle Upon Tyne Hospitals NHS Trust

Keywords:	Clinical Trial, Randomized Controlled Trial, Systematic Review

SCHOLARONE™ Manuscripts

I, the Submitting Author has the right to grant and does grant on behalf of all authors of the Work (as defined in the below author licence), an exclusive licence and/or a non-exclusive licence for contributions from authors who are: i) UK Crown employees; ii) where BMJ has agreed a CC-BY licence shall apply, and/or iii) in accordance with the terms applicable for US Federal Government officers or employees acting as part of their official duties; on a worldwide, perpetual, irrevocable, royalty-free basis to BMJ Publishing Group Ltd ("BMJ") its licensees and where the relevant Journal is co-owned by BMJ to the co-owners of the Journal, to publish the Work in this journal and any other BMJ products and to exploit all rights, as set out in our licence.

The Submitting Author accepts and understands that any supply made under these terms is made by BMJ to the Submitting Author unless you are acting as an employee on behalf of your employer or a postgraduate student of an affiliated institution which is paying any applicable article publishing charge ("APC") for Open Access articles. Where the Submitting Author wishes to make the Work available on an Open Access basis (and intends to pay the relevant APC), the terms of reuse of such Open Access shall be governed by a Creative Commons licence – details of these licences and which Creative Commons licence will apply to this Work are set out in our licence referred to above.

Other than as permitted in any relevant BMJ Author's Self Archiving Policies, I confirm this Work has not been accepted for publication elsewhere, is not being considered for publication elsewhere and does not duplicate material already published. I confirm all authors consent to publication of this Work and authorise the granting of this licence.

For peer review only

Operational complexities in international clinical trials: a systematic review of challenges and proposed solutions

Leher Gumber[1,2], Opeyemi Agbeleye[3], Alex Inskip[3], Ross Fairbairn[3], Madeleine Still[3], Luke Ouma[4], Jingky Lozano-Kuehne[4], Michelle Bardgett[5], John D Isaacs[1,6], James Wason[4], Dawn Craig[3*] and Arthur G Pratt[1,6*].

[1]Translational and Clinical Research Institute, Newcastle University, UK.
[2]Gateshead Health NHS Foundation Trust, UK.
[3]NIHR Innovation Observatory, Population Health Sciences Institute, Newcastle University, UK.
[4]Population Health Sciences Institute, Newcastle University, UK
[5]Newcastle Clinical Trial Unit, Newcastle University, UK.
[6]Musculoskeletal Unit, Newcastle upon Tyne Hospitals NHS Trust, UK.

*Denotes equal contribution

Corresponding author:
Dr Arthur G Pratt
Senior Clinical Lecturer and Consultant Rheumatologist
Translational and Clinical Research Institute
Newcastle University
Newcastle upon Tyne
NE1 7RU, UK
Email: arthur.pratt@newcastle.ac.uk

For peer review only

Abstract

Objective: International multi-centre trials are increasingly used in clinical research but can be challenging to operationalise due to incompatibilities between country-specific policies and infrastructures. The aim of this systematic review was to identify the operational complexities of conducting international clinical trials and identify potential solutions for overcoming them.

Design: Systematic review.

Data sources: Medline, Embase and Health Management Information Consortium were searched from 2006 to 30th January 2023.

Eligibility criteria: Any publication reporting operational challenges (e.g. site selection, trial management, intervention management, data management) of conducting international trials were included.

Data extraction and synthesis: Search results were independently screened by at least two reviewers and data were extracted into a pre-designed proforma.

Results: 38 studies (35 RCTs, 2 reports and 1 qualitative study) fulfilled the inclusion criteria. The median sample size was 1202 (IQR 332 – 4056) and median number of sites was 40 (IQR 13 – 78). 88.6% of studies had an academic sponsor and 80% were funded through government sources. Operational complexities were particularly reported during trial set-up due to lack of harmonisation in regulatory approvals and in relation to sponsorship structure, with associated budgetary impacts. Additional challenges included site selection, staff training, lengthy contract negotiations, site monitoring, communication, trial oversight, recruitment, data management, drug procurement and distribution, pharmacy involvement and biospecimen processing and transport.

Conclusions: International collaborative trials are valuable in cases where recruitment may be difficult, diversifying participation and applicability. However, multiple operational and regulatory challenges are encountered when implementing a trial in multiple countries. Careful planning and communication between trials units and investigators, with an emphasis on establishing adequately resourced cross-border sponsorship structures and regulatory approvals, may help to overcome these barriers and realise the benefits of the approach.

Open Science Framework registration number: osf-registrations-yvtjb-v1.

Strengths and limitations:

- Key strengths of this study include: a robust search strategy and screening of abstracts by at least two reviewers
- We focused on international trials so findings may not be generalisable to multi-centre studies conducted within the same country

Word count: 3999

Funding: This study was funded by National Institute of Health and Care Research (NIHR153955). Infrastructural support was provided by the National Institute of Health and Care Research (NIHR) Newcastle Biomedical Research Centre. The views expressed are those of the author(s) and not necessarily those of the NIHR or the Department of Health and Social Care.

Competing interests statement: None declared.

For peer review only

Contribution of authors: LG was involved in conception of the study, screening, data extraction, analysis and writing up the manuscript. OA was involved in screening and writing up the manuscript. AI was involved in developing search strategy and revising it critically for important intellectual content. RF, MS were involved in screening and revising it critically for important intellectual content. MB was involved in developing search strategy and revising it critically for important intellectual content. LO, JLK, JDI, JW and DC revised the manuscript for important intellectual content. AGP was responsible for project oversight and revised the manuscript for important intellectual content.

Acknowledgements: We would like to thank Chris Price, Andrew Johnston, Julia Philipson and Ruth Wood for providing insights into outcome measures.

For peer review only

Introduction

Research practices have transformed over the last decade, evolving from single-centred and within-nation multi-centre trials to include multi-national or global trials that require international collaboration. Several factors have contributed to this shift, including advancements in technology, globalisation and insufficient accrual rates with traditional trial designs[1]. The COVID-19 pandemic has been a further catalyst, giving rise to several international trials employing innovative and/or adaptive designs. For example, the Solidarity PLUS trial was launched by the World Health Organization to enable rapid evaluation of COVID-19 treatments and recruited participants from 52 countries[2].

International trials offer numerous advantages over single-nation randomised controlled trials (RCTs). They increase access to potentially eligible participants, enable faster recruitment and/or larger sample sizes whilst encouraging representation of diverse ethnic, biological and socio-cultural groups[1,3–5]. International trials promote best clinical practices globally and expand the horizon for the availability of treatments in countries that may not otherwise have access to a drug or intervention[5]. The reduced operational cost of running a trial in developing compared to developed countries may be an additional consideration in some cases[6]. Furthermore, they foster collaboration and development of closer relationships among academics[1,3,5,7,8]. Successful conduct of international trials requires adherence to local and international laws, regulations and ethical requirements, availability of Good Clinical Practice (GCP)-trained researchers, adequate infrastructure at clinical sites, and close monitoring and oversight across multiple jurisdictions[5,9,10]. Incompatibilities between country-specific policies and challenges in the trial setup were previously identified as factors delaying study start times, extending timelines and inflating costs[11]. Other contributing factors including site selection, insurance, logistics, regulatory requirements and sponsorship, have been highlighted in a narrative review [12]. Trials conducted under a master protocol can present further challenges, adaptations that may, in turn, incur ethical and/or regulatory review, sample size re-estimations, site capacity and data management challenges[13,14]. In recognition of these issues and a lack of best practice guidelines, we conducted a systematic review to identify: (1) the operational complexities of conducting international clinical trials and (2) potential solutions for overcoming practical challenges.

For peer review only

Methods

This study was reported in accordance with the Preferred Reporting Items for Systematic Reviews and Meta-analyses (PRISMA) guidelines[15]. The study was registered with the Open Science Framework (Identifier: osf-registrations-yvtjb-v1)[16] and funded by National Institute of Health and Care Research (NIHR153955).

Search strategy and selection criteria

A search strategy was developed in collaboration with an information specialist (AI) using a combination of key words and MeSH terms. The search covered four broad areas: (i) international trials; (ii) adaptive design trials; (iii) study design; (iv) specific challenges and requirements of designing and running trials (Appendix 1, Supplement). Multiple approaches to combining these elements were iterated to achieve a pragmatic quantity of relevant results, whilst mitigating the risk of omitting relevant material. Medline and Embase were the primary bibliographic database sources, but a variation of the search was also run on the Health Management Information Consortium (HMIC) database.

Selection of studies

Ovid was used to de-duplicate records and Zotero (reference management tool) was used to manage the studies throughout the review. Rayyan (Qatar Computing Research Institute), a systematic review web-based application, was used for screening. Studies were included if they reported operational challenges for conducting international trials (sites in two or more countries), published after 2005 and in English. This decision was made as studies conducted prior to 2006 are less likely to be reflective of current legislations or include adaptive designs. Conference abstracts, protocols, systematic reviews and studies not available in English were excluded. Titles and abstracts were double screened by at least two reviewers (from LG, MS, OA and RF). For studies deemed eligible, or where it was deemed impossible to decide eligibility from the abstract, the full text was retrieved. Full-text articles were screened for inclusion by LG or OA. 20% of the full-text articles underwent screening by a second reviewer (either MS or RF). In case of discrepancy, the decision was taken by consensus of all the reviewers.

Data extraction

For peer review only

Data were extracted using a pre-designed proforma by LG and checked by OA with discrepancies resolved by consultation with an additional reviewer. Due to the nature of the review, a formal quality assessment of included studies was not performed. The following data were extracted: citation information, study type, study population, number of sites and objective variables (disease, sample size, intervention, primary outcome), regulatory information (status of trial, site locations, start and completion dates, details of sponsor and funder) and outcome data (trial management, intervention management, data management, selection of sites and interventions, training, monitoring and details on contracts).

Definition of variables and data analysis

The sponsor was determined as either academic (universities, hospitals or the government), or industry (pharmaceutical or device companies). The funder was defined as academic (university or hospital), government, industry or charity. Location of studies was categorised by continent into: UK/Europe (EU), Asia, Africa, North America, South America or Oceania. Primary outcome measures were considered as efficacy, effectiveness, prevention or safety. Quantitative data were described using numbers, percentages, median and interquartile range. Qualitative data were summarised into overarching themes which described the main challenges of conducting international trials.

For peer review only

Results

The search identified 5215 records, of which 1588 were duplicates and removed. After title and abstract screening of the remaining 3627 articles, a further 3374 were excluded. Full text screening was conducted on 253 articles from which 38 were included (Figure 1). The included studies have been summarised in Table 1.

Study characteristics

Of the 38 included studies, 35 (92.1%) were RCTs, 2 (5.3%) were reports and 1 (2.6%) was a qualitative study. Of the 35 RCTs, 24 (68.6%) had a parallel design, 7 (20.0%) were platform, 3 (8.6%) were factorial and 1 (2.9%) was cluster. Over half of the RCTs were open-label. Most RCTs evaluated a drug intervention (74.3%) and measured efficacy (40.0%) as their primary outcome. The median number of sites and sample size among closed trials was 40 (IQR 13 –

78) and 1202 (IQR 332 – 4056) respectively. The majority of studies (n=31, 88.6%) were sponsored by an academic institution and the most common funding source was government (80.0%). The continents most commonly represented by sites included in studies were North America (n=27, 77.1%), and UK/EU (n=25, 71.4%). The design characteristics of the included studies are summarised in Table 2. Operational complexities of conducting international trials were reported broadly at six stages of the trial process including: study set-up, site set-up, trial management, data management, intervention management and adaptive specific features (Figure 2). We structure out findings accordingly herein, summarising recurring themes and potential approaches used to address them in Table 3.

For peer review only

Study set-up

Sponsorship, insurance and need for EU legal representative

Thirteen studies described variations in sponsorship and insurance requirements between countries which created delays in trial start-up[9,17–29]. This was particularly notable for UK/EU sites. The EU Clinical Trials Directive which governs the conduct of clinical trials in EU, requires the presence of an EU legal representative for trials sponsored by a non-EU institution[30]. After identification of an EU legal representative, repeated negotiations were necessary to clarify roles and responsibilities between the sponsor and legal representative resulting in further delays[18–20,31]. Country-specific variations in insurance and indemnity requirements within the EU were a further hurdle. For example, two studies noted that the minimum compensation in Germany was 500,000 euros and the insurance provided by the sponsor did not meet these limits. Additional insurance coverage could not be provided locally and, consequently, those sites were either dropped or authors had to run two parallel trials in different continents[22,23].

Funding

Fourteen studies described funding as a challenge[18–23,25,27,32–37]. Interestingly, all of these were funded through government sources. Limitations in funding meant that some investigators had to seek additional sources of support and in cases where this was unsuccessful, there was a delay in trial start up[19,20,35,36]. Additionally, trials funded by the US had a requirement for non-US sites to obtain departmental clearance and approval from their respective countries before funds could be transferred. A lack of familiarity with this process prevented timely transfer of funds and thus delayed recruitment[25]. Predicting accurate budget projections and variations in

currency exchange rates were described as additional challenges particularly for trials spanning several years[32]. Moreover, prolonged negotiations between the funder, site and sponsor regarding site set-up costs led to further delays[31].

Lack of harmonisation in ethics and regulatory approvals

A lack of harmonisation in international legal and ethical systems was a reoccurring theme[9,17,18,20,21,24–27,31,32,36–41]. The time from initiation of regulatory procedures to the start of the trial was sometimes very long, ranging from 3 to 18 months. Contributing factors were: lack of a centralised system and therefore the requirement for single-centre approvals[5,18,21,23,25,32,39], country-specific differences in requirements[5,21,23,25,36,40,41], infrequent ethics committee meetings[21,33], ethics review fee[21,36], translation of essential documents[40], protocol amendments[35,36] and lack of familiarity among ethics committees with trial design or conduct[21,39]. Additionally, multinational trials funded in total or in part by US National Institute of Health required Federalwide Assurance (FWA) approvals and annual reviews alongside adherence to country-specific regulations. Although this process was familiar to US sites, non-US sites found this challenging[5,9,22,25].

Site set-up

Training

In general, staff training was not considered a challenge[17,19,32,35,38,42–47]. Studies used a combination of in-person and online workshops which covered a broad range of topics including: study protocol, Good Clinical Practice, data management, intervention delivery and safety reporting[19,25,33,45,46]. One trial adopted a system of "train-the-trainer," whereby site leads would receive centralised training then train all personnel at their local sites[43].

Contracts

Contracts were formed at many levels including site agreements, funding agreements, sponsorship agreements, agreements with pharmaceutical companies and data sharing. Several studies described contract negotiations as a time consuming and lengthy process[17–20,22,23,27,31]. Contributing factors to this were: translation of contracts[25], conflict with country-specific legal terminology and interpretation[19,20], clauses of indemnification[18,19] and administrative bureaucracy with legal departments[31]. Data sharing across borders was a further challenge requiring additional levels of consent and protection. Trials with EU sites described how all

For peer review only

participating sites were required to comply with the EU General Data Protection Regulations (GDPR)[20,23]. The GDPR requires anonymisation of all participant details by removing links between identifiers and codes and permits participants access to and deletion of the data at any time. This required additional administrative reviews to be set up[23]. To overcome these challenges, INSIGHT (International Network for Strategic Initiatives in Global HIV Trials) established coordinating centres in several countries that were geographically closer to sites, familiar with local regulatory requirements and fluent in local languages which improved the contract negotiation process[22].

Site monitoring

Monitoring and auditing of sites was conducted periodically and included checking for compliance with the protocol and standard operating procedures (SOPs), data consistency and missing data. Most studies adopted a combination of remote and in-person quality assurance visits and created summary reports that were circulated to sites[33,38,48–50]. Underperforming sites were asked to submit an action plan and provide additional staff training[48,50]. A clear escalation procedure was established for ensuring timely improvement of site performances[48]. Studies reported the importance of planning adequate human and financial resources for these tasks as conducting visits across multiple countries were costly and time consuming[28,36].

Communication

Regular communication with sites and investigators was key to successful trial implementation[17,19,32–34,42,48,51]. Multiple communication channels were utilised to ensure consistency across sites, overcome time zone and cross-cultural differences. These included emails for study protocols, newsletters, progress reports and site score cards, and regular verbal communication in the form of monthly site teleconferences, outreach calls with leadership team and biannual or annual face-to-face meetings[5,19,32,34,42,48,51]. Communications were usually managed by a contract research organization, clinical trials unit or the site selection committee[46,48]. One study described the guidelines they created to manage site queries. In the first instance, site staff were advised to contact their site lead with any questions. If unresolved, these could be forwarded to the coordinating centre and if needed the principal investigator. A similar process was adopted for response to queries whereby if the response was relevant to all participating sites, a general group email was circulated instead of an individual email. This infrastructure helped limit unnecessary or excessive email traffic[32].

For peer review only

Trial management

Trial oversight

A number of management "tiers" could be identified, according to their scope of responsibility for trial oversight. Most studies described a Central Coordinating Centre (also referred to as a "trial coordinating centre", "main operational centre" or "clinical or data coordinating centre"; CCC) with over-arching responsibility for overseeing trial setup and conduct in accordance with the protocol, standard operating procedures (SOPs) and regulatory approvals[9,17,22,24,25,36,37,43,46,48–51]. The CCC worked with at least one national coordinating centre (also referred to as an "country coordinating centre" or "national project manager") that supported the regulatory approvals, set up of sites and delivery of the trial in each country[18,24,31,42]. In trials spanning multiple continents, there was an additional level of management with an international coordinating centre that worked closely with the CCC and was responsible for oversight of several national coordinating centres[9,22,52]. This arrangement ensured that there were data and regulatory consistencies between countries or research networks[42,49–51]. In general, the CCC consisted of the chief investigator, biostatistician, and support staff and were responsible for oversight of the regulatory aspects, recruitment and retention issues[34], trial monitoring[18,31,43,46,49,50], secondary analyses and communication[9,22,34,48]. Other roles carried out by the CCC included overseeing finance and logistics including drug distribution, storage and analyses of specimen and community support[9,22,24]. Independent overall oversight of was provided by Data Safety Monitoring Board (or Committee; DSMB) and Trial Steering Committee (TSC) [51]. Most study reports referred to the International Conference on Harmonization Good Clinical Practice guidelines (ICH-GCP)[27,33,38,40,41,46,49,50].

Site and intervention selection

Site selection was often based upon initial feasibility assessments[41,44,47]. Several factors were considered during the assessment including local capacity or infrastructure[5,35,37,41,51], prevalence or burden of the disease in question[5,37,41], local ethics committees and regulatory processes[33,38,41,47], individual sites' interest in participating[5], level of expertise available, and insight from local sites representatives[48]. The choice of intervention was usually based on expert reviews[5,42] or a systematic literature review[24,50] which was overseen by the TSC. Some studies also considered treatment guidelines[18,31] and expert consensus where evidence was sparse[39].

For peer review only

Recruitment

Recruitment strategies varied across the studies. Factors contributing to the difficulty in enrolling and retaining participants were complex and lengthy screening processes[26,47], financial constraints[35], difficulty in maintaining long-term site cooperation[47] and the rigour of navigating varied health systems and setups across countries. Country-specific laws further hampered recruitment particularly in Germany where participants could only enrol in one trial at a time[31]. The reported strategies that helped to overcome these challenges were frequent communication with participants through a combination of letters, phone calls, mailing of local medical publications containing the trial details and increasing study visibility through the use of websites[32,34,47,52] as well as implementation of the intervention at a convenient location[32]. Furthermore, some studies organised educational sessions in the form of grand rounds and presentations to healthcare providers which increased visibility[32,34]. Reimbursement of transport costs and keeping visits to a minimum also increased recruitment[34,41].

Data management

Data management was not a major challenge and was generally overseen by the Data Coordinating Centre (DCC)[26,28,32,36,38,43,50]. Data was usually collected at trial sites using data collection forms and stored locally or in a web-based system such as Microsoft Access, JavaScript or SPSS. Studies which used the local storage model requested trial sites to securely send data to the DCC where it was amalgamated at regular intervals[32,36,43]. In contrast, use of a web-based system provided real time data entry, study updates and improved access[45,49,50,52]. Some studies reported technical challenges including problems with internet or firewalls, delays in data collection and missing data[32,38]. This was managed by ensuring sites had access to technical and training support[26,38].

Intervention management

Drug procurement and distribution

Pharmaceutical companies were the most common source of drug procurement[5,18,25,26,31,36,50,52], and in some cases the investigational product was supplied free of charge[18,31,52]. In instances where there were drug import restrictions, local pharmacies and suppliers were used[5,34]. Most studies established a central drug repository or clinical coordinating pharmacy to oversee the translation, labelling, repackaging, shipping, and coordination of the drug distribution to the study sites[18,23,24,31,37,46]. Where studies extended across multiple

For peer review only - http://bmjopen.bmj.com/site/about/guidelines.xhtml

continents, country-specific repository centres were created. These handled the different regulatory and import requirements in addition to providing oversight and support for the distribution of the investigational drugs across sites in their jurisdiction[18,31,46]. More often, studies were faced with difficulty in navigating complex regulatory procedures between countries. For example, some studies in the EU were refused waiver for labelling requirements of a repurpose drug whereas a similar waiver request was granted in the UK[18,19,27,31]. Also, there were country-specific differences in import requirements and issues around "qualified person" drug release specifications[18,50].

Pharmacy involvement

Site pharmacies were involved in drug dispensing in some studies[17,25,34,46,47,49,50]. SOPs and training were provided to hospital pharmacists at each study site on drug procurement, dispensing, storage, maintenance of accountability logs and disposal of unused or expired drugs[5,49,50].

Safety monitoring and adverse events

Serious and adverse events (SAEs) at sites were reported either directly to the CCC or through an electronic data capture or web-based system then to sponsor who reported to the relevant ethical and regulatory authority. One study ensured that a study coordinator, physician and safety officer were available at all times to manage emergency situations[34].

All 38 studies established a DSMB for monitoring drug safety and efficacy and safeguarding trial participants. SAEs were reviewed by the independent DSMB at varying intervals based on risk. [5,34,39,42]. The timing for These ranged from monthly, to biannually and annually. Some studies used a predetermined interval, for example, in one study the DSMB met every time at least 25-30 participants had a particular data point collected[24]. If there were concerns or SAEs, these meetings were brought forward. All monitoring activities were generally followed by reports which were circulated to all sites[24,51].

Biospecimen processing and transport

A variety of biospecimens were collected and these were often processed in a different country[26,45,49,52,53]. Several studies asked sites to ship the specimen to a designated central facility or repository for analysis[26,45,49,52,53]. Where trials spanned across multiple continents, separate central facilities were

For peer review only

established in each continent[23,40,53]. A central facility was used for specimen storage and if this was not feasible, a central virtual biobank was established[51]. The collection, handling and processing of specimens were performed in accordance with the SOPs[49,51]. Studies described challenges in the process including over-collection or under-collection of specimens, increased shipping costs for collection of additional samples, shipments delays, staff shortages and inadequate training[26,48]. As most of the specimens were shipped to another country for analysis, there were hurdles in getting the regulatory and administrative approvals, coupled with difficulty in securing informed consent from participants for their samples to be stored in another country[23,40]. The use of electronic inventory for real time monitoring of collected samples, ongoing laboratory training and efficient trial oversight were described as effective methods for overcoming these challenges.

Adaptive-specific issues

Adaptive trials described how, compared to traditional trials, significantly more documentation to provide clarity on the adaptive process was necessary, including treatment arms, plans for data collection and interim analyses[24,54]. Managing protocol amendments posed a further challenge. Studies reported that using an overarching master protocol and consent form and only including agent-specific information in the appendices significantly expedited the time for ethics approval when adding or removing treatment arms[24,51]. Handling drug supply for multiple treatment arms whilst minimising waste was another recurring theme. In order to overcome this, studies found it helpful to use pharmacies that could serve multiple clinical sites within a close geographical area, a centralised drug distribution system through 1-2 depots[24] and choosing countries with similar drug labelling requirements[54]. Data systems that enabled rapid data entry and analyses was another important consideration. Studies reported that setting up a streamlined system with pre-specified stopping rules for each treatment arm was helpful[33,51]. They also had predefined timelines for the frequency of data analysis [38,51,54].

Discussion

We conducted a systematic review to determine the operational complexities of conducting international trials. Our review highlights that there are various, consistent challenges in the planning, setup, delivery and close out phases of the 38 international trials. A lack of harmonisation within EU and between EU and other developed countries was

For peer review only

reported to be one of the greatest challenges. The EU Clinical Trials Regulation launched a portal in 2022 that enables registration of trials with sites in up to 30 EU countries in a single platform. This system also resolves data sharing issues between nations by providing a one-off consent for use of patients' data and enables national regulators to collaboratively process regulatory approvals[55]. However, there is no obligation for EU countries to participate until 2025 and this process does not provide a solution for collaboration with other continents. Furthermore, this portal does not extend to UK sites following its withdrawal from the EU. Possible interim solutions to global collaboration include defining responsibilities of sponsors and legal representatives, ascertaining insurance requirements prior to country selection and ensuring all sites are provided with a framework for the regulatory review process. In addition, working with a specialist insurance broker and careful country selection based on previous successful collaborations have also been suggested[56].

Budgetary constraints and contract negotiations were described as further hurdles. Over the last few years, several networks have been formed to increase funding opportunities. For example, INSIGHT was created to provide governance and funding support for multi-national HIV treatment trials but has expanded to also include influenza and COVID-19[57]. NeuroNEXT (Network for Excellence in Neuroscience Clinical Trials) promotes partnership in neurological disease trials by providing a centralised system for ethics review, contracting agreements and data management but is limited to multi-centre studies in the US[58]. Use of these networks, establishing site set-up costs during the trial planning phase and timely contract negotiations may assist in this process.

Managing drug distribution across international borders remains a challenge, as has also been previously reported[20]. Tailoring supply chains whilst minimising drug wastage is clearly desirable. Establishment of central drug repositories in each continent and using pharmacies which can serve multiple sites rather than the traditional one-pharmacy-one-site model has been shown to be effective[18,46]. Involving pharmaceutical partners in drug distribution is another possibility which also eliminates the need for time-consuming vendor selection[59].

Our study had several strengths. Our search strategy was robust and our data collection methods rigorous. Each abstract was reviewed by at least two authors for inclusion and 20% of the full-text articles were screened by a second author. Limitations include that our systematic review only focused on trials conducted in more than one country and therefore our results may not be generalisable to multi-centre studies conducted within the same

For peer review only

country. For example, ethics regulations, sponsor responsibilities and insurance requirements are less likely to be a hurdle in single nation trials[59]. Furthermore, not all investigators routinely publish information on challenges encountered in trial design and conduct. Further research in the form of a qualitative or Delphi study may provide more in-depth information on these challenges and possible actions to mitigate them.

Conclusion

International trials address many unmet needs in trial design, with adaptive designs affording the added potential to evaluate multiple treatments concurrently within one protocol. However, international trial investigators still face several operational challenges at every level. Hurdles that are consistently reported across the studies range from difficulties in obtaining regulatory approvals to site contracts and managing drug distribution. Funding can also prove difficult due to design complexity and lack of an integrated funding model between nations. This can create unnecessary delays in trial set-up. Careful planning and communication with sites and key stakeholders during the trial planning phase can overcome some of these challenges. More generally, given the upsurge in global trials particularly since the COVID-19 pandemic, recognition by policymakers of the potential rewards that regulatory harmonisation between nations could bring for the delivery of more efficient and cost-effective research should positively impact the health and wellbeing of their citizens. National and international organisations should continue to work collaboratively to develop infrastructures that support international trials.

For peer review only

Tables

Table 1. Included studies and their characteristics

First author (year)	Study type	Trial name	Population	Number of sites	Site locations	Total participants	Intervention	Primary outcome	Enrolment period
Aban (2008)[25]	RCT	MGTX	Myasthenia gravis	79	Global	126	Other	Effectiveness	June 2006 – December 2015
Aitken (2008)[32]	RCT	PROMOTION	Coronary artery disease	5	North America, Oceania	3522	Behavioural	Prevention	February 2001 – June 2006
Angus (2020)[33]	RCT	REMAP-CAP	Severe pneumonia and COVID-19		UK/EU, North America, Oceania, Asia		Drug	Effectiveness	April 2016 – present
Aryal (2021)[38]	RCT	REMAP-CAP	Severe pneumonia and COVID-19		UK/EU, North America, Oceania, Asia		Drug	Effectiveness	April 2016 – present
Antic (2015)[42]	RCT	SAVE	Obstructive sleep apnoea	89	Oceania, North America, South America, Asia, UK/EU	2717	Device	Prevention	September 2008 – December 2015
Babiker (2013)[18]	RCT	START	HIV	237	North America, South America, UK/EU, Oceania, Africa	4000	Drug	Efficacy	April 2009 – July 2022
Berthon-Jones (2015)[26]	RCT	ALTAIR	HIV	36	Asia, Oceania, UK/EU, North America, South America	322	Drug	Effectiveness	February 2007 – November 2011
Bryant (2021)[49]	RCT	TBTC Study 31	Tuberculosis	34	North America, South America, Asia, Africa	2516	Drug	Effectiveness	January 2016 – May 2021
Carli (2013)[43]	RCT	SEYLE	Suicide	11	UK/EU	11110	Behavioural	Prevention	September 2009 – January 2012
Clasen (2020)[53]	RCT	HAPIN	Low birth weight		Asia, North America, South America, Africa		Device	Prevention	September 2017 – present
Coomarasamy (2016)[50]	RCT	PROMISE	Recurrent miscarriage	45	UK/EU	836	Drug	Efficacy	June 2008 – May 2012
Crow (2018)[19]	RCT	FOR-DMD	Duchenne muscular dystrophy	40	North America, UK/EU	196	Drug	Effectiveness	January 2013 – November 2019
del Álamo (2022)[20]	Qualitative								
Denholm (2022)[51]	RCT	ASCOT ADAPT	COVID-19		Oceania, Asia		Drug	Effectiveness	February 2021 – present
Dutton (2009)[44]	RCT	CONTROL	Trauma	75	North America, South America, UK/EU, Asia, Africa	576	Drug	Efficacy	October 2005 – September 2008
Eikelboom (2022)[39]	RCT	ACT	COVID-19	62	North America, South America, Africa, Asia	6528	Drug	Effectiveness	April 2020 – February 2022
Fogelholm (2017)[45]	RCT	PREVIEW	Pre-diabetes	8	UK/EU, Oceania	2326	Behavioural	Prevention	June 2013 – December 2018
Franciscus (2014)[52]	RCT	TRIGR	Type 1 diabetes	77	North America, Oceania, UK/EU	5156	Other	Prevention	May 2002 – December 2006
Fulda (2023)[48]	RCT	REPRIEVE	HIV		North America, South America, Africa, Asia, UK/EU		Drug	Prevention	March 2015 – present
Goossens (2021)[27]	RCT	REMAP-CAP	Severe pneumonia and COVID-19		UK/EU, North America, Oceania, Asia		Drug	Effectiveness	April 2016 – present
Grarup (2015)[31]	RCT	START	HIV	237	North America, South America, UK/EU, Oceania, Africa	4000	Drug	Efficacy	April 2009 – July 2022
Hata (2020)[40]	RCT	PATHWAY	Breast cancer	23	Asia	185	Drug	Efficacy	February 2018 – July 2022
Herrick (2012)[34]	RCT	FDTT	Functional dyspepsia	8	North America	292	Drug	Efficacy	October 2006 – July 2013
Jeon (2016)[46]	RCT	CLEAR III	Intracerebral haemorrhage	73	North America, South America, UK/EU, Asia	500	Drug	Efficacy	September 2009 – January 2015

For peer review only

Kenyon (2011)[21]	RCT	STICH II	Intracerebral haemorrhage	126	North America, Oceania, UK/EU, Asia, Africa	601	Other	Efficacy	January 2007 – August 2015
Kesho Bora Study Group (2011)[35]	RCT	Kesho-Bora	HIV	5	Africa	824	Drug	Efficacy	June 2005 – August 2008
Kolitsopoulos (2013)[47]	RCT	ZODIAC	Schizophrenia	226	North America, South America, UK/EU, Asia	18240	Drug	Effectiveness	February 2002 – April 2007
Larson (2016)[22]	Report	INSIGHT trials							
Lingor (2021)[23]	RCT	ROCK-ALS / ROCK-ALS-US	Amyotrophic lateral sclerosis		North America, UK/EU		Drug	Efficacy	February 2019 – present
Minisman (2012)[5]	RCT	MGTX	Myasthenia gravis	79	Global	126	Other	Effectiveness	June 2006 – December 2015
Murray (2022)[24]	RCT	TICO	COVID-19		North America, UK/EU, Asia, Africa		Drug	Effectiveness	August 2020 – present
Neaton (2010)[9]	Report	INSIGHT trials							
Ravinetto (2013)[36]	RCT	4ABC	Malaria	12	Africa	4112	Drug	Efficacy	July 2007 – December 2009
Reams (2018)[41]	RCT	DOVE	Sickle cell disease	51	North America, South America, UK/EU, Asia, Africa	341	Drug	Efficacy	April 2013 – December 2015
Seal (2006)[28]	RCT		Endophthalmitis	24	UK/EU, Asia	35000	Drug	Efficacy	September 2003 – May 2006
Spencer (2012)[54]	RCT	AWARD-5	Type 2 diabetes	111	North America, UK/EU, Asia	1202	Drug	Safety	August 2008 – July 2012
Sydes (2011)[29]	RCT	STAMPEDE	Prostate cancer		UK/EU		Drug	Efficacy	July 2005 – present
Zimmer (2010)[37]	RCT	BAMSG 3-01	Cryptococcal meningitis	13	North America, Asia	143	Drug	Efficacy	May 2005 – April 2008

For peer review only

Table 2. Design characteristics of included trials (n=35)

**Features**	**n (%)**
**Design**	
Parallel	24 (68.6)
Platform	7 (20.0)
Cluster	1 (2.9)
Factorial	3 (8.6)
**Masking**	
Open label	19 (54.3)
Blinded	16 (45.7)
**Number of sites among closed trials**: median (IQR)	40 (13 – 78)
**Continents of site locations**	
Africa	14 (40.0)
Asia	22 (62.9)
North America	27 (77.1)
Oceania	14 (40.0)
South America	14 (40.0)
UK/Europe	25 (71.4)
**Trial status**	
Open	9 (25.7)
Closed	25 (71.4)
Terminated	1 (2.9)
**Sample size among closed trials**: median (IQR)	1202 (332 – 4056)
**Intervention**	
Drug	26 (74.3)
Device	2 (5.7)
Behavioural	3 (8.6)
Other	4 (11.4)
**Primary outcome**	
Efficacy	14 (40.0)
Effectiveness	12 (34.3)
Safety	2 (5.7)
Prevention	7 (20.0)
**Sponsor**	
Academic	31 (88.6)
Industry	3 (8.6)
Unknown	1 (2.9)
**Main funder**	
Academic	1 (2.9)
Government	28 (80.0)
Industry	5 (14.3)
Charity	1 (2.9)

For peer review only

Table 3. Challenges and proposed solutions

	Challenges	Proposed solutions
Study set-up	Identification of appropriate sponsor and agreements	• Clarify roles and responsibilities between sponsor, co-sponsor and legal representative in advance<br>• Ascertain insurance policy requirements prior to country and site selection<br>• Identify a lead site for each country/continent that is familiar with local regulations to act as a coordinating centre for all local sites
	Budget considerations	• Establish site set-up costs early on (including start-up fees, pharmacy dispensing, lab, ethics, administration, archiving and close out)<br>• Consideration of changes in currency exchange rates<br>• Accurate budget predictions for trials spanning several years<br>• Translation costs
	Regulatory and ethical approvals	• Ensure all sites are familiar with the process required for regulatory approvals
Site set-up	Contracts with collaborating sites	• Begin contract negotiations early<br>• Establish coordinating sites or centres within each country or region to manage negotiations<br>• Allocate time and costs for translation<br>• Coordination with legal representatives to clarify country-specific terminology and interpretation of legal responsibilities and GDPR
	Site monitoring	• Plan adequate financial and human resources for in-person visits<br>• Clear escalation procedures and remedial actions for sites which are underperforming
	Channels of communication	• Regular communication with sites and investigators using multiple channels including emails, monthly teleconferences, outreach calls and annual face-to-face meetings<br>• Develop a hierarchical process for managing site queries to avoid unnecessary emails
	Translation	• Plan adequate financial resources and time for translation of essential documents, contracts and drug packaging
Trial management	Site and intervention selection	• Conduct site feasibility assessments during trial planning phase<br>• Use a systematic process to guide intervention selection and removal particularly for adaptive trials
	Recruitment and retention	• Frequent, often weekly communication with study participants using different channels (e.g. letters, phone calls, emails)<br>• Reimbursement of transport costs<br>• Keeping study visits to a minimum
Data management	Technological challenges including internet bandwidth, institutional firewalls and device availability	• Access to technical expertise and availability of real-time troubleshooting all the time<br>• Use of a software with an option for offline data entry
	Delays in data entry and missing data	• Training and retraining of researchers in data entry and protocol compliance
Intervention management	Difficulty in getting import permits	• Use local commercial or pharmacy suppliers
	Drug wastage	• Distribute smaller numbers but frequently replenish study sites
	Shipment delays and errors	• Establish an electronic inventory that allows real time monitoring of drug stock, biospecimen collection and distribution<br>• Staff training
Adaptive specific	Protocol amendments	• Use an overarching master protocol and consent form and add agent-specific information in appendices to speed up ethical reviews
	Handling drug supply for multiple arms	• Use pharmacies that can serve multiple sites within a close geographical area<br>• Centralised drug distribution system<br>• Choose countries with similar drug labelling requirements
	Data entry and analyses	• Predefine stopping rules for each treatment arm<br>• Predefine frequency of data analysis

For peer review only

References

1 Barrios CH, Werutsky G, Martinez-Mesa J. The global conduct of cancer clinical trials: challenges and opportunities. *Am Soc Clin Oncol Educ Book Am Soc Clin Oncol Annu Meet* 2015;:e132-139. doi:10.14694/EdBook_AM.2015.35.e132

2 World Health Organization. WHO COVID-19 Solidarity Therapeutics Trial. 2021.https://www.who.int/emergencies/diseases/novel-coronavirus-2019/global-research-on-novel-coronavirus-2019-ncov/solidarity-clinical-trial-for-covid-19-treatments (accessed 23 Mar 2023).

3 Lang T, Siribaddana S. Clinical trials have gone global: is this a good thing? *PLoS Med* 2012;**9**:e1001228. doi:10.1371/journal.pmed.1001228

4 Thiers FA, Sinskey AJ, Berndt ER. Trends in the globalization of clinical trials. *Nat Rev Drug Discov* 2008;**7**:13–4. doi:10.1038/nrd2441

5 Minisman G, Bhanushali M, Conwit R, *et al.* Implementing clinical trials on an international platform: challenges and perspectives. *J Neurol Sci* 2012;**313**:1–6. doi:10.1016/j.jns.2011.10.004

6 Jeong S, Sohn M, Kim JH, *et al.* Current globalization of drug interventional clinical trials: characteristics and associated factors, 2011–2013. *Trials* 2017;**18**:288. doi:10.1186/s13063-017-2025-1

7 The Adaptive Platform Trials Coalition. Adaptive platform trials: definition, design, conduct and reporting considerations. *Nat Rev Drug Discov* 2019;**18**:797–807. doi:10.1038/s41573-019-0034-3

8 Pocock S, Calvo G, Marrugat J, *et al.* International differences in treatment effect: do they really exist and why?†. *Eur Heart J* 2013;**34**:1846–52. doi:10.1093/eurheartj/eht071

9 Neaton JD, Babiker A, Bohnhorst M, *et al.* Regulatory impediments jeopardizing the conduct of clinical trials in Europe funded by the National Institutes of Health. *Clin Trials* 2010;**7**:705–18. doi:10.1177/1740774510376547

10 Tang M, Joensuu H, Simes RJ, *et al.* Challenges of international oncology trial collaboration-a call to action. *Br J Cancer* 2019;**121**:515–21. doi:10.1038/s41416-019-0532-4

11 Kurzrock R, Pilat S, Bartolazzi M, *et al.* Project Zero Delay: a process for accelerating the activation of cancer clinical trials. *J Clin Oncol Off J Am Soc Clin Oncol* 2009;**27**:4433–40. doi:10.1200/JCO.2008.21.6093

12 Lai J, Forney L, Brinton DL, *et al.* Drivers of Start-Up Delays in Global Randomized Clinical Trials. *Ther Innov Regul Sci* 2021;**55**:212–27. doi:10.1007/s43441-020-00207-2

13 Chow S-C, Corey R. Benefits, challenges and obstacles of adaptive clinical trial designs. *Orphanet J Rare Dis* 2011;**6**:79. doi:10.1186/1750-1172-6-79

14 Brown LC, Graham J, Fisher D, *et al.* Experiences of running a stratified medicine adaptive platform trial: Challenges and lessons learned from 10 years of the FOCUS4 trial in metastatic colorectal cancer. *Clin Trials* 2022;**19**:146–57. doi:10.1177/17407745211069879

15 Page MJ, McKenzie JE, Bossuyt PM, *et al.* The PRISMA 2020 statement: an updated guideline for reporting systematic reviews. *BMJ* 2021;:n71. doi:10.1136/bmj.n71

16 Gumber L, Pratt A, Bardgett M, *et al.* Operational best practice in international clinical trials: a systematic review protocol. Published Online First: 30 January 2023. doi:10.17605/OSF.IO/YVTJB

18 Babiker AG, Emery S, Fatkenheuer G, *et al.* Considerations in the rationale, design and methods of the Strategic Timing of AntiRetroviral Treatment (START) study. *Clin Trials* 2013;**10**:S5–36. doi:10.1177/1740774512440342

For peer review only

19 Crow RA, Hart KA, McDermott MP, *et al.* A checklist for clinical trials in rare disease: obstacles and anticipatory actions-lessons learned from the FOR-DMD trial. *Trials Electron Resour* 2018;**19**:291. doi:10.1186/s13063-018-2645-0

20 Del Alamo M, Buhrer C, Fisher D, *et al.* Identifying obstacles hindering the conduct of academic-sponsored trials for drug repurposing on rare-diseases: an analysis of six use cases. *Trials Electron Resour* 2022;**23**:783. doi:10.1186/s13063-022-06713-y

21 Kenyon GM, Mendelow AD, Gregson BA, *et al.* Obtaining regulatory approval for multicentre randomised controlled trials: experiences in the STICH II trial. *Br J Neurosurg* 2011;**25**:352–6. doi:10.3109/02688697.2010.551675

22 Larson GS, Carey C, Grarup J, *et al.* Lessons learned: Infrastructure development and financial management for large, publicly funded, international trials. *Clin Trials* 2016;**13**:127–36. doi:10.1177/1740774515625974

23 Lingor P, Koch JC, Statl, *et al.* Challenges and opportunities for Multi-National Investigator-Initiated clinical trials for ALS: European and United States collaborations. *Amyotroph Lateral Scler Front Degener* 2021;**22**:419–25. doi:10.1080/21678421.2021.1879866

24 Murray DD, Babiker AG, Baker JV, *et al.* Design and implementation of an international, multi-arm, multi-stage platform master protocol for trials of novel SARS-CoV-2 antiviral agents: Therapeutics for Inpatients with COVID-19 (TICO/ACTIV-3). *Clin Trials* 2022;**19**:52–61. doi:10.1177/17407745211049829

25 Aban IB, Wolfe GI, Cutter GR, *et al.* The MGTX experience: challenges in planning and executing an international, multicenter clinical trial. *J Neuroimmunol* 2008;**201**:80–4. doi:10.1016/j.jneuroim.2008.05.031

26 Berthon-Jones N, Courtney-Vega K, Donaldson A, *et al.* Assessing site performance in the Altair study, a multinational clinical trial. *Trials Electron Resour* 2015;**16**:138. doi:10.1186/s13063-015-0653-x

27 Goossens H, Derde L, Horby P, *et al.* The European clinical research response to optimise treatment of patients with COVID-19: lessons learned, future perspective, and recommendations. *Lancet Infect Dis* 2022;**22**:e153–8. doi:10.1016/S1473-3099(21)00705-2

28 Seal DV, Barry P, Gettinby G, *et al.* ESCRS study of prophylaxis of postoperative endophthalmitis after cataract surgery. Case for a European multicenter study. *J Cataract Refract Surg* 2006;**32**:396–406. doi:10.1016/j.jcrs.2006.02.014

29 Sydes MR, Parmar MK, Mason MD, *et al.* Flexible trial design in practice - stopping arms for lack-of-benefit and adding research arms mid-trial in STAMPEDE: a multi-arm multi-stage randomized controlled trial. *Trials Electron Resour* 2012;**13**:168. doi:10.1186/1745-6215-13-168

30 Official Journal of the European Union. Regulation (EU) No 536/2014 of the European Parliament and of the Council of 16 April 2014 on clinical trials on medicinal products for human use, and repealing Directive 2001/20/EC Text with EEA relevance. 2022. https://eur-lex.europa.eu/legal-content/EN/TXT/?uri=CELEX%3A02014R0536-20221205 (accessed 4 May 2023).

31 Grarup J, Rappoport C, Engen N, *et al.* Challenges, successes and patterns of enrolment in the INSIGHT Strategic Timing of AntiRetroviral Treatment (START) trial. *HIV Med* 2015;**16**:14–23. doi:10.1111/hiv.12229

32 Aitken LM, Pelter MM, Carlson B, *et al.* Effective strategies for implementing a multicenter international clinical trial. *J Nurs Scholarsh* 2008;**40**:101–8. doi:10.1111/j.1547-5069.2008.00213.x

33 Angus DC, Berry S, Lewis RJ, *et al.* The REMAP-CAP (Randomized Embedded Multifactorial Adaptive Platform for Community-acquired Pneumonia) Study. Rationale and Design. *Ann Am Thorac Soc* 2020;**17**:879–91. doi:10.1513/AnnalsATS.202003-192SD

34 Herrick LM, Locke 3rd GR, Zinsmeister AR, *et al.* Challenges and lessons learned in conducting comparative-effectiveness trials. *Am J Gastroenterol* 2012;**107**:644–9.

For peer review only

35 Kesho Bora Study Group. Safety and effectiveness of antiretroviral drugs during pregnancy, delivery and breastfeeding for prevention of mother-to-child transmission of HIV-1: The Kesho Bora Multicentre Collaborative Study rationale, design, and implementation challenges. *Contemp Clin Trials* 2011;**32**:74–85. doi:10.1016/j.cct.2010.09.008

36 Ravinetto RM, Talisuna A, De Crop M, *et al.* Challenges of non-commercial multicentre North-South collaborative clinical trials. *Trop Med Int Health* 2013;**18**:237–41. doi:10.1111/tmi.12036

37 Zimmer LO, Nolen TL, Pramanpol S, *et al.* International collaboration between US and Thailand on a clinical trial of treatment for HIV-associated cryptococcal meningitis. *Contemp Clin Trials* 2010;**31**:34–43. doi:10.1016/j.cct.2009.11.002

38 Aryal D, Beane A, Dondorp AM, *et al.* Operationalisation of the Randomized Embedded Multifactorial Adaptive Platform for COVID-19 trials in a low and lower-middle income critical care learning health system. *Wellcome Open Res* 2021;**6**:14. doi:10.12688/wellcomeopenres.16486.1

39 Eikelboom J, Rangarajan S, Jolly SS, *et al.* The Anti-Coronavirus Therapies (ACT) Trials: Design, Baseline Characteristics, and Challenges. *CJC Open* 2022;**4**:568–76. doi:10.1016/j.cjco.2022.02.010

40 Hata T, Nakamura K, Yonemori K, *et al.* Regulatory and operational challenges in conducting Asian International Academic Trial for expanding the indications of cancer drugs. *Clin Transl Sci* 2021;**14**:1015–25. doi:10.1111/cts.12965

41 Reams SG, Messana L, Brown PB, *et al.* Clinical trial conduct in special populations and developing regions: An overview of the DOVE study in pediatric patients with sickle cell disease. *Contemp Clin Trials* 2018;**64**:88–94. doi:10.1016/j.cct.2017.11.003

42 Antic NA, Heeley E, Anderson CS, *et al.* The Sleep Apnea cardioVascular Endpoints (SAVE) Trial: Rationale, Ethics, Design, and Progress. *Sleep* 2015;**38**:1247–57. doi:10.5665/sleep.4902

43 Carli V, Wasserman C, Wasserman D, *et al.* The saving and empowering young lives in Europe (SEYLE) randomized controlled trial (RCT): methodological issues and participant characteristics. *BMC Public Health* 2013;**13**:479. doi:10.1186/1471-2458-13-479

44 Dutton R, Hauser C, Boffard K, *et al.* Scientific and logistical challenges in designing the CONTROL trial: recombinant factor VIIa in severe trauma patients with refractory bleeding. *Clin Trials* 2009;**6**:467–79. doi:10.1177/1740774509344102

45 Fogelholm M, Larsen TM, Westerterp-Plantenga M, *et al.* PREVIEW: Prevention of Diabetes through Lifestyle Intervention and Population Studies in Europe and around the World. Design, Methods, and Baseline Participant Description of an Adult Cohort Enrolled into a Three-Year Randomised Clinical Trial. *Nutrients* 2017;**9**:20. doi:10.3390/nu9060632

46 Jeon JE, Mighty J, Lane K, *et al.* Participation of a coordinating center pharmacy in a multicenter international study. *Am J Health Syst Pharm* 2016;**73**:1859–68.

47 Kolitsopoulos FM, Strom BL, Faich G, *et al.* Lessons learned in the conduct of a global, large simple trial of treatments indicated for schizophrenia. *Contemp Clin Trials* 2013;**34**:239–47. doi:10.1016/j.cct.2012.12.001

48 Fulda ES, Fichtenbaum CJ, Kileel EM, *et al.* The importance of methods for site performance evaluation in REPRIEVE, a longitudinal, global, multicenter trial. *Contemp Clin Trials* 2023;**124**. doi:10.1016/j.cct.2022.107035

49 Bryant KE, Yuan Y, Engle M, *et al.* Central monitoring in a randomized, open-label, controlled phase 3 clinical trial for a treatment-shortening regimen for pulmonary tuberculosis. *Contemp Clin Trials* 2021;**104**. doi:10.1016/j.cct.2021.106355

50 Coomarasamy A, Williams H, Truchanowicz E, *et al.* PROMISE: First-trimester progesterone therapy in women with a history of unexplained recurrent miscarriages - A randomised, double-blind, placebo-controlled,

For peer review only

international multicentre trial and economic evaluation. *Health Technol Assess* 2016;**20**:7–91. doi:10.3310/hta20410

51 Denholm JT, Venkatesh B, Davis J, *et al.* ASCOT ADAPT study of COVID-19 therapeutics in hospitalised patients: an international multicentre adaptive platform trial. *Trials Electron Resour* 2022;**23**:1014. doi:10.1186/s13063-022-06929-y

52 Franciscus M, Nucci A, Bradley B, *et al.* Recruitment and retention of participants for an international type 1 diabetes prevention trial: a coordinators' perspective. *Clin Trials* 2014;**11**:150–8. doi:10.1177/1740774513510070

53 Clasen T, Checkley W, Peel JL, *et al.* Design and Rationale of the HAPIN Study: A Multicountry Randomized Controlled Trial to Assess the Effect of Liquefied Petroleum Gas Stove and Continuous Fuel Distribution. *Environ Health Perspect* 2020;**128**:47008. doi:10.1289/EHP6407

54 Spencer K, Colvin K, Braunecker B, *et al.* Operational challenges and solutions with implementation of an adaptive seamless phase 2/3 study. *J Diabetes Sci Technol* 2012;**6**:1296–304.

55 European Medicines Agency. Clinical Trials Regulation. Eur. Med. Agency. 2022.https://www.ema.europa.eu/en/human-regulatory/research-development/clinical-trials/clinical-trials-regulation (accessed 25 Apr 2023).

56 Fox L, Toms C, Kernaghan S, *et al.* Conducting non-commercial international clinical trials: the ICR-CTSU experience. *Trials* 2017;**18**:440, s13063-017-2176–0. doi:10.1186/s13063-017-2176-0

57 International Network for Strategic Initiative in Global HIV Trials. New science in the International Network for Strategic Initiatives in Global HIV Trials (INSIGHT): Why, What and How. 2011. http://insight.ccbr.umn.edu/governance_committees/ssc/WhyWhatWhoToDoResearch.pdf (accessed 9 Mar 2023).

58 Love MN, Fathallh-Shaykh H. Network for Excellence in Neuroscience Clinical Trials: NeuroNEXT. *JAMA Neurol* Published Online First: 5 August 2013. doi:10.1001/jamaneurol.2013.3663

59 Snowdon C, Kernaghan S, Moretti L, *et al.* Operational complexity versus design efficiency: challenges of implementing a phase IIa multiple parallel cohort targeted treatment platform trial in advanced breast cancer. *Trials* 2022;**23**:372. doi:10.1186/s13063-022-06312-x

For peer review only

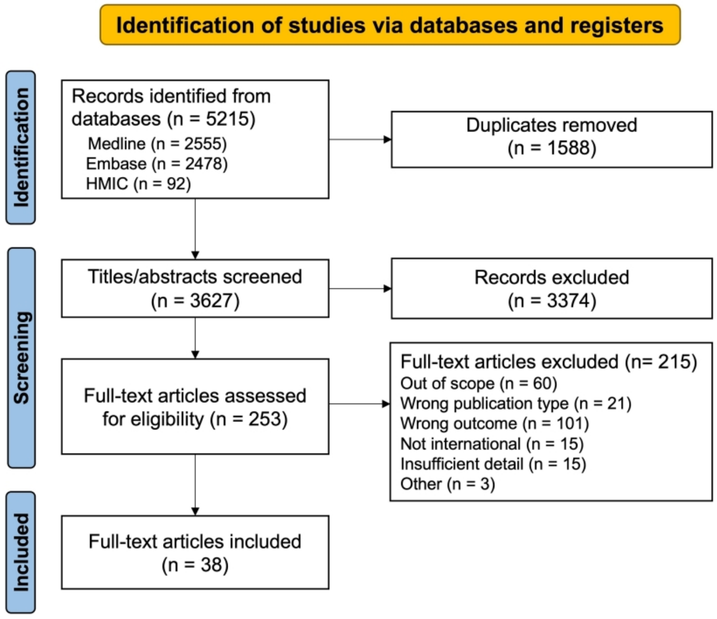

Figure 1. PRISMA Flow Diagram.
*Wrong outcome was defined as studies not discussing operational challenges of conducting international trials.
**Wrong publication type was defined as protocol, conference abstract or systematic review.

254x190mm (300 x 300 DPI)

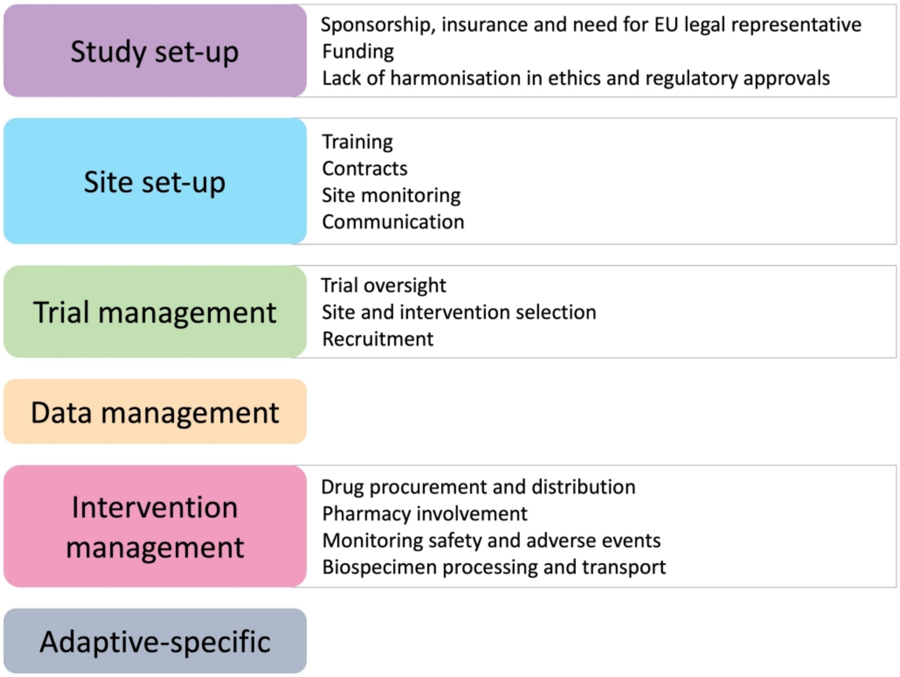

Figure 2. Operational complexities of international trials

254x190mm (300 x 300 DPI)

Appendix 1 – Search Strategies

Database: MEDLINE (Ovid), 31 January 2023

1 adaptive clinical trial/
2 ((adaptive or umbrella or basket or bucket) adj2 (design or trial or trials or study or studies or protocol$)).mp.
3 (trial$ platform$ or platform design or platform trial$ or platform clinical trial$ or platform study or platform studies or platform protocol$).mp.
4 ((adaptive adj6 design$) and (trial or trials or study or studies or protocol$)).mp.
5 (complex innovative adj3 (trial$ or design$ or protocol$)).mp.
6 master protocol$.mp.
7 response adaptive randomi$.mp.
8 (single centre or single site).mp.
9 (or/1-7) not 8 [adaptive design - not single-site]
10 (international adj5 (trial or trials or protocol$ or multicent$ or multi-cent$ or multisite or multi-site or multi-arm or multiarm or multi-stage or multistage)).mp.
11 (international clinical$ trial$ regist$ or international clinical$ trial$ platform$ or international standard randomi$ control$ trial$ number$).mp.
12 international.mp. /freq=2
13 10 not (11 not 12) [gets rid of results with 'international trial' that only refer to the WHO ICTRP]
14 ((multinational or multi-national or intercontinental or inter-continental or pan-europ$) adj8 (trial or trials or protocol$ or multicent$ or multi-cent$ or multisite or multi-site)).mp.
15 ((trial or trials or protocol$) and (((multicent$ or multi-cent$ or multisite or multi-site) and sites) or centres or centers) and countries).mp.
16 ((UK or United Kingdom) and (EU or Europ$) and (trial$ or protocol$)).mp.
17 (europe/ or european alpine region/ or andorra/ or austria/ or balkan peninsula/ or belgium/ or exp europe, eastern/ or exp france/ or exp germany/ or gibraltar/ or greece/ or ireland/ or exp italy/ or liechtenstein/ or luxembourg/ or exp mediterranean region/ or monaco/ or netherlands/ or portugal/ or san marino/ or exp "scandinavian and nordic countries"/ or spain/ or switzerland/ or exp transcaucasia/ or exp ussr/ or vatican city/) and exp United Kingdom/ and (trial$ or protocol$).mp.
18 (exp africa/ or exp americas/ or exp asia/ or exp oceania/) and exp europe/ and (trial$ or protocol$).mp.
19 or/13-18 [main international trial requirement]
20 Methods/
21 Research Design/
22 challenge$.mp.
23 (((trial$ or design) adj5 efficiency) or efficiencies).mp.
24 ((complexit$ or guidance or guidelines or recommendations or considerations or issues or obstacles or barriers) and trial$).ti.
25 ((lesson or lessons or pitfall or pitfalls) and (design$ or plan$)).mp.
26 (trial$ adj5 (manag$ or run or running or conducting)).mp.
27 (design$ or rationale$ or implement$ or methodol$).ti.
28 (trial$ and (framework$ or regulations)).ti.
29 (trials and (Europ$ or EU or multinational or multi-national or intercontinental or inter-continental or international or multi-cent$ or multicent$ or multi-site or multisite)).ti.
30 (trial$ adj1 (approval or authorisation or authorization or regulation)).mp.
31 "organization and administration"/ or capacity building/ or decision making, organizational/ or efficiency/ or efficiency, organizational/ or organizational culture/ or workforce/ or models, organizational/ or organizational innovation/ or change management/ or organizational objectives/ or personnel management/ or leadership/ or personnel selection/ or "personnel staffing and scheduling"/ or staff development/ or planning techniques/ or strategic planning/ or program development/ or total quality management/
32 Multicenter Studies as Topic/
33 exp *Clinical Trials as Topic/ or exp Clinical Trials as Topic/mt, st
34 (trial$ and design$).kf.
35 (((good or best) adj3 practice$) and trial$).ti.
36 (trial$ adj2 (authoris$ or authoriz$)).mp.
37 or/20-36 [general study design/methodology terms]
38 ((trial$ adj5 insur$) or insuring or indemni$).mp.
39 ((add$ or remov$) adj5 (arm or arms)).mp.

For peer review only

40 gdpr.mp.
41 exp *ethics/ or ethic$.ti.
42 ((data adj8 secur$) or (data adj3 (share$ or sharing)) or data management or data governance).mp. or computer security/ or data anonymization/
43 data accuracy/
44 (trial$ adj3 infrastructure).mp.
45 exp *models, statistical/ or (exp models, statistical/ and ((statistical adj3 (model$ or framework$ or plan$)) or estimand$).ti,ab.) or (statistical adj1 design$).mp.
46 software/ or database management systems/ or software design/ or software validation/
47 (reporting guideline$ or outcome reporting).kf. or reporting.ti. or (reporting adj4 (quality or guideline$)).mp.
48 terminology/
49 (recruit$ adj4 (effective$ or model$ or pause$ or pausing)).mp. or recruit$.ti. or *Patient Selection/
50 (decision$ adj2 algorithm$).mp.
51 sponsor$.ti.
52 fund$.ti.
53 resourcing.mp.
54 exp financial management/ or financing, organized/ or taxes/ or (financ$ not incentiv$).ti.
55 economic$.ti.
56 (trial$ adj1 cost$).mp. or (costs or costing).ti.
57 contract research org$.mp.
58 (regulations or (regulat$ adj3 framework$)).mp.
59 Government Regulation/
60 jurisprudence/ or confidentiality/ or personally identifiable information/ or international law/ or legal services/ or liability, legal/ or mandatory reporting/ or legislation, drug/
61 ((legal$ or law or laws or legislat$ or policy or policies or rule or rules) adj5 (UK or United Kingdom or brit$ or EU or Europe$ or framework$ or national or government$)).mp.
62 (clinical trial regulation or "536/2014").mp.
63 ((EU or Europe or European) adj4 representative$).mp.
64 collaborat$.ti.
65 (harmonis$ or harmoniz$).mp.
66 brexit.mp.
67 (safety adj3 (monitor$ or governance)).mp. or Patient Safety/
68 (((intervention or IMP or investigational medicin$ or drug$) adj6 (supply$ or inventory or export$ or import$)) or procur$).mp.
69 (treatment selection or site selection).mp.
70 workload$.mp.
71 Contracts/ or (contract or contracts).mp.
72 ((fund$ or collaborat$ or delegat$ or site or sites or research or sponsor$) adj5 (agreement or agreements)).mp.
73 ((operational or practical or legal or administrative or financial or procedural or ethical or methodolog$ or statistical or recruitment or sponsor$ or logistic$ or design) adj4 (complexit$ or guidance or guidelines or recommendations or considerations or issues or challenges or obstacles or barriers or difficulties or advantages or disadvantages)).mp.
74 exp *Clinical Trials as Topic/es, lj, og, st, sd [Ethics, Legislation & Jurisprudence, Organization & Administration, Supply & Distribution]
75 or/38-74 [any of the various specific challenges]
76 (9 or 19) and 37 and 75
77 (9 or 19) and (*Research Design/ or *Methods/)
78 9 and 19 and (37 or 75)
79 76 or 77 or 78
80 limit 79 to yr="2006 -Current"
81 80 and (exp clinical trial/ or trial$.mp.)
82 limit 81 to ("review" or "scientific integrity review" or "systematic review")
83 81 not (82 and (systematic and review).ti.)
84 83 not (exp animals/ not humans.sh.)
85 limit 84 to english language

For peer review only

Database: EMBASE, 31 January 2023

1	adaptive clinical trial/
2	((adaptive or umbrella or basket or bucket) adj2 (design or trial or trials or study or studies or protocol$)).mp.
3	(trial$ platform$ or platform design or platform trial$ or platform clinical trial$ or platform study or platform studies or platform protocol$).mp.
4	((adaptive adj6 design$) and (trial or trials or study or studies or protocol$)).mp.
5	(complex innovative adj3 (trial$ or design$ or protocol$)).mp.
6	master protocol$.mp.
7	response adaptive randomi$.mp.
8	(single centre or single site).mp.
9	(or/1-7) not 8 [adaptive design - not single-site]
10	(international adj5 (trial or trials or protocol$ or multicent$ or multi-cent$ or multisite or multi-site or multi-arm or multiarm or multi-stage or multistage)).mp.
11	(international clinical$ trial$ regist$ or international clinical$ trial$ platform$ or international standard randomi$ control$ trial$ number$).mp.
12	international.mp. /freq=2
13	10 not (11 not 12) [gets rid of results with 'international trial' that only refer to the WHO ICTRP]
14	((multinational or multi-national or intercontinental or inter-continental or pan-europ$) adj8 (trial or trials or protocol$ or multicent$ or multi-cent$ or multisite or multi-site)).mp.
15	((trial or trials or protocol$) and (((multicent$ or multi-cent$ or multisite or multi-site) and sites) or centres or centers) and countries).mp.
16	((UK or United Kingdom) and (EU or Europ$) and (trial$ or protocol$)).mp.
17	(western europe/ or austria/ or exp belgium/ or benelux/ or exp france/ or germany/ or ireland/ or liechtenstein/ or luxembourg/ or monaco/ or netherlands/ or exp scandinavia/ or switzerland/ or exp Eastern Europe/ or exp Southern Europe/) and exp United Kingdom/ and (trial$ or protocol$).mp.
18	((exp Eastern Europe/ and exp Southern Europe/) or (exp Eastern Europe/ and exp Western Europe/) or (exp Western Europe/ and exp Southern Europe/)) and (trial$ or protocol$).mp.
19	(exp western hemisphere/ or exp africa/ or exp asia/ or exp "australia and new zealand"/) and exp Europe/ and (trial$ or protocol$).mp.
20	or/13-19 [main international trial requirement]
21	methodology/
22	study design/
23	challenge$.mp.
24	(((trial$ or design) adj5 efficiency) or efficiencies).mp.
25	((complexit$ or guidance or guidelines or recommendations or considerations or issues or obstacles or barriers) and trial$).ti.
26	((lesson or lessons or pitfall or pitfalls) and (design$ or plan$)).mp.
27	(trial$ adj5 (manag$ or run or running or conducting)).mp.
28	(design$ or rationale$ or implement$ or methodol$).ti.
29	(trial$ and (framework$ or regulations)).ti.
30	(trials and (Europ$ or EU or multinational or multi-national or intercontinental or inter-continental or international or multi-cent$ or multicent$ or multi-site or multisite)).ti.
31	(trial$ adj1 (approval or authorisation or authorization or regulation)).mp.
32	management/ or joint venture/ or total quality management/ or work schedule/ or workflow/
33	"multicenter study (topic)"/
34	exp *"clinical trial (topic)"/
35	(trial$ and design$).kf.
36	(((good or best) adj3 practice$) and trial$).ti.
37	(trial$ adj2 (authoris$ or authoriz$)).mp.
38	or/21-37 [general study design/methodology terms]
39	((trial$ adj5 insur$) or insuring or indemni$).mp.
40	((add$ or remov$) adj5 (arm or arms)).mp.
41	gdpr.mp.
42	exp *ethics/ or ethic$.ti.
43	((data adj8 secur$) or (data adj3 (share$ or sharing)) or data management or data governance).mp. or data protection/ or anonymization/ or data privacy/ or encryption/
44	data quality/ or data accuracy/ or data availability/ or data completeness/ or data consistency/ or data validity/
45	(trial$ adj3 infrastructure).mp.

For peer review only

46 *statistical model/ or *statistical analysis/ or ((statistical model/ or statistical analysis/) and ((statistical adj3 (model$ or framework$ or plan$)) or estimand$).ti,ab.) or statistical design/ or (statistical adj1 design$).mp.

47 software/ or software design/ or general software/ or exp data analysis software/

48 (reporting guideline$ or outcome reporting).kf. or reporting.ti. or (reporting adj4 (quality or guideline$)).mp.

49 nomenclature/

50 (recruit$ adj4 (effective$ or model$ or pause$ or pausing)).mp. or recruit$.ti. or *patient selection/

51 (decision$ adj2 algorithm$).mp.

52 sponsor$.ti.

53 funding/ or fund$.ti.

54 resourcing.mp.

55 financial management/ or accounting/ or "billing and claims"/ or budget/ or finance/ or corporate finance/ or public finance/ or purchasing/ or exp tax/ or resource management/ or resource allocation/ or (financ$ not incentiv$).ti.

56 economic evaluation/ or economic$.ti.

57 (trial$ adj1 cost$).mp. or (costs or costing).ti.

58 contract research org$.mp.

59 (regulations or (regulat$ adj3 framework$)).mp.

60 *european medicines agency/ or *"medicines and healthcare products regulatory agency"/

61 legal aspect/ or government regulation/ or legal evidence/ or legal liability/ or legal procedure/ or legal service/ or medical liability/ or medicolegal aspect/ or jurisprudence/

62 ((legal$ or law or laws or legislat$ or policy or policies or rule or rules) adj5 (UK or United Kingdom or brit$ or EU or Europe$ or framework$ or national or government$)).mp.

63 (clinical trial regulation or "536/2014").mp.

64 ((EU or Europe or European) adj4 representative$).mp.

65 collaborat$.ti.

66 (harmonis$ or harmoniz$).mp.

67 brexit.mp.

68 (safety adj3 (monitor$ or governance)).mp.

69 (((intervention or IMP or investigational medicin$ or drug$) adj6 (supply$ or inventory or export$ or import$)) or procur$).mp.

70 (treatment selection or site selection).mp.

71 workload$.mp.

72 contract/ or (contract or contracts).mp.

73 ((fund$ or collaborat$ or delegat$ or site or sites or research or sponsor$) adj5 (agreement or agreements)).mp.

74 ((operational or practical or legal or administrative or financial or procedural or ethical or methodolog$ or statistical or recruitment or sponsor$ or logistic$ or design) adj4 (complexit$ or guidance or guidelines or recommendations or considerations or issues or challenges or obstacles or barriers or difficulties or advantages or disadvantages)).mp.

75 or/39-74 [any of the various specific challenges]

76 (9 or 20) and 38 and 75

77 (9 or 20) and (*study design/ or *methodology/)

78 9 and 20 and (38 or 75)

79 76 or 77 or 78

80 limit 79 to conference abstract

81 79 not 80

82 limit 81 to yr="2006 -Current"

83 82 and (trial$.mp. or clinical research/ or exp clinical trial/)

84 limit 83 to ("systematic review" or "review")

85 83 not (84 and (systematic and review).ti.)

86 85 not ((exp animal/ or animal experiment/ or nonhuman/) not (exp human/ or human experiment/))

87 limit 86 to english language

For peer review only

Database: Health Management Information Consortium, 31 January 2023

1	((adaptive or umbrella or basket or bucket) adj2 (design or trial or trials or study or studies or protocol$)).mp.
2	(trial$ platform$ or platform design or platform trial$ or platform clinical trial$ or platform study or platform studies or platform protocol$).mp.
3	((adaptive adj6 design$) and (trial or trials or study or studies or protocol$)).mp.
4	(complex innovative adj3 (trial$ or design$ or protocol$)).mp.
5	master protocol$.mp.
6	response adaptive randomi$.mp.
7	(single centre or single site).mp.
8	(or/1-6) not 7 [adaptive design - not single-site]
9	(international adj5 (trial or trials or protocol$ or multicent$ or multi-cent$ or multisite or multi-site or multi-arm or multiarm or multi-stage or multistage)).mp.
10	(international clinical$ trial$ regist$ or international clinical$ trial$ platform$ or international standard randomi$ control$ trial$ number$).mp.
11	international.mp. /freq=2
12	9 not (10 not 11) [gets rid of results with 'international trial' that only refer to the WHO ICTRP]
13	((multinational or multi-national or intercontinental or inter-continental or pan-europ$) adj8 (trial or trials or protocol$ or multicent$ or multi-cent$ or multisite or multi-site)).mp.
14	((trial or trials or protocol$) and (((multicent$ or multi-cent$ or multisite or multi-site) and sites) or centres or centers) and countries).mp.
15	((UK or United Kingdom) and (EU or Europ$) and (trial$ or protocol$)).mp.
16	(europe/ or albania/ or alps/ or andorra/ or austria/ or balkans/ or baltic countries/ or belarus/ or belgium/ or bosnia herzegovina/ or bulgaria/ or caucasus/ or central europe/ or croatia/ or cyprus/ or czechoslovakia/ or danube river/ or eastern europe/ or france/ or fyr macedonia/ or germany/ or gibraltar/ or greece/ or hungary/ or iberian peninsula/ or italy/ or liechtenstein/ or luxembourg/ or malta/ or mediterranean/ or moldova/ or monaco/ or montenegro/ or netherlands/ or papal states/ or poland/ or "republic of ireland"/ or romania/ or russia/ or san marino/ or scandinavia/ or serbia/ or slovenia/ or soviet union/ or switzerland/ or turkey/ or ukraine/ or yugoslavia/) and united kingdom/ and (trial$ or protocol$).mp.
17	(exp africa/ or exp asia/ or exp middle east/ or exp americas/ or exp oceania/) and exp europe/ and (trial$ or protocol$).mp.
18	or/12-17 [main international trial requirement]
19	methodology/
20	exp research strategies/
21	challenge$.mp.
22	(((trial$ or design) adj5 efficiency) or efficiencies).mp.
23	((complexit$ or guidance or guidelines or recommendations or considerations or issues or obstacles or barriers) and trial$).ti.
24	((lesson or lessons or pitfall or pitfalls) and (design$ or plan$)).mp.
25	(trial$ adj4 (manag$ or run or running or conducting)).mp.
26	(design$ or rationale$ or implement$ or methodol$).ti.
27	management/ or business management/ or corporate management/ or development management/ or joint management/ or office management/ or operational management/ or process management/ or programme management/ or project management/ or quality management/ or research management/ or strategic management/ or team management/ or work organisation/ or administration/ or leadership/ or management communication/ or management operations/ or management planning/ or management practice/ or management process/ or management techniques/
28	contract management/ or facilities management/ or financial management/ or human resources management/ or information management/ or knowledge management/ or materials & supplies management/ or physical distribution management/ or resource management/ or risk management/
29	(((good or best) adj3 practice$) and trial$).ti.
30	(trial$ adj2 (authoris$ or authoriz$)).mp.
31	or/19-30
32	((trial$ adj5 insur$) or insuring or indemni$).mp.
33	((add$ or remov$) adj5 (arm or arms)).mp.
34	gdpr.mp.
35	exp ethics/ or ethic$.ti.
36	ethics committees/

For peer review only

37	((data adj8 secur$) or (data adj3 (share$ or sharing)) or data management or data governance).mp.
38	(trial$ adj3 infrastructure).mp.
39	statistical model/ or statistical analysis/ or statistical design/ or (statistical adj1 design$).mp.
40	software.mp.
41	reporting.ti. or (reporting adj4 (quality or guideline$)).mp.
42	(recruit$ adj4 (effective$ or model$ or pause$ or pausing)).mp. or recruit$.ti.
43	(decision$ adj2 algorithm$).mp.
44	sponsor$.ti.
45	fund$.ti.
46	exp financing/ or (financ$ not incentiv$).ti.
47	resourcing.mp.
48	economic evaluation/ or economic.ti.
49	(trial$ adj1 cost$).mp. or (costs or costing).ti.
50	contract research org$.mp.
51	(regulations or (regulat$ adj3 framework$)).mp. or drug regulations/ or regulations/
52	((legal$ or law or laws or legislat$ or policy or policies or rule or rules) adj5 (UK or United Kingdom or brit$ or EU or Europe$ or framework$ or national or government$)).mp.
53	(clinical trial regulation or "536/2014").mp.
54	((EU or Europe or European) adj4 representative$).mp.
55	collaborat$.ti.
56	(harmonis$ or harmoniz$).mp.
57	brexit.mp.
58	(safety adj3 (monitor$ or governance)).mp. or drug safety/
59	(((intervention or IMP or investigational medicin$ or drug$) adj6 (supply$ or inventory or export$ or import$)) or procur$).mp.
60	(treatment selection or site selection).mp.
61	workload$.mp.
62	exp contracts/ or (contract or contracts).mp.
63	((fund$ or collaborat$ or delegat$ or site or sites or research or sponsor$) adj5 (agreement or agreements)).mp.
64	((operational or practical or legal or administrative or financial or procedural or ethical or methodolog$ or statistical or recruitment or sponsor$ or logistic$ or design) adj4 (complexit$ or guidance or guidelines or recommendations or considerations or issues or challenges or obstacles or barriers or difficulties or advantages or disadvantages)).mp.
65	or/32-64
66	(8 or 18) and (31 or 65)
67	limit 66 to yr="2006 -Current"
68	limit 67 to english
69	68 not systematic review.ti.

For peer review only

Appendix 2 – Operational challenges reported in included studies

Table 1. Operational complexities in conducting international trials

Major barriers	Source
**Study set-up**	
Sponsorship, insurance and need for EU legal representative	Aban (2008); Babiker (2013); Berthon-Jones (2015); Crow (2018); del Álamo (2022); Goossens (2021); Kenyon (2011); Lingor (2021); Minisman (2012); Murray (2022); Neaton (2010); Seal (2006); Sydes (2011)
Funding	Aban (2008); Aitken (2008); Angus (2020); Babiker (2013); Crow (2018); del Álamo (2022); Goossens (2021); Herrick (2012); Kenyon (2011); Kesho Bora Study Group (2011); Larson (2016); Lingor (2021); Ravinetto (2013); Zimmer (2010)
Lack of harmonisation in ethics and regulatory approvals	Aban (2008); Aitken (2008); Aryal (2021); Babiker (2013); Berthon-Jones (2015); del Álamo (2022); Eikelboom (2022); Goossens (2021); Grarup (2015); Hata (2020); Kenyon (2011); Minisman (2012); Murray (2022); Neaton (2010); Ravinetto (2013); Reams (2018); Zimmer (2010)
**Site set-up**	
Training	Aitken (2008); Antic (2015); Aryal (2021); Carli (2013); Crow (2018); Dutton (2009); Fogelholm (2017); Jeon (2016); Kesho Bora Study Group (2011); Kolitsopoulos (2013); Minisman (2012)
Contracts	Babiker (2013); Crow (2018); del Álamo (2022); Goossens (2021); Grarup (2015); Larson (2016); Lingor (2021); Minisman (2012)
Site monitoring	Angus (2020); Aryal (2021); Bryant (2021); Carli (2013); Coomarasamy (2016); Denholm (2022); Dutton (2009); Franciscus (2014); Fulda (2023); Larson (2016); Ravinetto (2013); Seal (2006); Sydes (2011); Zimmer (2010)
Communication	Aitken (2008); Angus (2020); Antic (2015); Crow (2018); Denholm (2022); Fulda (2023); Herrick (2012); Minisman (2012)
Translation of materials	Aban (2008); Babiker (2013); Berthon-Jones (2015); Carli (2013); Crow (2018); Franciscus (2014); Lingor (2021); Zimmer (2010)
**Trial management**	
Trial oversight	Antic (2015); Babiker (2013); Coomarasamy (2016); Dutton (2009); Franciscus (2014); Herrick (2012); Larson (2016); Murray (2022); Spencer (2012)
Site and intervention selection	Angus (2020); Antic (2015); Coomarasamy (2016); Denholm (2022); Dutton (2009); Eikelboom (2022); Fulda (2023); Herrick (2012); Kesho Bora Study Group (2011); Kolitsopoulos (2013); Minisman (2012); Murray (2022); Reams (2018); Zimmer (2010)
Recruitment	Aitken (2008); Antic (2015); Berthon-Jones (2015); Eikelboom (2022); Franciscus (2014); Herrick (2012); Kesho Bora Study Group (2011); Kolitsopoulos (2013); Reams (2018)
**Data management**	Aitken (2008); Aryal (2021); Berthon-Jones (2015); Carli (2013); Coomarasamy (2016); Ravinetto (2013); Seal (2006)
**Intervention management**	
Drug procurement and distribution	Babiker (2013); Bryant (2021); Coomarasamy (2016); Crow (2018); del Álamo (2022); Goossens (2021); Grarup (2015); Hata (2020); Herrick (2012); Jeon (2016); Lingor (2021); Minisman (2012); Murray (2022); Ravinetto (2013); Reams (2018); Seal (2006); Spencer (2012); Zimmer (2010)
Pharmacy involvement	Aban (2008); Bryant (2021); Coomarasamy (2016); Herrick (2012); Jeon (2016); Kolitsopoulos (2013); Minisman (2012)
Monitoring safety and adverse events	Antic (2015); Bryant (2021); Coomarasamy (2016); Hata (2020); Herrick (2012); Minisman (2012); Murray (2022); Zimmer (2010)
Biospecimen processing and transport	Berthon-Jones (2015); Bryant (2021); Denholm (2022); Fogelholm (2017); Franciscus (2014); Fulda (2023); Hata (2020); Lingor (2021)
**Adaptive-specific**	Angus (2020); Aryal (2021); Denholm (2022); Murray (2022); Spencer (2012)

For peer review only - http://bmjopen.bmj.com/site/about/guidelines.xhtml

Appendix 3 – PRISMA checklist

Section and Topic	Item #	Checklist item	Location where item is reported
**TITLE**			
Title	1	Identify the report as a systematic review.	Page 1
**ABSTRACT**			
Abstract	2	See the PRISMA 2020 for Abstracts checklist.	Page 2
**INTRODUCTION**			
Rationale	3	Describe the rationale for the review in the context of existing knowledge.	Page 4-5
Objectives	4	Provide an explicit statement of the objective(s) or question(s) the review addresses.	Page 4-5
**METHODS**			
Eligibility criteria	5	Specify the inclusion and exclusion criteria for the review and how studies were grouped for the syntheses.	Page 5-6
Information sources	6	Specify all databases, registers, websites, organisations, reference lists and other sources searched or consulted to identify studies. Specify the date when each source was last searched or consulted.	Page 5
Search strategy	7	Present the full search strategies for all databases, registers and websites, including any filters and limits used.	Page 5
Selection process	8	Specify the methods used to decide whether a study met the inclusion criteria of the review, including how many reviewers screened each record and each report retrieved, whether they worked independently, and if applicable, details of automation tools used in the process.	Page 5-6
Data collection process	9	Specify the methods used to collect data from reports, including how many reviewers collected data from each report, whether they worked independently, any processes for obtaining or confirming data from study investigators, and if applicable, details of automation tools used in the process.	Page 5-6
Data items	10a	List and define all outcomes for which data were sought. Specify whether all results that were compatible with each outcome domain in each study were sought (e.g. for all measures, time points, analyses), and if not, the methods used to decide which results to collect.	Page 6
	10b	List and define all other variables for which data were sought (e.g. participant and intervention characteristics, funding sources). Describe any assumptions made about any missing or unclear information.	Page 6
Study risk of bias assessment	11	Specify the methods used to assess risk of bias in the included studies, including details of the tool(s) used, how many reviewers assessed each study and whether they worked independently, and if applicable, details of automation tools used in the process.	NA
Effect measures	12	Specify for each outcome the effect measure(s) (e.g. risk ratio, mean difference) used in the synthesis or presentation of results.	Page 6-7
Synthesis methods	13a	Describe the processes used to decide which studies were eligible for each synthesis (e.g. tabulating the study intervention characteristics and comparing against the planned groups for each synthesis (item #5)).	Page 5-7
	13b	Describe any methods required to prepare the data for presentation or synthesis, such as handling of missing summary statistics, or data conversions.	NA
	13c	Describe any methods used to tabulate or visually display results of individual studies and syntheses.	NA
	13d	Describe any methods used to synthesize results and provide a rationale for the choice(s). If meta-analysis was performed, describe the model(s), method(s) to identify the presence and extent of statistical heterogeneity, and software package(s) used.	Page 6-7
	13e	Describe any methods used to explore possible causes of heterogeneity among study results (e.g. subgroup analysis, meta-regression).	NA
	13f	Describe any sensitivity analyses conducted to assess robustness of the synthesized results.	NA
Reporting bias assessment	14	Describe any methods used to assess risk of bias due to missing results in a synthesis (arising from reporting biases).	NA
Certainty assessment	15	Describe any methods used to assess certainty (or confidence) in the body of evidence for an outcome.	NA
**RESULTS**			
Study selection	16a	Describe the results of the search and selection process, from the number of records identified in the search to the number of studies included in the review, ideally using a flow diagram.	Page 7
	16b	Cite studies that might appear to meet the inclusion criteria, but which were excluded, and explain why they were excluded.	Page 7 / Figure 1
Study characteristics	17	Cite each included study and present its characteristics.	Page 7 / Table 1
Risk of bias in studies	18	Present assessments of risk of bias for each included study.	NA
Results of individual studies	19	For all outcomes, present, for each study: (a) summary statistics for each group (where appropriate) and (b) an effect estimate and its precision (e.g. confidence/credible interval), ideally using structured tables or plots.	Table 2, Page 7-16
Results of syntheses	20a	For each synthesis, briefly summarise the characteristics and risk of bias among contributing studies.	NA
	20b	Present results of all statistical syntheses conducted. If meta-analysis was done, present for each the	NA

For peer review only - http://bmjopen.bmj.com/site/about/guidelines.xhtml

Section and Topic	Item #	Checklist item	Location where item is reported
		summary estimate and its precision (e.g. confidence/credible interval) and measures of statistical heterogeneity. If comparing groups, describe the direction of the effect.	
	20c	Present results of all investigations of possible causes of heterogeneity among study results.	NA
	20d	Present results of all sensitivity analyses conducted to assess the robustness of the synthesized results.	NA
Reporting biases	21	Present assessments of risk of bias due to missing results (arising from reporting biases) for each synthesis assessed.	NA
Certainty of evidence	22	Present assessments of certainty (or confidence) in the body of evidence for each outcome assessed.	NA
**DISCUSSION**			
Discussion	23a	Provide a general interpretation of the results in the context of other evidence.	Page 16-18
	23b	Discuss any limitations of the evidence included in the review.	Page 17
	23c	Discuss any limitations of the review processes used.	Page 17
	23d	Discuss implications of the results for practice, policy, and future research.	Page 16-18
**OTHER INFORMATION**			
Registration and protocol	24a	Provide registration information for the review, including register name and registration number, or state that the review was not registered.	Page 5
	24b	Indicate where the review protocol can be accessed, or state that a protocol was not prepared.	NA
	24c	Describe and explain any amendments to information provided at registration or in the protocol.	NA
Support	25	Describe sources of financial or non-financial support for the review, and the role of the funders or sponsors in the review.	Page 3
Competing interests	26	Declare any competing interests of review authors.	Page 3
Availability of data, code and other materials	27	Report which of the following are publicly available and where they can be found: template data collection forms; data extracted from included studies; data used for all analyses; analytic code; any other materials used in the review.	

For peer review only

Appendix 4 – Protocol

Operational best practice in international clinical trials: a systematic review protocol

Support and registration:
This systematic review has been funded by the NIHR (Application Accelerator Award reference number: NIHR153955). The funder will have no further role in any aspect of the review. This protocol has been registered with the Open Science Framework (registration DOI: https://doi.org/10.17605/OSF.IO/YVTJB) (1).

Aim:
The aim of this systematic review is to identify models of best practice and lessons learned from the set up and delivery of international clinical trials, specifically in regard to management of cross-border regulatory and logistical requirements.

Rationale:
Research practices have transformed over the last decade, evolving from large single centred trials to more global trials that promote international collaboration. International trials offer numerous advantages over single-centre including reduced operational costs particularly in developing countries, faster recruitment and expand the horizon for the availability of treatments. Despite their benefits, the process of expanding to multiple sites raises logistical challenges due to the diversity in laws, ethics, guidelines and regulations between countries.

Search strategy:
The search strategy will be designed in collaboration with an experienced information specialist and will be peer-reviewed by another information specialist using the PRESS checklist. Thesaurus headings and keywords have been used as appropriate, and the search will be translated to other sources accordingly. The search strategy will cover four areas: "international trials", "adaptive design trials", "study design", and "specific challenges" and requirements of designing and running trials. Multiple approaches to combining these aspects will be used to achieve a practical quantity of specific, relevant results, while also mitigating risks of missing relevant material. A wide range of subject headings and search terms in appropriate fields will be used for all concepts in the search, including proximity searching to cover the many variations on phrases applicable to these topics. We will also apply a date filter to include studies published after 2005 as international trials have become prominent only in the last 15-20 years. We will search Medline, Embase and Health Management Information Consortium (HMIC) for appropriate studies.

Participants or population:
As the focus is on the implementation and infrastructure of the trial, any clinical population and any condition will be considered of interest.

Intervention:
Clinical trials on any drug, device or therapeutic intervention will be considered if they are multi-centre and trial site locations are in more than one country.

Comparator:
Trials will be eligible regardless of comparator.

Study designs to be included:
Any multi-centre randomised trial design with sites in at least two different countries will be included.

Eligibility criteria:
The following types of studies will be included:

1. Any study reporting operational challenges to conducting international trials (involving two or more countries)
2. Available in English
3. Published after 2005

Conference abstracts, systematic reviews and protocols will be excluded.

For peer review only

Main outcome:
Any outcomes related to the best practice models and lessons learned in aspects of set up and delivery of international trials.

We will specifically look at:

- Sponsorship and funding: balancing UK, EU and other international trial regulations, policies and procedures in sponsorship of the overall trial
- Trial management structure: legal, ethical and regulatory matters, safety and monitoring standards, processes for adding and/or removing trial arms, GDPR and clinical data management system requirements
- Intervention management: pharmacy involvement, procurement, distribution, delivery of interventions
- Contracting: agreements of collaboration, sites, delegation, sample transfer logistics arrangements and data sharing.

Data management:
Zotero will be used to manage the studies throughout the review. Rayyan will be used to screen records.

Strategy for data synthesis:
Two reviewers will independently screen the titles and abstracts of the studies retrieved by the search. For studies deemed eligible, or studies where it is impossible to decide eligibility from the abstract, the full text will be retrieved, and two reviewers will independently assess for inclusion. Any disagreements will be resolved through discussion or if necessary, by reference to a third reviewer.

Data extraction will be undertaken by one reviewer and checked by a second, with discrepancies resolved by consultation with a third. Where studies are reported in multiple publications, we will extract relevant data from all publications but consider as one study. Where data is missing or unclear, we will contact authors to request details or clarification. The following data will be extracted from included studies:

- Citation information
- Study design variables: sample size, phase
- Study population and objective variables: age, condition, experimental interventions, primary outcome
- Regulatory information: status, location, availability of results, start and completion date
- Outcome data: Sponsorship information; funding information; trial management process information; intervention/pharmacy process information; detail on the contracting processes

In the first instance, we will present a summary of study characteristics and outcome data in a series of structured tables to give a clear picture of the available evidence.

Language:
Only studies published in English will be included.

References

1. Gumber L, Pratt A, Bardgett M, Inskip A, Still M, Phillipson J, et al. Operational best practice in international clinical trials: a systematic review protocol 2023. osf.io/yvtjb.

For peer review only

BMJ Open

Operational complexities in international clinical trials: a systematic review of challenges and proposed solutions

Journal:	*BMJ Open*
Manuscript ID	bmjopen-2023-077132.R1
Article Type:	Original research
Date Submitted by the Author:	17-Jan-2024

Complete List of Authors:	Gumber, Leher; Newcastle University, Translational and Clinical Research Institute; Northumbria Healthcare NHS Foundation Trust,<br>Agbeleye, Opeyemi; Newcastle University, Population Health Sciences Institute<br>Inskip, Alex; Newcastle University, Population Health Sciences Institute<br>Fairbairn, Ross; Newcastle University, Population Health Sciences Institute<br>Still, Madeleine; Newcastle University<br>Ouma, Luke; Newcastle University, Population Health Sciences Institute<br>Lozano-Kuehne, Jingky; Newcastle University, Population Health Sciences Institute<br>Bardgett, Michelle; Newcastle University<br>Isaacs, John; Newcastle University, Translational and Experimental Medicine Institute; Newcastle Upon Tyne Hospitals NHS Foundation Trust, Musculoskeletal Unit<br>Wason, James; Newcastle University, Population Health Sciences Institute; University of Cambridge, MRC Biostatistics Unit<br>Craig, Dawn; Newcastle University, Health Economics Group, Institute of Health and Society<br>Pratt, Arthur; Newcastle University; Newcastle Upon Tyne Hospitals NHS Trust

**Primary Subject Heading**:	Research methods
Secondary Subject Heading:	Research methods, Global health
Keywords:	Clinical Trial, Randomized Controlled Trial, Systematic Review

SCHOLARONE™
Manuscripts

I, the Submitting Author has the right to grant and does grant on behalf of all authors of the Work (as defined in the below author licence), an exclusive licence and/or a non-exclusive licence for contributions from authors who are: i) UK Crown employees; ii) where BMJ has agreed a CC-BY licence shall apply, and/or iii) in accordance with the terms applicable for US Federal Government officers or employees acting as part of their official duties; on a worldwide, perpetual, irrevocable, royalty-free basis to BMJ Publishing Group Ltd ("BMJ") its licensees and where the relevant Journal is co-owned by BMJ to the co-owners of the Journal, to publish the Work in this journal and any other BMJ products and to exploit all rights, as set out in our licence.

The Submitting Author accepts and understands that any supply made under these terms is made by BMJ to the Submitting Author unless you are acting as an employee on behalf of your employer or a postgraduate student of an affiliated institution which is paying any applicable article publishing charge ("APC") for Open Access articles. Where the Submitting Author wishes to make the Work available on an Open Access basis (and intends to pay the relevant APC), the terms of reuse of such Open Access shall be governed by a Creative Commons licence – details of these licences and which Creative Commons licence will apply to this Work are set out in our licence referred to above.

Other than as permitted in any relevant BMJ Author's Self Archiving Policies, I confirm this Work has not been accepted for publication elsewhere, is not being considered for publication elsewhere and does not duplicate material already published. I confirm all authors consent to publication of this Work and authorise the granting of this licence.

For peer review only

Operational complexities in international clinical trials: a systematic review of challenges and proposed solutions

Leher Gumber[1,2], Opeyemi Agbeleye[3], Alex Inskip[3], Ross Fairbairn[3], Madeleine Still[3], Luke Ouma[4], Jingky Lozano-Kuehne[4], Michelle Bardgett[5], John D Isaacs[1,6], James M. S. Wason[4], Dawn Craig[3*] and Arthur G Pratt[1,6*].

[1]Translational and Clinical Research Institute, Newcastle University, UK.
[2]Northumbria Healthcare NHS Foundation Trust, UK.
[3]NIHR Innovation Observatory, Population Health Sciences Institute, Newcastle University, UK.
[4]Population Health Sciences Institute, Newcastle University, UK
[5]Newcastle Clinical Trial Unit, Newcastle University, UK.
[6]Musculoskeletal Unit, Newcastle upon Tyne Hospitals NHS Trust, UK.

*Denotes equal contribution

Corresponding author:
Dr Arthur G Pratt
Senior Clinical Lecturer and Consultant Rheumatologist
Translational and Clinical Research Institute
Newcastle University
Newcastle upon Tyne
NE1 7RU, UK
Email: arthur.pratt@newcastle.ac.uk

For peer review only

Abstract

Objective: International trials can be challenging to operationalise due to incompatibilities between country-specific policies and infrastructures. The aim of this systematic review was to identify the operational complexities of conducting international trials and identify potential solutions for overcoming them.

Design: Systematic review.

Data sources: Medline, Embase and Health Management Information Consortium were searched from 2006 to 30th January 2023.

Eligibility criteria: All studies reporting operational challenges (e.g. site selection, trial management, intervention management, data management) of conducting international trials were included.

Data extraction and synthesis: Search results were independently screened by at least two reviewers and data were extracted into a proforma.

Results: 38 studies (35 RCTs, 2 reports and 1 qualitative study) fulfilled the inclusion criteria. The median sample size was 1202 (IQR 332 – 4056) and median number of sites was 40 (IQR 13 – 78). 88.6% of studies had an academic sponsor and 80% were funded through government sources. Operational complexities were particularly reported during trial set-up due to lack of harmonisation in regulatory approvals and in relation to sponsorship structure, with associated budgetary impacts. Additional challenges included site selection, staff training, lengthy contract negotiations, site monitoring, communication, trial oversight, recruitment, data management, drug procurement and distribution, pharmacy involvement and biospecimen processing and transport.

Conclusions: International collaborative trials are valuable in cases where recruitment may be difficult, diversifying participation and applicability. However, multiple operational and regulatory challenges are encountered when implementing a trial in multiple countries. Careful planning and communication between trials units and investigators, with an emphasis on establishing adequately resourced cross-border sponsorship structures and regulatory approvals, may help to overcome these barriers and realise the benefits of the approach.

Open Science Framework registration number: osf-registrations-yvtjb-v1.

Strengths and limitations:

- A robust search and screening strategy was utilised to maximise the identification of relevant studies.
- All abstracts were screened by at least two reviewers.
- The review focused on international trials so the findings may not be generalisable to multi-centre studies conducted within the same country.
- The possibility that some specific challenges encountered might be omitted cannot be entirely excluded, nor can an element of subjectivity when summarising potential solutions to those identified.

Word count: 4263

Funding: This study was funded by National Institute of Health and Care Research (NIHR153955). Infrastructural support was provided by the National Institute of Health and Care Research (NIHR) Newcastle Biomedical Research Centre. JMSW is funded by a NIHR Research Professorship (NIHR301614). LG is funded by a NIHR Academic Clinical Fellowship. The views expressed are those of the author(s) and not necessarily those of the NIHR or the Department of Health and Social Care.

For peer review only

Competing interests statement: None declared.

Contribution of authors: LG was involved in conception of the study, screening, data extraction, analysis and writing up the manuscript. OA was involved in screening and writing up the manuscript. AI was involved in developing search strategy and revising it critically for important intellectual content. RF, MS were involved in screening and revising it critically for important intellectual content. MB was involved in developing search strategy and revising it critically for important intellectual content. LO, JLK, JDI and JW reviewed the manuscript for important intellectual content. Together with DC, AGP conceived and oversaw the project, revising the manuscript for important intellectual content.

Acknowledgements: We would like to thank Chris Price, Andrew Johnston, Julia Philipson and Ruth Wood for providing insights into outcome measures.

For peer review only

Introduction

The development and deployment of international clinical trials that enrol participants from more than one nation or jurisdiction, continues to increase [1,2]. Motivated by advancements in technology, globalisation and insufficient accrual rates using traditional approaches[3], recent examples increasingly adopt master protocols that allow treatment arms to be added and dropped adaptively over time – so-called "platform" trials – and other innovative designs [4]. International trials offer numerous advantages over single-nation approaches by increasing access to potentially eligible participants, enabling faster recruitment and/or larger sample sizes whilst encouraging representation of diverse ethnic, biological and socio-cultural groups[3,5–7]. They promote best practice globally and expand horizons for treatment availability in countries that may not otherwise have access to particular interventions[7]. The reduced operational cost of running a trial in developing compared to developed countries may be an additional consideration [8]. They furthermore foster collaboration and development of closer relationships among academics globally[3,5,7,9,10].

The conduct of international trials nonetheless presents unique challenges. Their success requires adherence to local and international laws, regulations and ethical requirements, availability of Good Clinical Practice (GCP)-trained researchers, adequate infrastructure at clinical sites, and close monitoring and oversight across multiple jurisdictions[7,11,12]. Incompatibilities between country-specific policies and challenges in the trial setup were previously identified as factors that extend study timelines and inflating costs[13]. Other contributing factors including site selection, insurance, logistics, regulatory requirements and sponsorship have been highlighted in a narrative review [14]. Platform trials present further complexity, potentially involving adaptations that incur ethical and/or regulatory review, sample size re-estimations, site capacity and data management challenges[15,16]. In recognition of these issues, we conducted a systematic review that aimed to identify: (1) the operational challenges of conducting international clinical trials and (2) potential practical solutions for overcoming them. Our overarching objective was to deliver a reference of practical value to prospective trialists planning to set up international trials in the modern era, with the potential to inform best practice guidelines.

Methods

For peer review only - http://bmjopen.bmj.com/site/about/guidelines.xhtml

This study was reported in accordance with the Preferred Reporting Items for Systematic Reviews and Meta-analyses (PRISMA) guidelines[17]. The study was registered with the Open Science Framework (Identifier: osf-registrations-yvtjb-v1)[18] and funded by National Institute of Health and Care Research (NIHR153955).

Search strategy and study selection

A search strategy was developed in collaboration with an information specialist (AI) using a combination of key words and MeSH terms. Medline and Embase were the primary bibliographic database sources, but a variation of the search was also run on the Health Management Information Consortium (HMIC) database. The search covered four broad concepts: (i) international trials, (ii) adaptive trials (in recognition that such trials frequently adopt international enrolment strategies not explicit in titles/abstracts), (iii) specific challenges to conducting trials and (iv) a focus on study design methods. These concepts were utilised in several different combinations to achieve a practicable quantity of relevant results, whilst mitigating the risk of omitting relevant material. Studies conducted prior to 2006 were considered less likely to reflect current legislation and hence a limit was applied to exclude them in the initial search strategy, as were systematic reviews and studies not available in English. A full description of the search strategy is provided in the supplement.

After de-duplication of records, titles and abstracts were rigorously double screened by at least two reviewers. The following studies were excluded: protocols, abstracts, multi-centre trials in a single country, studies published before 2006, studies not in English, systematic reviews and studies which did not report operational challenges. Operational challenges broadly encompassed issues related to approvals, opening sites, recruitment, and data management, and are further detailed in the supplement.

For studies deemed eligible, or where it was deemed impossible to decide eligibility from the abstract, the full text was retrieved. Full-text articles were screened for inclusion by one reviewer and 20% of the full-text articles underwent screening by a second reviewer. In case of discrepancy, the decision was taken by consensus of all the reviewers. Rayyan (Qatar Computing Research Institute), a systematic review web-based application, was used for screening, with record management facilitated throughout using the Zotero reference management tool.

Data extraction

For peer review only

Data were extracted by one reviewer using a pre-designed proforma (supplementary Table S1) and extractions were checked by a second reviewer. Aside from general trial characteristics, data extraction was under the broad headings: sponsorship; funding; regulatory considerations; trial management; intervention; biospecimens; agreements. Due to the nature of the review, a formal quality assessment was not performed.

Definition of variables and data analysis

The sponsor was determined as either academic (universities, hospitals or the government), or industry (pharmaceutical or device companies). The funder was defined as academic (university or hospital), government, industry or charity; where studies were co-funded by more than one source, the predominant funder was reported. Location of studies was categorised by continent into: UK/Europe (EU), Asia, Africa, North America, South America or Oceania. Primary outcome measures were considered as efficacy, effectiveness, prevention or safety. Quantitative data were described using numbers, percentages, median and interquartile range. Qualitative data were summarised into overarching themes which described the main challenges of conducting international trials. In relation to specific challenges identified during data extractions, solutions given by the authors were also recorded where available in each case and synthesised for reporting purposes.

Patient and public involvement

No patients were involved.

Results

The search identified 5215 records, of which 1588 were duplicates and removed. After title and abstract screening of the remaining 3627 articles, a further 3374 were excluded. Full text screening was conducted on 253 articles from which 38 were included (Figure 1). Characteristics of included studies are summarised in Table 1 and further details are available in supplementary Table S2.

Study characteristics

For peer review only

Of the 38 included studies, 35 (92.1%) were RCTs, 2 (5.3%) were reports and 1 (2.6%) was a qualitative study. Of the 35 RCTs, 24 (68.6%) had a parallel design, 7 (20.0%) were adaptive, 3 (8.6%) were factorial and 1 (2.9%) was cluster. Over half of the RCTs were open-label. Most RCTs evaluated a drug intervention (74.3%) and measured efficacy (40.0%) as their primary outcome. The median number of sites and sample size among closed trials was 40 (IQR 13 – 78) and 1202 (IQR 332 – 4056) respectively. The majority of studies (n=31, 88.6%) were sponsored by an academic institution and the most common funding source was government (80.0%). The continents most commonly represented by sites included in studies were North America (n=27, 77.1%), and UK/EU (n=25, 71.4%). Design characteristics of the included studies are summarised in Table 2. Operational complexities of conducting international trials were reported broadly at six stages of the trial process including study set-up, site set-up, trial management, data management, intervention management and adaptive specific features (Figure 2; supplementary Table S3). We structure our findings accordingly herein, summarising recurring themes and potential approaches arising to address them in Table 3.

Study set-up

Sponsorship, insurance and need for EU legal representative

Thirteen studies described variations in sponsorship and insurance requirements between countries which created delays[11,19–31]. This was particularly notable for UK/EU sites. The EU Clinical Trials Directive which governs the conduct of clinical trials in EU, requires the presence of an EU legal representative for trials sponsored by a non-EU institution[32]. After identification of an EU legal representative, repeated negotiations were necessary to clarify roles and responsibilities between the sponsor and legal representative resulting in further delays[19–21,33]. Country-specific variations in insurance and indemnity requirements within the EU were a further hurdle. For example, two studies noted that the minimum compensation in Germany was 500,000 euros and the insurance provided by the sponsor did not meet these limits. Additional insurance coverage could not be provided locally and, consequently, those sites were either dropped or authors had to run two parallel trials in different continents[23,24].

Funding

Fourteen studies described funding as a challenge[19–24,26,28,34–39]. Interestingly, all of these were funded through government sources. Limitations in funding meant that some investigators had to seek additional sources of

For peer review only

support and in cases where this was unsuccessful, there was a delay in trial start up[20,21,37,38]. Additionally, trials funded by the US had a requirement for non-US sites to obtain departmental clearance and approval from their respective countries before funds could be transferred. A lack of familiarity with this process prevented timely transfer of funds and thus delayed recruitment[26]. Predicting accurate budget projections and variations in currency exchange rates were additional challenges for trials spanning several years[34]. Prolonged negotiations between the funder, site and sponsor regarding site set-up costs led to further delays[33].

Lack of harmonisation in ethics and regulatory approvals

A lack of harmonisation in international legal and ethical systems was a reoccurring theme[11,19,21,22,25–29,33,34,38–43]. The time from initiation of regulatory procedures to the start of the trial ranged from 3 to 18 months. Hurdles included: lack of a centralised system and therefore the requirement for single-centre approvals[7,19,22,24,26,34,41], country-specific differences in requirements[7,22,24,26,38,42,43], infrequent ethics committee meetings[22,35], ethics review fee[22,38], translation of essential documents[42], protocol amendments[37,38] and lack of familiarity among ethics committees with trial design or conduct[22,41]. Additionally, multinational trials funded in total or in part by US National Institute of Health required Federalwide Assurance (FWA) approvals and annual reviews alongside adherence to country-specific regulations. Although this process was familiar to US sites, non-US sites found this challenging[7,11,23,26].

Site set-up

Training

In general, staff training was not considered a challenge[20,29,34,37,40,44–49]. Studies used a combination of in-person and online workshops which covered a broad range of topics including: study protocol, Good Clinical Practice, data management, intervention delivery and safety reporting[20,26,35,47,48]. One trial adopted a system of "train-the-trainer," whereby site leads would receive centralised training then train all personnel at their local sites[45].

Contracts

Contracts were formed at many levels including site agreements, funding agreements, sponsorship agreements, agreements with pharmaceutical companies and data sharing. Several studies described contract negotiations as a time consuming and lengthy process[19–21,23,24,28,29,33]. Contributing factors to this were: translation of

For peer review only - http://bmjopen.bmj.com/site/about/guidelines.xhtml

contracts[26], conflict with country-specific legal terminology and interpretation[20,21], clauses of indemnification[19,20] and administrative bureaucracy in legal departments[33]. Data sharing across borders requires additional levels of consent and protection. Trials with EU sites described how all participating sites were required to comply with the EU General Data Protection Regulations (GDPR)[21,24]. This required additional administrative steps[24]. To overcome these challenges, INSIGHT (International Network for Strategic Initiatives in Global HIV Trials) established coordinating centres in several countries that were geographically closer to sites, familiar with local regulatory requirements and fluent in local languages which improved the contract negotiation process[23].

Site monitoring

Monitoring and auditing of sites was conducted periodically and included checking for compliance with the protocol and standard operating procedures (SOPs), data consistency and missing data. Most studies adopted a combination of remote and in-person quality assurance visits and created summary reports that were circulated to sites[35,40,50–52]. Underperforming sites were asked to submit an action plan and provide additional staff training[50,52]. A clear escalation procedure was established for ensuring timely improvement of site performances[50]. Studies reported the importance of planning adequate human and financial resources for these tasks [30,38].

Communication

Regular communication with sites and investigators was critical[20,29,34–36,44,50,53]. Multiple communication channels were utilised to ensure consistency across sites, overcome time zone and cross-cultural differences. These included emails for study protocols, newsletters, progress reports and site score cards, and regular verbal communication in the form of monthly site teleconferences, outreach calls with leadership team and biannual or annual face-to-face meetings[7,20,34,36,44,50,53]. Communications were usually managed by a contract research organization, clinical trials unit or the site selection committee[48,50]. One study described in detail the guidelines they created to streamline the management of site queries and thereby limit unnecessary or excessive email traffic[34].

Trial management

For peer review only

Trial oversight

A number of management "tiers" could be identified, according to their scope of responsibility for trial oversight. Most studies described a Central Coordinating Centre (also referred to as a "trial coordinating centre", "main operational centre" or "clinical or data coordinating centre"; CCC) with over-arching responsibility for overseeing trial setup and conduct in accordance with the protocol, SOPs and regulatory approvals[11,23,25,26,29,38,39,45,48,50–53]. The CCC worked with at least one national coordinating centre (also referred to as an "country coordinating centre" or "national project manager") that supported regulatory approvals, set-up of sites and delivery of the trial in each country[19,25,33,44]. In trials spanning multiple continents, an additional level of management with an international coordinating centre worked closely with the CCC and was responsible for oversight of several national coordinating centres[11,23,54]. This arrangement ensured that there were data and regulatory consistencies between countries or research networks[44,51–53]. In general, the CCC consisted of the chief investigator, biostatistician, and support staff, being responsible for regulatory oversight, recruitment/retention [36], trial monitoring[19,33,45,48,51,52], secondary analyses and communication[11,23,36,50]. Other roles carried out by the CCC included overseeing finance and logistics including drug distribution, storage and analyses of specimen and community support[11,23,25]. Independent overall oversight of was provided by Data Safety Monitoring Board (or Committee; DSMB) and Trial Steering Committee (TSC) [53]. Most study reports referred to the International Conference on Harmonization Good Clinical Practice guidelines (ICH-GCP)[28,35,40,42,43,48,51,52].

Site and intervention selection

Site selection was often based upon initial feasibility assessments[43,46,49]. Several factors were considered during the assessment including local capacity or infrastructure[7,37,39,43,53], prevalence or burden of the disease in question[7,39,43], local ethics committees and regulatory processes[35,40,43,49], individual sites' interest in participating[7], level of expertise available, and insight from local sites representatives[50]. The choice of intervention was usually based on expert reviews[7,44] or a systematic literature review[25,52] which was overseen by the TSC. Some studies also considered treatment guidelines[19,33] and expert consensus where evidence was sparse[41].

Recruitment

For peer review only - http://bmjopen.bmj.com/site/about/guidelines.xhtml

Recruitment strategies varied across the studies. Factors contributing to the difficulty in enrolling and retaining participants were complex and lengthy screening processes[27,49], financial constraints[37], difficulty in maintaining long-term site cooperation[49] and the rigour of navigating varied health systems and setups across countries. Country-specific laws further hampered recruitment: in a German study, participants could only enrol in one trial at a time[33]. Reported strategies that helped to overcome these challenges were frequent communication with participants through a combination of letters, phone calls, mailing of local medical publications containing the trial details and increasing study visibility through the use of websites[34,36,49,54] as well as implementation of the intervention at a convenient location[34]. Some studies organised educational sessions in the form of grand rounds and presentations to healthcare providers which increased visibility[34,36]. Reimbursement of transport costs and keeping visits to a minimum also increased recruitment[36,43].

Data management

Data management was not a major challenge and was generally overseen by the Data Coordinating Centre (DCC)[27,30,34,38,40,45,52]. Data was usually collected at trial sites using data collection forms and stored locally or in a web-based system. Studies which used the local storage model requested trial sites to securely send data to the DCC where it was amalgamated at regular intervals[34,38,45]. In contrast, use of a web-based system provided real time data entry, study updates and improved access[47,51,52,54]. Some studies reported technical challenges including problems with internet or firewalls, delays in data collection and missing data[34,40]. This was managed by ensuring sites had access to technical and training support[27,40].

Intervention management

Drug procurement and distribution

Pharmaceutical companies were the most common source of drug procurement[7,19,26,27,33,38,52,54], and in some cases the investigational medicinal product (IMP) was supplied free of charge[19,33,54]. In instances where there were drug import restrictions, local pharmacies and suppliers were used[7,36]. Most studies established a clinical coordinating pharmacy to oversee the translation, labelling, repackaging, shipping, and coordination of the drug distribution to the study sites[19,24,25,33,39,48]. Country-specific repository centres could be created, particularly in cross-continent studies. These handled the different regulatory and import requirements in addition to providing oversight and support for the distribution of IMPs across sites in their jurisdiction[19,33,48]. More

For peer review only - http://bmjopen.bmj.com/site/about/guidelines.xhtml

often, studies were faced with difficulty in navigating complex regulatory procedures between countries. For example, some studies in the EU were refused waiver for labelling requirements of a repurposed drug whereas a similar waiver request was granted in the UK[19,20,28,33]. Also, there were country-specific differences in import requirements and issues around "qualified person" drug release specifications[19,52]. Site pharmacies were involved in drug dispensing in some studies[26,29,36,48,49,51,52]. SOPs and training were provided to hospital pharmacists at each study site on drug procurement, dispensing, storage, maintenance of accountability logs and disposal of unused or expired drugs[7,51,52].

Safety monitoring and adverse events

Serious and adverse events (SAEs) at sites were reported either directly to the CCC or via an electronic data capture or web-based system, then to sponsor who reported to the relevant ethical and regulatory authority. One study ensured that a study coordinator, physician and safety officer were available at all times to manage emergency situations[36].

All 38 studies established a DSMB for monitoring drug safety and efficacy and safeguarding trial participants. SAEs were reviewed by the independent DSMB at varying intervals based on risk. [7,36,41,44]. The timing for These ranged from monthly, to biannually and annually. Some studies used a predetermined interval, for example, in one study the DSMB met every time at least 25-30 participants had a particular data point collected[25]. If there were concerns or SAEs, these meetings were brought forward. All monitoring activities were generally followed by reports which were circulated to all sites[25,53].

Biospecimen processing and transport

A variety of biospecimens were often processed in a different country[27,47,51,54,55]. Several studies asked sites to ship the specimen to the designated central facility for analysis[27,47,51,54,55]. Where trials spanned multiple continents, separate facilities were established in each [24,42,55]. Where a central facility for specimen storage was not feasible, a "virtual biobank" was sometimes established[53]. The collection, handling and processing of specimens were performed in accordance with SOPs[51,53]. Over-collection or under-collection of specimens, increased shipping costs for collection of additional samples, shipments delays, staff shortages and inadequate training were cited as challenges[27,50]. As most of the specimens were shipped to another country for analysis,

For peer review only

regulatory and administrative approvals, coupled with difficulties securing informed consent for international transfer presented additional hurdles[24,42]. Use of an electronic inventory for real-time monitoring of samples, ongoing laboratory training and efficient trial oversight were described as effective mitigation methods.

Adaptive-specific issues for international trials

Adaptive trial design has become a popular means of increasing the flexibility with which interventions may be interrogated with respect to particular disease indications and/or outcomes, and the efficiency with which this may be achieved (for example through interim analyses in the context of platform trials)[15]. They formed a substantial minority of the trials identified in the current review, reflecting challenges raised when implementing them across national boundaries. For example, compared to traditional trials, the need for significantly more documentation to provide clarity on the adaptive process was necessary, including treatment arms, plans for data collection and interim analyses[25,56]. Managing protocol amendments posed a further challenge. Studies reported that using an overarching master protocol and consent form and limiting IMP-specific information to appendices significantly expedited the time for ethics approval when adding or removing treatment arms[25,53]. Handling drug supply for multiple treatment arms whilst minimising waste was another recurring theme. Studies found it helpful to use pharmacies that could serve multiple clinical sites within a close geographical area, a centralised drug distribution system[25] and choosing countries with similar drug labelling requirements[56]. Data systems that enabled rapid data entry and analyses was another important consideration. Studies reported that setting up a streamlined system with pre-specified stopping rules for each treatment arm was helpful[35,53]. They also had predefined timelines for the frequency of data analysis [40,53,56].

Discussion

We conducted a systematic review to determine the operational complexities of conducting international trials, with the aim of providing a useful resource for researchers considering such an approach. Our search strategy employed an extensive array of search terms, themselves falling under four broad "concepts" and organised to optimise our ability to capture all relevant articles; this approach then necessitated significant refinement during the screening phase, and rigorous data collection methods. To this end, each abstract was reviewed by at least two authors for

For peer review only

inclusion and 20% of the full-text articles were screened by a second author. Our review highlights that there are various, consistent challenges in the planning, setup, delivery and close out phases of the 38 international trials. Some of the greatest challenges are posed by ethical and regulatory obstacles, a lack of harmonisation within EU and between EU and other developed countries being a key element. Similar findings have been highlighted in another systematic review on conducting trials in developing countries[57]. Streamlining funding and regulatory processes is one possible solution. The EU Clinical Trials Regulation launched a portal in 2022 that enables registration of trials with sites in up to 30 EU countries in a single platform. This system also resolves data sharing issues, providing a one-off consent and enabling national regulators to collaboratively process approvals[58]. However, there is no obligation for EU countries to participate until 2025 and this process does not provide a solution for collaboration with other continents. Furthermore, this portal does not extend to UK sites following its withdrawal from the EU. Possible interim solutions to global collaboration include defining responsibilities of sponsors and legal representatives, ascertaining insurance requirements prior to country selection and ensuring all sites are provided with a framework for the regulatory review process. In addition, working with a specialist insurance broker and careful country selection based on previous successful collaborations have also been suggested[59]. Establishing a national support group to provide mentoring to less experienced trialists for the entire trial process may also aid in the process[57]. By contrast, previous literature has described other key challenges including lack of infrastructure, poor data quality and lack of training particularly in resource-limited settings. These were not a particular challenge in our systematic review[57,60].

Budgetary constraints and contract negotiations were described as further hurdles. Over the last few years, several networks have been formed to increase funding opportunities. For example, INSIGHT was created to provide governance and funding support for multi-national HIV treatment trials but has expanded to also include influenza and COVID-19[61]. NeuroNEXT (Network for Excellence in Neuroscience Clinical Trials) promotes partnership in neurological disease trials by providing a centralised system for ethics review, contracting agreements and data management but is limited to multi-centre studies in the US[62]. Use of these networks, establishing site set-up costs during the trial planning phase and timely contract negotiations may assist in this process.

Managing drug distribution across international borders remains a challenge, as has also been previously reported[21]. Tailoring supply chains whilst minimising drug wastage is clearly desirable. Establishment of central

For peer review only

drug repositories in each continent and using pharmacies which can serve multiple sites rather than the traditional one-pharmacy-one-site model has been shown to be effective[19,48]. Involving pharmaceutical partners in drug distribution is another possibility which also eliminates the need for time-consuming vendor selection[63].

Our search strategy was employed an extensive array of search terms, themselves falling under four broad "concepts" and organised to optimise our ability to capture all relevant articles; this approach then necessitated significant refinement during the screening phase to arrive at robust and our data collection methods rigorous. Each abstract was reviewed by at least two authors for inclusion and 20% of the full-text articles were screened by a second author.

Limitations and future work.

The extent to which international facets of clinical trials is made explicit in their description, and the terminology used in the literature to describe clinical trials in general, have evolved over time, and this observation is reflected in the pragmatic search and screening strategy we adopted. As with any systematic review, we cannot exclude the possibility that decisions about individual terms included in, or excluded from, our searches may have influenced the precise range of papers pertaining to individual international trials that would have been captured in our systematic review – and hence the challenges and solutions identified during data extraction. Linked to this, not all investigators routinely publish information on challenges encountered in trial design and conduct, meaning our findings cannot be said to be exhaustive. Moreover, our systematic review only focused on trials conducted in more than one country and studies published after 2005 and therefore our results may not be generalisable to multi-centre studies conducted within the same country. For example, ethics regulations, sponsor responsibilities and insurance requirements are less likely to be a hurdle in single nation trials[63]. With respect to potential solutions to the challenges described, these were extracted from included papers where identified in association with specific challenges; whilst every effort was made to mandate this link, an element of subjectivity when summarising such solutions in the current report cannot be excluded. Finally, we did not assess clinical outcomes of included studies, considering this beyond the scope of our endeavour. Further research in the form of a qualitative or Delphi study with trialists and key stakeholders may provide more in-depth information on these challenges and possible actions to mitigate them. We also urge researchers involved in conducting international trials to routinely report operational challenges.

For peer review only

Conclusion

International trials address many unmet needs in trial design but their proponents still face operational challenges at every level, ranging from difficulties with funding and obtaining regulatory approvals to site contracts and drug distribution. Careful planning and communication with sites and key stakeholders during the trial planning phase can overcome delays presented by some of these challenges. More generally, given the upsurge in global trials particularly since the COVID-19 pandemic, recognition by policymakers of the potential rewards that regulatory harmonisation between nations could bring for the delivery of more efficient and cost-effective research should positively impact the health and wellbeing of their citizens. National and international organisations should continue to work collaboratively to develop infrastructures that support international trials.

Ethical approval

Not applicable.

Data sharing

No additional data available.

For peer review only

Tables

Table 1. Summary of included studies

First author (year)	Trial name	Trial design	Population	Number of sites	Site locations	Main coordinating centre(s)	Total participants	Intervention
Aban (2008)[25]	MGTX	Parallel	Myasthenia gravis	79	Global	USA	126	Other
Aitken (2008)[32]	PROMOTION	Parallel	Coronary artery disease	5	North America, Oceania	USA	3522	Behavioural
Angus (2020)[33]	REMAP-CAP	Adaptive-platform	Severe pneumonia and COVID-19		UK/EU, North America, Oceania, Asia	Australia, Thailand		Drug
Aryal (2021)[38]	REMAP-CAP	Adaptive-platform	Severe pneumonia and COVID-19		UK/EU, North America, Oceania, Asia	RCC in Australia and Thailand		Drug
Antic (2015)[42]	SAVE	Parallel	Obstructive sleep apnoea	89	Oceania, North America, South America, Asia, UK/EU	RCC in Australia, Brazil, China, India and Spain	2717	Device
Babiker (2013)[18]	START	Parallel	HIV	237	North America, South America, UK/EU, Oceania, Africa	RCC in Denmark, UK, Australia, USA	4000	Drug
Berthon-Jones (2015)[26]	ALTAIR	Parallel	HIV	36	Asia, Oceania, UK/EU, North America, South America		322	Drug
Bryant (2021)[49]	TBTC Study 31	Parallel	Tuberculosis	34	North America, South America, Asia, Africa	USA	2516	Drug
Carli (2013)[43]	SEYLE	Cluster	Suicide	11	UK/EU	Sweden	11110	Behavioural
Clasen (2020)[53]	HAPIN	Parallel	Low birth weight		Asia, North America, South America, Africa	USA		Device
Coomarasamy (2016)[50]	PROMISE	Parallel	Recurrent miscarriage	45	UK/EU	UK	836	Drug
Crow (2018)[19]	FOR-DMD	Parallel	Duchenne muscular dystrophy	40	North America, UK/EU	USA	196	Drug
del Álamo (2022)[20]								
Denholm (2022)[51]	ASCOT ADAPT	Adaptive-platform	COVID-19		Oceania, Asia			Drug
Dutton (2009)[44]	CONTROL	Parallel	Trauma	75	North America, South America, UK/EU, Asia, Africa	USA	576	Drug
Eikelboom (2022)[39]	ACT	Factorial	COVID-19	62	North America, South America, Africa, Asia		6528	Drug
Fogelholm (2017)[45]	PREVIEW	Factorial	Pre-diabetes	8	UK/EU, Oceania		2326	Behavioural
Franciscus (2014)[52]	TRIGR	Parallel	Type 1 diabetes	77	North America, Oceania, UK/EU	USA	5156	Other

For peer review only

Fulda (2023)[48]	REPRIEVE	Parallel	HIV		North America, South America, Africa, Asia, UK/EU	USA		Drug
Goossens (2021)[27]	REMAP-CAP	Adaptive-platform	Severe pneumonia and COVID-19		UK/EU, North America, Oceania, Asia	Australia, Thailand		Drug
Grarup (2015)[31]	START	Parallel	HIV	237	North America, South America, UK/EU, Oceania, Africa	RCC in Denmark, UK, Australia, USA	4000	Drug
Hata (2020)[40]	PATHWAY	Parallel	Breast cancer	23	Asia		185	Drug
Herrick (2012)[34]	FDTT	Parallel	Functional dyspepsia	8	North America		292	Drug
Jeon (2016)[46]	CLEAR III	Parallel	Intracerebral haemorrhage	73	North America, South America, UK/EU, Asia	USA	500	Drug
Kenyon (2011)[21]	STICH II	Parallel	Intracerebral haemorrhage	126	North America, Oceania, UK/EU, Asia, Africa	UK	601	Other
Kesho Bora Study Group (2011)[35]	Kesho-Bora	Parallel	HIV	5	Africa	Switzerland	824	Drug
Kolitsopoulos (2013)[47]	ZODIAC	Parallel	Schizophrenia	226	North America, South America, UK/EU, Asia		18240	Drug
Larson (2016)[22]	INSIGHT trials					RCC in UK, Denmark, USA and Australia		
Lingor (2021)[23]	ROCK-ALS / ROCK-ALS-US	Parallel	Amyotrophic lateral sclerosis		North America, UK/EU			Drug
Minisman (2012)[5]	MGTX	Parallel	Myasthenia gravis	79	Global	USA	126	Other
Murray (2022)[24]	TICO	Adaptive-platform	COVID-19		North America, UK/EU, Asia, Africa	USA with 8 RCC		Drug
Neaton (2010)[9]	INSIGHT trials					RCC in UK, Denmark, USA and Australia		
Ravinetto (2013)[36]	4ABC	Parallel	Malaria	12	Africa	Belgium	4112	Drug
Reams (2018)[41]	DOVE	Parallel	Sickle cell disease	51	North America, South America, UK/EU, Asia, Africa		341	Drug
Seal (2006)[28]		Factorial	Endophthalmitis	24	UK/EU, Asia	UK	35000	Drug
Spencer (2012)[54]	AWARD-5	Adaptive	Type 2 diabetes	111	North America, UK/EU, Asia		1202	Drug
Sydes (2011)[29]	STAMPEDE	Adaptive-platform	Prostate cancer		UK/EU	UK		Drug
Zimmer (2010)[37]	BAMSG 3-01	Parallel	Cryptococcal meningitis	13	North America, Asia	USA	143	Drug

RCC: Regional Coordinating Centre; RCT: Randomised Controlled Trial.

For peer review only

Table 2. Design characteristics of included trials (n=35)

**Features**	**n (%)**
**Design**	
Parallel	24 (68.6)
Adaptive	7 (20.0)
Cluster	1 (2.9)
Factorial	3 (8.6)
**Masking**	
Open label	19 (54.3)
Blinded	16 (45.7)
**Number of sites among closed trials**: median (IQR)	40 (13 – 78)
**Continents of site locations**	
Africa	14 (40.0)
Asia	22 (62.9)
North America	27 (77.1)
Oceania	14 (40.0)
South America	14 (40.0)
UK/Europe	25 (71.4)
**Trial status**	
Open	9 (25.7)
Closed	25 (71.4)
Terminated	1 (2.9)
**Sample size among closed trials**: median (IQR)	1202 (332 – 4056)
**Intervention**	
Drug	26 (74.3)
Device	2 (5.7)
Behavioural	3 (8.6)
Other	4 (11.4)
**Primary outcome**	
Efficacy	14 (40.0)
Effectiveness	12 (34.3)
Safety	2 (5.7)
Prevention	7 (20.0)
**Sponsor**	
Academic	31 (88.6)
Industry	3 (8.6)
Unknown	1 (2.9)
**Main funder**	
Academic	1 (2.9)
Government	28 (80.0)
Industry	5 (14.3)
Charity	1 (2.9)

For peer review only

Table 3. Challenges and proposed solutions		
	**Challenges**	**Proposed solutions**
Study set-up	Identification of appropriate sponsor and agreements	• Clarify roles and responsibilities between sponsor, co-sponsor and legal representative in advance<br>• Ascertain insurance policy requirements prior to country and site selection<br>• Identify a lead site for each country/continent that is familiar with local regulations to act as a coordinating centre for all local sites
	Budget considerations	• Establish site set-up costs early on (including start-up fees, pharmacy dispensing, lab, ethics, administration, archiving and close out)<br>• Consideration of changes in currency exchange rates<br>• Accurate budget predictions for trials spanning several years<br>• Translation costs
	Regulatory and ethical approvals	• Ensure all sites are familiar with the process required for regulatory approvals
Site set-up	Contracts with collaborating sites	• Begin contract negotiations early<br>• Establish coordinating sites or centres within each country or region to manage negotiations<br>• Allocate time and costs for translation<br>• Coordination with legal representatives to clarify country-specific terminology and interpretation of legal responsibilities and GDPR
	Site monitoring	• Plan adequate financial and human resources for in-person visits<br>• Clear escalation procedures and remedial actions for sites which are underperforming
	Channels of communication	• Regular communication with sites and investigators using multiple channels including emails, monthly teleconferences, outreach calls and annual face-to-face meetings<br>• Develop a hierarchical process for managing site queries to avoid unnecessary emails
	Translation	• Plan adequate financial resources and time for translation of essential documents, contracts and drug packaging
Trial management	Site and intervention selection	• Conduct site feasibility assessments during trial planning phase<br>• Use a systematic process to guide intervention selection and removal particularly for adaptive trials
	Recruitment and retention	• Frequent, often weekly communication with study participants using different channels (e.g. letters, phone calls, emails)<br>• Reimbursement of transport costs<br>• Keeping study visits to a minimum
Data management	Technological challenges including internet bandwidth, institutional firewalls and device availability	• Access to technical expertise and availability of real-time troubleshooting all the time<br>• Use of a software with an option for offline data entry
	Delays in data entry and missing data	• Training and retraining of researchers in data entry and protocol compliance
Intervention management	Difficulty in getting import permits	• Use local commercial or pharmacy suppliers
	Drug wastage	• Distribute smaller numbers but frequently replenish study sites
	Shipment delays and errors	• Establish an electronic inventory that allows real time monitoring of drug stock, biospecimen collection and distribution<br>• Staff training
Adaptive specific	Protocol amendments	• Use an overarching master protocol and consent form and add agent-specific information in appendices to speed up ethical reviews
	Handling drug supply for multiple arms	• Use pharmacies that can serve multiple sites within a close geographical area<br>• Centralised drug distribution system<br>• Choose countries with similar drug labelling requirements
	Data entry and analyses	• Predefine stopping rules for each treatment arm<br>• Predefine frequency of data analysis

For peer review only

Figure legends

Figure 1. PRISMA flow diagram.

Figure 2. Operational complexities of international trials.

References

1 Glickman SW, McHutchison JG, Peterson ED, *et al.* Ethical and Scientific Implications of the Globalization of Clinical Research. *N Engl J Med*. 2009;360:816–23.

2 Weigmann K. The ethics of global clinical trials: In developing countries, participation in clinical trials is sometimes the only way to access medical treatment. What should be done to avoid exploitation of disadvantaged populations? *EMBO Rep*. 2015;16:566–70.

3 Barrios CH, Werutsky G, Martinez-Mesa J. The global conduct of cancer clinical trials: challenges and opportunities. *Am Soc Clin Oncol Educ Book Am Soc Clin Oncol Annu Meet*. 2015;e132-139.

4 World Health Organization. WHO COVID-19 Solidarity Therapeutics Trial. 2021. https://www.who.int/emergencies/diseases/novel-coronavirus-2019/global-research-on-novel-coronavirus-2019-ncov/solidarity-clinical-trial-for-covid-19-treatments (accessed 23 March 2023)

5 Lang T, Siribaddana S. Clinical trials have gone global: is this a good thing? *PLoS Med*. 2012;9:e1001228.

6 Thiers FA, Sinskey AJ, Berndt ER. Trends in the globalization of clinical trials. *Nat Rev Drug Discov*. 2008;7:13–4.

7 Minisman G, Bhanushali M, Conwit R, *et al.* Implementing clinical trials on an international platform: challenges and perspectives. *J Neurol Sci*. 2012;313:1–6.

8 Jeong S, Sohn M, Kim JH, *et al.* Current globalization of drug interventional clinical trials: characteristics and associated factors, 2011–2013. *Trials*. 2017;18:288.

9 The Adaptive Platform Trials Coalition. Adaptive platform trials: definition, design, conduct and reporting considerations. *Nat Rev Drug Discov*. 2019;18:797–807.

10 Pocock S, Calvo G, Marrugat J, *et al.* International differences in treatment effect: do they really exist and why?†. *Eur Heart J*. 2013;34:1846–52.

11 Neaton JD, Babiker A, Bohnhorst M, *et al.* Regulatory impediments jeopardizing the conduct of clinical trials in Europe funded by the National Institutes of Health. *Clin Trials*. 2010;7:705–18.

12 Tang M, Joensuu H, Simes RJ, *et al.* Challenges of international oncology trial collaboration-a call to action. *Br J Cancer*. 2019;121:515–21.

13 Kurzrock R, Pilat S, Bartolazzi M, *et al.* Project Zero Delay: a process for accelerating the activation of cancer clinical trials. *J Clin Oncol Off J Am Soc Clin Oncol*. 2009;27:4433–40.

14 Lai J, Forney L, Brinton DL, *et al.* Drivers of Start-Up Delays in Global Randomized Clinical Trials. *Ther Innov Regul Sci*. 2021;55:212–27.

15 Chow S-C, Corey R. Benefits, challenges and obstacles of adaptive clinical trial designs. *Orphanet J Rare Dis*. 2011;6:79.

For peer review only

16 Brown LC, Graham J, Fisher D, *et al.* Experiences of running a stratified medicine adaptive platform trial: Challenges and lessons learned from 10 years of the FOCUS4 trial in metastatic colorectal cancer. *Clin Trials*. 2022;19:146–57.

17 Page MJ, McKenzie JE, Bossuyt PM, *et al.* The PRISMA 2020 statement: an updated guideline for reporting systematic reviews. *BMJ*. 2021;n71.

18 Gumber L, Pratt A, Bardgett M, *et al.* Operational best practice in international clinical trials: a systematic review protocol. Published Online First: 30 January 2023. doi: 10.17605/OSF.IO/YVTJB

19 Babiker AG, Emery S, Fatkenheuer G, *et al.* Considerations in the rationale, design and methods of the Strategic Timing of AntiRetroviral Treatment (START) study. *Clin Trials*. 2013;10:S5–36.

20 Crow RA, Hart KA, McDermott MP, *et al.* A checklist for clinical trials in rare disease: obstacles and anticipatory actions-lessons learned from the FOR-DMD trial. *Trials Electron Resour*. 2018;19:291.

21 Del Alamo M, Buhrer C, Fisher D, *et al.* Identifying obstacles hindering the conduct of academic-sponsored trials for drug repurposing on rare-diseases: an analysis of six use cases. *Trials Electron Resour*. 2022;23:783.

22 Kenyon GM, Mendelow AD, Gregson BA, *et al.* Obtaining regulatory approval for multicentre randomised controlled trials: experiences in the STICH II trial. *Br J Neurosurg*. 2011;25:352–6.

23 Larson GS, Carey C, Grarup J, *et al.* Lessons learned: Infrastructure development and financial management for large, publicly funded, international trials. *Clin Trials*. 2016;13:127–36.

24 Lingor P, Koch JC, Statl, *et al.* Challenges and opportunities for Multi-National Investigator-Initiated clinical trials for ALS: European and United States collaborations. *Amyotroph Lateral Scler Front Degener*. 2021;22:419–25.

25 Murray DD, Babiker AG, Baker JV, *et al.* Design and implementation of an international, multi-arm, multi-stage platform master protocol for trials of novel SARS-CoV-2 antiviral agents: Therapeutics for Inpatients with COVID-19 (TICO/ACTIV-3). *Clin Trials*. 2022;19:52–61.

26 Aban IB, Wolfe GI, Cutter GR, *et al.* The MGTX experience: challenges in planning and executing an international, multicenter clinical trial. *J Neuroimmunol*. 2008;201:80–4.

27 Berthon-Jones N, Courtney-Vega K, Donaldson A, *et al.* Assessing site performance in the Altair study, a multinational clinical trial. *Trials Electron Resour*. 2015;16:138.

28 Goossens H, Derde L, Horby P, *et al.* The European clinical research response to optimise treatment of patients with COVID-19: lessons learned, future perspective, and recommendations. *Lancet Infect Dis*. 2022;22:e153–8.

30 Seal DV, Barry P, Gettinby G, *et al.* ESCRS study of prophylaxis of postoperative endophthalmitis after cataract surgery. Case for a European multicenter study. *J Cataract Refract Surg*. 2006;32:396–406.

31 Sydes MR, Parmar MK, Mason MD, *et al.* Flexible trial design in practice - stopping arms for lack-of-benefit and adding research arms mid-trial in STAMPEDE: a multi-arm multi-stage randomized controlled trial. *Trials Electron Resour*. 2012;13:168.

32 Official Journal of the European Union. Regulation (EU) No 536/2014 of the European Parliament and of the Council of 16 April 2014 on clinical trials on medicinal products for human use, and repealing Directive 2001/20/EC Text with EEA relevance. 2022. https://eur-lex.europa.eu/legal-content/EN/TXT/?uri=CELEX%3A02014R0536-20221205 (accessed 4 May 2023)

For peer review only

33 Grarup J, Rappoport C, Engen NW, *et al.* Challenges, successes and patterns of enrolment in the INSIGHT Strategic Timing of AntiRetroviral Treatment (START) trial. *HIV Med*. 2015;16:14–23.

34 Aitken LM, Pelter MM, Carlson B, *et al.* Effective strategies for implementing a multicenter international clinical trial. *J Nurs Scholarsh*. 2008;40:101–8.

35 Angus DC, Berry S, Lewis RJ, *et al.* The REMAP-CAP (Randomized Embedded Multifactorial Adaptive Platform for Community-acquired Pneumonia) Study. Rationale and Design. *Ann Am Thorac Soc*. 2020;17:879–91.

36 Herrick LM, Locke 3rd GR, Zinsmeister AR, *et al.* Challenges and lessons learned in conducting comparative-effectiveness trials. *Am J Gastroenterol*. 2012;107:644–9.

37 Kesho Bora Study G. Safety and effectiveness of antiretroviral drugs during pregnancy, delivery and breastfeeding for prevention of mother-to-child transmission of HIV-1: the Kesho Bora Multicentre Collaborative Study rationale, design, and implementation challenges. *Contemp Clin Trials*. 2011;32:74–85.

38 Ravinetto RM, Talisuna A, De Crop M, *et al.* Challenges of non-commercial multicentre North-South collaborative clinical trials. *Trop Med Int Health*. 2013;18:237–41.

39 Zimmer LO, Nolen TL, Pramanpol S, *et al.* International collaboration between US and Thailand on a clinical trial of treatment for HIV-associated cryptococcal meningitis. *Contemp Clin Trials*. 2010;31:34–43.

40 Aryal D, Beane A, Dondorp AM, *et al.* Operationalisation of the Randomized Embedded Multifactorial Adaptive Platform for COVID-19 trials in a low and lower-middle income critical care learning health system. *Wellcome Open Res*. 2021;6:14.

41 Eikelboom J, Rangarajan S, Jolly SS, *et al.* The Anti-Coronavirus Therapies (ACT) Trials: Design, Baseline Characteristics, and Challenges. *CJC Open*. 2022;4:568–76.

42 Hata T, Nakamura K, Yonemori K, *et al.* Regulatory and operational challenges in conducting Asian International Academic Trial for expanding the indications of cancer drugs. *Clin Transl Sci*. 2021;14:1015–25.

43 Reams SG, Messana L, Brown PB, *et al.* Clinical trial conduct in special populations and developing regions: An overview of the DOVE study in pediatric patients with sickle cell disease. *Contemp Clin Trials*. 2018;64:88–94.

44 Antic NA, Heeley E, Anderson CS, *et al.* The Sleep Apnea cardioVascular Endpoints (SAVE) Trial: Rationale, Ethics, Design, and Progress. *Sleep*. 2015;38:1247–57.

45 Carli V, Wasserman C, Wasserman D, *et al.* The saving and empowering young lives in Europe (SEYLE) randomized controlled trial (RCT): methodological issues and participant characteristics. *BMC Public Health*. 2013;13:479.

46 Dutton R, Hauser C, Boffard K, *et al.* Scientific and logistical challenges in designing the CONTROL trial: recombinant factor VIIa in severe trauma patients with refractory bleeding. *Clin Trials*. 2009;6:467–79.

47 Fogelholm M, Larsen TM, Westerterp-Plantenga M, *et al.* PREVIEW: Prevention of Diabetes through Lifestyle Intervention and Population Studies in Europe and around the World. Design, Methods, and Baseline Participant Description of an Adult Cohort Enrolled into a Three-Year Randomised Clinical Trial. *Nutrients*. 2017;9:20.

For peer review only

48 Jeon JE, Mighty J, Lane K, *et al.* Participation of a coordinating center pharmacy in a multicenter international study. *Am J Health Syst Pharm*. 2016;73:1859–68.

49 Kolitsopoulos FM, Strom BL, Faich G, *et al.* Lessons learned in the conduct of a global, large simple trial of treatments indicated for schizophrenia. *Contemp Clin Trials*. 2013;34:239–47.

50 Fulda ES, Fichtenbaum CJ, Kileel EM, *et al.* The importance of methods for site performance evaluation in REPRIEVE, a longitudinal, global, multicenter trial. *Contemp Clin Trials*. 2023;124. doi: 10.1016/j.cct.2022.107035

51 Bryant KE, Yuan Y, Engle M, *et al.* Central monitoring in a randomized, open-label, controlled phase 3 clinical trial for a treatment-shortening regimen for pulmonary tuberculosis. *Contemp Clin Trials*. 2021;104. doi: 10.1016/j.cct.2021.106355

52 Coomarasamy A, Williams H, Truchanowicz E, *et al.* PROMISE: First-trimester progesterone therapy in women with a history of unexplained recurrent miscarriages - A randomised, double-blind, placebo-controlled, international multicentre trial and economic evaluation. *Health Technol Assess*. 2016;20:7–91.

53 Denholm JT, Venkatesh B, Davis J, *et al.* ASCOT ADAPT study of COVID-19 therapeutics in hospitalised patients: an international multicentre adaptive platform trial. *Trials Electron Resour*. 2022;23:1014.

54 Franciscus M, Nucci A, Bradley B, *et al.* Recruitment and retention of participants for an international type 1 diabetes prevention trial: a coordinators' perspective. *Clin Trials*. 2014;11:150–8.

55 Clasen T, Checkley W, Peel JL, *et al.* Design and Rationale of the HAPIN Study: A Multicountry Randomized Controlled Trial to Assess the Effect of Liquefied Petroleum Gas Stove and Continuous Fuel Distribution. *Environ Health Perspect*. 2020;128:47008.

56 Spencer K, Colvin K, Braunecker B, *et al.* Operational challenges and solutions with implementation of an adaptive seamless phase 2/3 study. *J Diabetes Sci Technol*. 2012;6:1296–304.

57 Alemayehu C, Mitchell G, Nikles J. Barriers for conducting clinical trials in developing countries- a systematic review. *Int J Equity Health*. 2018;17:37.

58 European Medicines Agency. Clinical Trials Regulation. Eur. Med. Agency. 2022. https://www.ema.europa.eu/en/human-regulatory/research-development/clinical-trials/clinical-trials-regulation (accessed 25 April 2023)

59 Fox L, Toms C, Kernaghan S, *et al.* Conducting non-commercial international clinical trials: the ICR-CTSU experience. *Trials Electron Resour*. 2017;18:440.

60 Mbuagbaw L, Thabane L, Ongolo-Zogo P, *et al.* The challenges and opportunities of conducting a clinical trial in a low resource setting: the case of the Cameroon mobile phone SMS (CAMPS) trial, an investigator initiated trial. *Trials*. 2011;12:145.

61 International Network for Strategic Initiative in Global HIV Trials. New science in the International Network for Strategic Initiatives in Global HIV Trials (INSIGHT): Why, What and How. 2011. http://insight.ccbr.umn.edu/governance_committees/ssc/WhyWhatWhoToDoResearch.pdf (accessed 9 March 2023)

62 Love MN, Fathallh-Shaykh H. Network for Excellence in Neuroscience Clinical Trials: NeuroNEXT. *JAMA Neurol*. Published Online First: 5 August 2013. doi: 10.1001/jamaneurol.2013.3663

63 Snowdon C, Kernaghan S, Moretti L, *et al.* Operational complexity versus design efficiency: challenges of implementing a phase IIa multiple parallel cohort targeted treatment platform trial in advanced breast cancer. *Trials*. 2022;23:372.

For peer review only

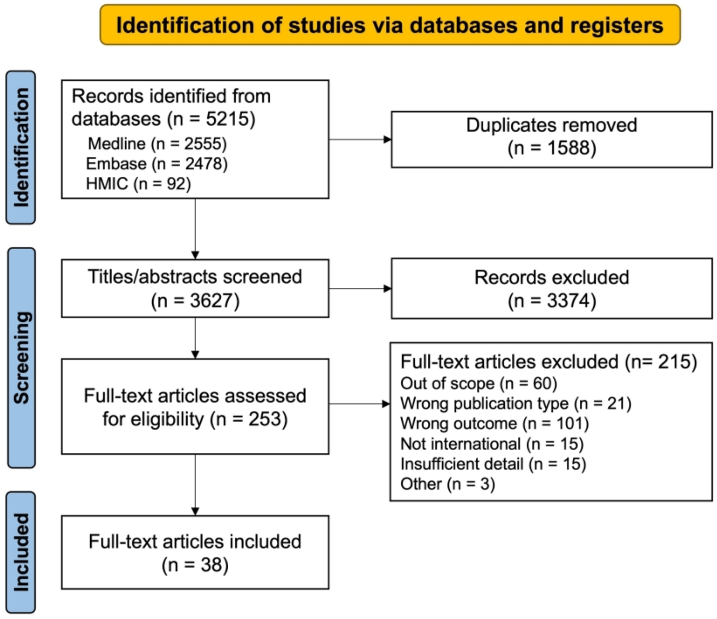

PRISMA Flow diagram

254x190mm (300 x 300 DPI)

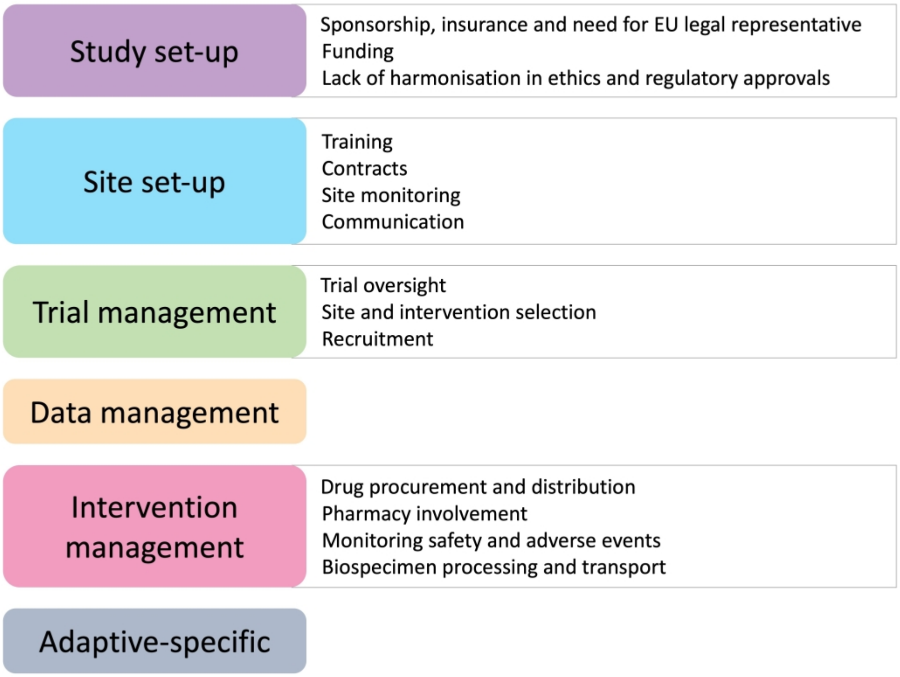

Figure 2. Operational complexities of international trials

254x190mm (400 x 400 DPI)

Supplementary Material

1 LITERATURE SEARCH STRATEGY

A summary of the search and selection strategy is first depicted for quick reference (a); examples of the kinds of search terms that were used to identify each of four concepts, as described in the main manuscript are then provided in (b). Full and detailed database search strategies used in Medline, Embase and HMIC are then also provided (c) for reference.

a) Search and selection strategy

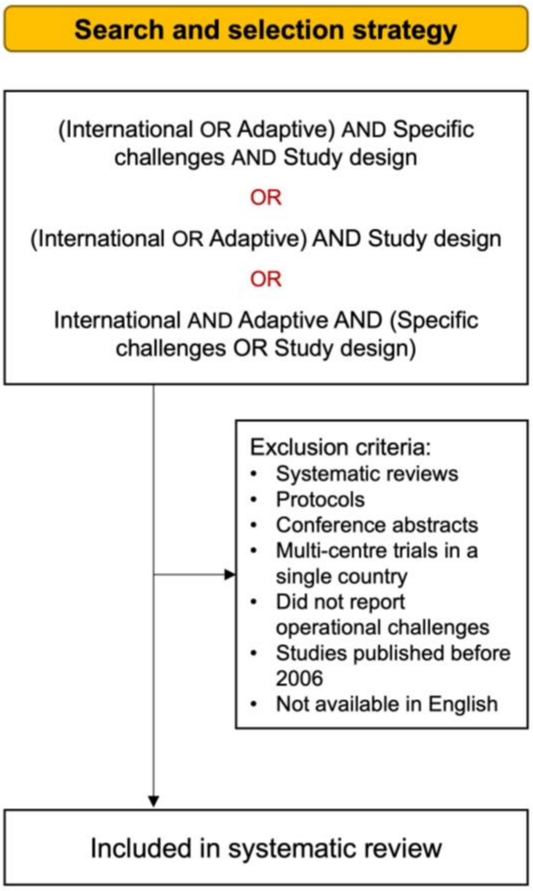

b) Examples of search terms used for each concept

International
International, multinational, intercontinental, multicentre, multisite, pan-European.

Adaptive
Adaptive, platform, umbrella, basket, bucket, master protocol.

Specific challenges
legal, operational, workforce, data management, insurance, indemnity, add or remove arms, gdpr, infrastructure, recruitment, sponsor, contract research, law, regulation, budget, finance, resource allocation, resource management, policy, Brexit, intervention, investigational, supply, logistics, distribution, delivery, monitoring, safety, capacity building, contract, funding, delegation, agreement, ethics, insurance, data sharing.

Study design
lessons, pitfalls, study design, methodology, challenge, operational, practical, guidelines, recommendations, rationale, implement, workflow, framework.

For peer review only - http://bmjopen.bmj.com/site/about/guidelines.xhtml

c) Detailed database search strategy.

Database: MEDLINE (Ovid), 31 January 2023

1	adaptive clinical trial/	37
2	((adaptive or umbrella or basket or bucket) adj2 (design or trial or trials or study or studies or protocol$)).mp.	4428
3	(trial$ platform$ or platform design or platform trial$ or platform clinical trial$ or platform study or platform studies or platform protocol$).mp.	856
4	((adaptive adj6 design$) and (trial or trials or study or studies or protocol$)).mp.	3077
5	(complex innovative adj3 (trial$ or design$ or protocol$)).mp.	17
6	master protocol$.mp.	242
7	response adaptive randomi$.mp.	139
8	(single centre or single site).mp.	33986
9	(or/1-7) not 8 [adaptive design - not single-site]	6597
10	(international adj5 (trial or trials or protocol$ or multicent$ or multi-cent$ or multisite or multi-site or multi-arm or multiarm or multi-stage or multistage)).mp.	19111
11	(international clinical$ trial$ regist$ or international clinical$ trial$ platform$ or international standard randomi$ control$ trial$ number$).mp.	3897
12	international.mp. /freq=2	93924
13	10 not (11 not 12) [gets rid of results with 'international trial' that only refer to the WHO ICTRP]	15678
14	((multinational or multi-national or intercontinental or inter-continental or pan-europ$) adj8 (trial or trials or protocol$ or multicent$ or multi-cent$ or multisite or multi-site)).mp.	3011
15	((trial or trials or protocol$) and (((multicent$ or multi-cent$ or multisite or multi-site) and sites) or centres or centers) and countries).mp.	5427
16	((UK or United Kingdom) and (EU or Europ$) and (trial$ or protocol$)).mp.	2982
17	(europe/ or european alpine region/ or andorra/ or austria/ or balkan peninsula/ or belgium/ or exp europe, eastern/ or exp france/ or exp germany/ or gibraltar/ or greece/ or ireland/ or exp italy/ or liechtenstein/ or luxembourg/ or exp mediterranean region/ or monaco/ or netherlands/ or portugal/ or san marino/ or exp "scandinavian and nordic countries"/ or spain/ or switzerland/ or exp transcaucasia/ or exp ussr/ or vatican city/) and exp United Kingdom/ and (trial$ or protocol$).mp.	1404
18	(exp africa/ or exp americas/ or exp asia/ or exp oceania/) and exp europe/ and (trial$ or protocol$).mp.	9851
19	or/13-18 [main international trial requirement]	35699
20	Methods/	231754
21	Research Design/	122769
22	challenge$.mp.	870696
23	(((trial$ or design) adj5 efficiency) or efficiencies).mp.	54394
24	((complexit$ or guidance or guidelines or recommendations or considerations or issues or obstacles or barriers) and trial$).ti.	5122
25	((lesson or lessons or pitfall or pitfalls) and (design$ or plan$)).mp.	27395
26	(trial$ adj5 (manag$ or run or running or conducting)).mp.	15671
27	(design$ or rationale$ or implement$ or methodol$).ti.	292767
28	(trial$ and (framework$ or regulations)).ti.	488
29	(trials and (Europ$ or EU or multinational or multi-national or intercontinental or inter-continental or international or multi-cent$ or multicent$ or multi-site or multisite)).ti.	2853
30	(trial$ adj1 (approval or authorisation or authorization or regulation)).mp.	341
31	"organization and administration"/ or capacity building/ or decision making, organizational/ or efficiency/ or efficiency, organizational/ or organizational culture/ or workforce/ or models, organizational/ or organizational innovation/ or change management/ or organizational objectives/ or personnel management/ or leadership/ or personnel selection/ or "personnel staffing and scheduling"/ or staff development/ or planning techniques/ or strategic planning/ or program development/ or total quality management/	306062
32	Multicenter Studies as Topic/	22031
33	exp *Clinical Trials as Topic/ or exp Clinical Trials as Topic/mt, st	68990
34	(trial$ and design$).kf.	2312

For peer review only

35	(((good or best) adj3 practice$) and trial$).ti.	204
36	(trial$ adj2 (authoris$ or authoriz$)).mp.	110
37	or/20-36 [general study design/methodology terms]	1888239
38	((trial$ adj5 insur$) or insuring or indemni$).mp.	2674
39	((add$ or remov$) adj5 (arm or arms)).mp.	3631
40	gdpr.mp.	365
41	exp *ethics/ or ethic$.ti.	121779
42	((data adj8 secur$) or (data adj3 (share$ or sharing)) or data management or data governance).mp. or computer security/ or data anonymization/	33659
43	data accuracy/	3708
44	(trial$ adj3 infrastructure).mp.	197
45	exp *models, statistical/ or (exp models, statistical/ and ((statistical adj3 (model$ or framework$ or plan$)) or estimand$).ti,ab.) or (statistical adj1 design$).mp.	59261
46	software/ or database management systems/ or software design/ or software validation/	133275
47	(reporting guideline$ or outcome reporting).kf. or reporting.ti. or (reporting adj4 (quality or guideline$)).mp.	37866
48	terminology/	0
49	(recruit$ adj4 (effective$ or model$ or pause$ or pausing)).mp. or recruit$.ti. or *Patient Selection/	50660
50	((decision$ adj2 algorithm$).mp.	2965
51	sponsor$.ti.	3863
52	fund$.ti.	43682
53	resourcing.mp.	1190
54	exp financial management/ or financing, organized/ or taxes/ or (financ$ not incentiv$).ti.	118701
55	economic$.ti.	57184
56	(trial$ adj1 cost$).mp. or (costs or costing).ti.	39270
57	contract research org$.mp.	428
58	(regulations or (regulat$ adj3 framework$)).mp.	60029
59	Government Regulation/	21840
60	jurisprudence/ or confidentiality/ or personally identifiable information/ or international law/ or legal services/ or liability, legal/ or mandatory reporting/ or legislation, drug/	80153
61	((legal$ or law or laws or legislat$ or policy or policies or rule or rules) adj5 (UK or United Kingdom or brit$ or EU or Europe$ or framework$ or national or government$)).mp.	50560
62	(clinical trial regulation or "536/2014").mp.	95
63	((EU or Europe or European) adj4 representative$).mp.	810
64	collaborat$.ti.	37346
65	(harmonis$ or harmoniz$).mp.	20656
66	brexit.mp.	771
67	(safety adj3 (monitor$ or governance)).mp. or Patient Safety/	31531
68	(((intervention or IMP or investigational medicin$ or drug$) adj6 (supply$ or inventory or export$ or import$)) or procur$).mp.	130269
69	(treatment selection or site selection).mp.	8819
70	workload$.mp.	51329
71	Contracts/ or (contract or contracts).mp.	37008
72	((fund$ or collaborat$ or delegat$ or site or sites or research or sponsor$) adj5 (agreement or agreements)).mp.	3553
73	((operational or practical or legal or administrative or financial or procedural or ethical or methodolog$ or statistical or recruitment or sponsor$ or logistic$ or design) adj4 (complexit$ or guidance or guidelines or recommendations or considerations or issues or challenges or obstacles or barriers or difficulties or advantages or disadvantages)).mp.	135867
74	exp *Clinical Trials as Topic/es, lj, og, st, sd [Ethics, Legislation & Jurisprudence, Organization & Administration, Supply & Distribution]	11020
75	or/38-74 [any of the various specific challenges]	1238686
76	(9 or 19) and 37 and 75	2818
77	(9 or 19) and (*Research Design/ or *Methods/)	1335
78	9 and 19 and (37 or 75)	72
79	76 or 77 or 78	3675
80	limit 79 to yr="2006 -Current"	3065
81	80 and (exp clinical trial/ or trial$.mp.)	2686

For peer review only

82	limit 81 to ("review" or "scientific integrity review" or "systematic review")	498
83	81 not (82 and (systematic and review).ti.)	2630
84	83 not (exp animals/ not humans.sh.)	2625
85	limit 84 to english language	2555

Database: EMBASE, 31 January 2023

1	adaptive clinical trial/	377
2	((adaptive or umbrella or basket or bucket) adj2 (design or trial or trials or study or studies or protocol$)).mp.	6949
3	(trial$ platform$ or platform design or platform trial$ or platform clinical trial$ or platform study or platform studies or platform protocol$).mp.	1567
4	((adaptive adj6 design$) and (trial or trials or study or studies or protocol$)).mp.	4842
5	(complex innovative adj3 (trial$ or design$ or protocol$)).mp.	29
6	master protocol$.mp.	439
7	response adaptive randomi$.mp.	207
8	(single centre or single site).mp.	66742
9	(or/1-7) not 8 [adaptive design - not single-site]	10465
10	(international adj5 (trial or trials or protocol$ or multicent$ or multi-cent$ or multisite or multi-site or multi-arm or multiarm or multi-stage or multistage)).mp.	32583
11	(international clinical$ trial$ regist$ or international clinical$ trial$ platform$ or international standard randomi$ control$ trial$ number$).mp.	4034
12	international.mp. /freq=2	165479
13	10 not (11 not 12) [gets rid of results with 'international trial' that only refer to the WHO ICTRP]	29033
14	((multinational or multi-national or intercontinental or inter-continental or pan-europ$) adj8 (trial or trials or protocol$ or multicent$ or multi-cent$ or multisite or multi-site)).mp.	5901
15	((trial or trials or protocol$) and (((multicent$ or multi-cent$ or multisite or multi-site) and sites) or centres or centers) and countries).mp.	9439
16	((UK or United Kingdom) and (EU or Europ$) and (trial$ or protocol$)).mp.	8673
17	(western europe/ or austria/ or exp belgium/ or benelux/ or exp france/ or germany/ or ireland/ or liechtenstein/ or luxembourg/ or monaco/ or netherlands/ or exp scandinavia/ or switzerland/ or exp Eastern Europe/ or exp Southern Europe/) and exp United Kingdom/ and (trial$ or protocol$).mp.	5785
18	((exp Eastern Europe/ and exp Southern Europe/) or (exp Eastern Europe/ and exp Western Europe/) or (exp Western Europe/ and exp Southern Europe/)) and (trial$ or protocol$).mp.	6768
19	(exp western hemisphere/ or exp africa/ or exp asia/ or exp "australia and new zealand"/) and exp Europe/ and (trial$ or protocol$).mp.	25734
20	or/13-19 [main international trial requirement]	77781
21	methodology/	1630438
22	study design/	54726
23	challenge$.mp.	1076548
24	(((trial$ or design) adj5 efficiency) or efficiencies).mp.	59423
25	((complexit$ or guidance or guidelines or recommendations or considerations or issues or obstacles or barriers) and trial$).ti.	6537
26	((lesson or lessons or pitfall or pitfalls) and (design$ or plan$)).mp.	35412
27	(trial$ adj5 (manag$ or run or running or conducting)).mp.	25255
28	(design$ or rationale$ or implement$ or methodol$).ti.	345887
29	(trial$ and (framework$ or regulations)).ti.	629
30	(trials and (Europ$ or EU or multinational or multi-national or intercontinental or inter-continental or international or multi-cent$ or multicent$ or multi-site or multisite)).ti.	4222
31	(trial$ adj1 (approval or authorisation or authorization or regulation)).mp.	579
32	management/ or joint venture/ or total quality management/ or work schedule/ or workflow/	183307
33	"multicenter study (topic)"/	38773
34	exp *"clinical trial (topic)"/	21048
35	(trial$ and design$).kf.	4092
36	(((good or best) adj3 practice$) and trial$).ti.	301

For peer review only

37	(trial$ adj2 (authoris$ or authoriz$)).mp.	223
38	or/21-37 [general study design/methodology terms]	3300880
39	((trial$ adj5 insur$) or insuring or indemni$).mp.	3566
40	((add$ or remov$) adj5 (arm or arms)).mp.	6264
41	gdpr.mp.	528
42	exp *ethics/ or ethic$.ti.	134708
43	((data adj8 secur$) or (data adj3 (share$ or sharing)) or data management or data governance).mp. or data protection/ or anonymization/ or data privacy/ or encryption/	37340
44	data quality/ or data accuracy/ or data availability/ or data completeness/ or data consistency/ or data validity/	8842
45	(trial$ adj3 infrastructure).mp.	316
46	*statistical model/ or *statistical analysis/ or ((statistical model/ or statistical analysis/) and ((statistical adj3 (model$ or framework$ or plan$)) or estimand$).ti,ab.) or statistical design/ or (statistical adj1 design$).mp.	55949
47	software/ or software design/ or general software/ or exp data analysis software/	203465
48	(reporting guideline$ or outcome reporting).kf. or reporting.ti. or (reporting adj4 (quality or guideline$)).mp.	48775
49	nomenclature/	60593
50	(recruit$ adj4 (effective$ or model$ or pause$ or pausing)).mp. or recruit$.ti. or *patient selection/	51069
51	(decision$ adj2 algorithm$).mp.	4194
52	sponsor$.ti.	4495
53	funding/ or fund$.ti.	114234
54	resourcing.mp.	1588
55	financial management/ or accounting/ or "billing and claims"/ or budget/ or finance/ or corporate finance/ or public finance/ or purchasing/ or exp tax/ or resource management/ or resource allocation/ or (financ$ not incentiv$).ti.	233201
56	economic evaluation/ or economic$.ti.	81922
57	(trial$ adj1 cost$).mp. or (costs or costing).ti.	52721
58	contract research org$.mp.	934
59	(regulations or (regulat$ adj3 framework$)).mp.	77414
60	*european medicines agency/ or *"medicines and healthcare products regulatory agency"/	604
61	legal aspect/ or government regulation/ or legal evidence/ or legal liability/ or legal procedure/ or legal service/ or medical liability/ or medicolegal aspect/ or jurisprudence/	281458
62	((legal$ or law or laws or legislat$ or policy or policies or rule or rules) adj5 (UK or United Kingdom or brit$ or EU or Europe$ or framework$ or national or government$)).mp.	68360
63	(clinical trial regulation or "536/2014").mp.	168
64	((EU or Europe or European) adj4 representative$).mp.	1071
65	collaborat$.ti.	45453
66	(harmonis$ or harmoniz$).mp.	31555
67	brexit.mp.	929
68	(safety adj3 (monitor$ or governance)).mp.	11693
69	(((intervention or IMP or investigational medicin$ or drug$) adj6 (supply$ or inventory or export$ or import$)) or procur$).mp.	114343
70	(treatment selection or site selection).mp.	12860
71	workload$.mp.	72251
72	contract/ or (contract or contracts).mp.	38623
73	((fund$ or collaborat$ or delegat$ or site or sites or research or sponsor$) adj5 (agreement or agreements)).mp.	5107
74	((operational or practical or legal or administrative or financial or procedural or ethical or methodolog$ or statistical or recruitment or sponsor$ or logistic$ or design) adj4 (complexit$ or guidance or guidelines or recommendations or considerations or issues or challenges or obstacles or barriers or difficulties or advantages or disadvantages)).mp.	168395
75	or/39-74 [any of the various specific challenges]	1787225
76	(9 or 20) and 38 and 75	4552
77	(9 or 20) and (*study design/ or *methodology/)	932
78	9 and 20 and (38 or 75)	192

For peer review only

79	76 or 77 or 78	5246
80	limit 79 to conference abstract	1834
81	79 not 80	3412
82	limit 81 to yr="2006 -Current"	3004
83	82 and (trial$.mp. or clinical research/ or exp clinical trial/)	2633
84	limit 83 to ("systematic review" or "review")	656
85	83 not (84 and (systematic and review).ti.)	2567
86	85 not ((exp animal/ or animal experiment/ or nonhuman/) not (exp human/ or human experiment/))	2557
87	limit 86 to english language	2478

Database: Health Management Information Consortium, 31 January 2023

1	((adaptive or umbrella or basket or bucket) adj2 (design or trial or trials or study or studies or protocol$)).mp.	15
2	(trial$ platform$ or platform design or platform trial$ or platform clinical trial$ or platform study or platform studies or platform protocol$).mp.	4
3	((adaptive adj6 design$) and (trial or trials or study or studies or protocol$)).mp.	8
4	(complex innovative adj3 (trial$ or design$ or protocol$)).mp.	0
5	master protocol$.mp.	1
6	response adaptive randomi$.mp.	0
7	(single centre or single site).mp.	129
8	(or/1-6) not 7 [adaptive design - not single-site]	22
9	(international adj5 (trial or trials or protocol$ or multicent$ or multi-cent$ or multisite or multi-site or multi-arm or multiarm or multi-stage or multistage)).mp.	114
10	(international clinical$ trial$ regist$ or international clinical$ trial$ platform$ or international standard randomi$ control$ trial$ number$).mp.	29
11	international.mp. /freq=2	3011
12	9 not (10 not 11) [gets rid of results with 'international trial' that only refer to the WHO ICTRP]	90
13	((multinational or multi-national or intercontinental or inter-continental or pan-europ$) adj8 (trial or trials or protocol$ or multicent$ or multi-cent$ or multisite or multi-site)).mp.	17
14	((trial or trials or protocol$) and (((multicent$ or multi-cent$ or multisite or multi-site) and sites) or centres or centers) and countries).mp.	26
15	((UK or United Kingdom) and (EU or Europ$) and (trial$ or protocol$)).mp.	113
16	(europe/ or albania/ or alps/ or andorra/ or austria/ or balkans/ or baltic countries/ or belarus/ or belgium/ or bosnia herzegovina/ or bulgaria/ or caucasus/ or central europe/ or croatia/ or cyprus/ or czechoslovakia/ or danube river/ or eastern europe/ or france/ or fyr macedonia/ or germany/ or gibraltar/ or greece/ or hungary/ or iberian peninsula/ or italy/ or liechtenstein/ or luxembourg/ or malta/ or mediterranean/ or moldova/ or monaco/ or montenegro/ or netherlands/ or papal states/ or poland/ or "republic of ireland"/ or romania/ or russia/ or san marino/ or scandinavia/ or serbia/ or slovenia/ or soviet union/ or switzerland/ or turkey/ or ukraine/ or yugoslavia/) and united kingdom/ and (trial$ or protocol$).mp.	22
17	(exp africa/ or exp asia/ or exp middle east/ or exp americas/ or exp oceania/) and exp europe/ and (trial$ or protocol$).mp.	64
18	or/12-17 [main international trial requirement]	303
19	methodology/	153
20	exp research strategies/	16557
21	challenge$.mp.	14704
22	(((trial$ or design) adj5 efficiency) or efficiencies).mp.	270
23	((complexit$ or guidance or guidelines or recommendations or considerations or issues or obstacles or barriers) and trial$).ti.	87
24	((lesson or lessons or pitfall or pitfalls) and (design$ or plan$)).mp.	1351
25	(trial$ adj4 (manag$ or run or running or conducting)).mp.	208
26	(design$ or rationale$ or implement$ or methodol$).ti.	7553
27	management/ or business management/ or corporate management/ or development management/ or joint management/ or office management/ or operational management/ or process management/ or programme management/ or project	9141

	management/ or quality management/ or research management/ or strategic management/ or team management/ or work organisation/ or administration/ or leadership/ or management communication/ or management operations/ or management planning/ or management practice/ or management process/ or management techniques/	
28	contract management/ or facilities management/ or financial management/ or human resources management/ or information management/ or knowledge management/ or materials & supplies management/ or physical distribution management/ or resource management/ or risk management/	10733
29	(((good or best) adj3 practice$) and trial$).ti.	13
30	(trial$ adj2 (authoris$ or authoriz$)).mp.	1
31	or/19-30	55720
32	((trial$ adj5 insur$) or insuring or indemni$).mp.	171
33	((add$ or remov$) adj5 (arm or arms)).mp.	7
34	gdpr.mp.	16
35	exp ethics/ or ethic$.ti.	4861
36	ethics committees/	135
37	((data adj8 secur$) or (data adj3 (share$ or sharing)) or data management or data governance).mp.	839
38	(trial$ adj3 infrastructure).mp.	1
39	statistical model/ or statistical analysis/ or statistical design/ or (statistical adj1 design$).mp.	551
40	software.mp.	1768
41	reporting.ti. or (reporting adj4 (quality or guideline$)).mp.	1107
42	(recruit$ adj4 (effective$ or model$ or pause$ or pausing)).mp. or recruit$.ti.	1058
43	(decision$ adj2 algorithm$).mp.	10
44	sponsor$.ti.	190
45	fund$.ti.	5155
46	exp financing/ or (financ$ not incentiv$).ti.	16452
47	resourcing.mp.	358
48	economic evaluation/ or economic.ti.	3423
49	(trial$ adj1 cost$).mp. or (costs or costing).ti.	3347
50	contract research org$.mp.	4
51	(regulations or (regulat$ adj3 framework$)).mp. or drug regulations/ or regulations/	6548
52	((legal$ or law or laws or legislat$ or policy or policies or rule or rules) adj5 (UK or United Kingdom or brit$ or EU or Europe$ or framework$ or national or government$)).mp.	14895
53	(clinical trial regulation or "536/2014").mp.	3
54	((EU or Europe or European) adj4 representative$).mp.	25
55	collaborat$.ti.	1607
56	(harmonis$ or harmoniz$).mp.	377
57	brexit.mp.	193
58	(safety adj3 (monitor$ or governance)).mp. or drug safety/	191
59	(((intervention or IMP or investigational medicin$ or drug$) adj6 (supply$ or inventory or export$ or import$)) or procur$).mp.	2378
60	(treatment selection or site selection).mp.	27
61	workload$.mp.	3445
62	exp contracts/ or (contract or contracts).mp.	7573
63	((fund$ or collaborat$ or delegat$ or site or sites or research or sponsor$) adj5 (agreement or agreements)).mp.	112
64	((operational or practical or legal or administrative or financial or procedural or ethical or methodolog$ or statistical or recruitment or sponsor$ or logistic$ or design) adj4 (complexit$ or guidance or guidelines or recommendations or considerations or issues or challenges or obstacles or barriers or difficulties or advantages or disadvantages)).mp.	5882
65	or/32-64	71491
66	(8 or 18) and (31 or 65)	186
67	limit 66 to yr="2006 -Current"	110
68	limit 67 to english	110
69	68 not systematic review.ti.	92

For peer review only

2 DATA EXTRACTION TEMPLATE

Data was extracted on the elements listed in Table S1 for all identified studies.

Table S1. Data extracted from included studies

- First author
- Publication year
- Study type
- Trial acronym
- Trial phase
- Blinding status
- Trial status
- Start date
- End date
- Number and location of sites
- Total sample size
- What condition was the study on?
- Type of intervention
- Number of arms and interventions in each arm
- What is the primary outcome?

Sponsorship

- Type of sponsor
- Was it the same sponsor across all sites?
- Details of additional sponsorship

Funding

- Main funding source
- Details of what was funded
- Did they fund all sites?
- Any funding challenges or solutions?
- Insurance details
- Was there a standard operating procedure?

Regulatory

- Were any guidelines used in oversight, if so what were they?
- Was there an EU representative?
- Details of GDPR
- Details of trial monitoring and oversight
- Details of auditing
- Were site specific documents prepared?

Trial management

- Name of randomisation system
- Process of adding and removing arms
- Process of adding and removing sites
- How were interventions chosen?
- Was there a trial steering committee present?
- Recruitment challenges and solutions
- Details of protocol amendments
- Staff training
- Name of data management system
- Any reported data management issues?

Intervention

- Who supplied the intervention?
- Was it the same suppler across all sites?
- Details of procurement and distribution
- Were pharmacies/pharmacists involved?
- How were adverse events monitored?
- Details of intervention licensing

Biospecimens

- What specimens were collected?
- Were they collected at all sites?
- Where were specimens processed?
- Details of specimen transport between sites

Agreements

- Was a data sharing agreement present?
- Who was the agreement between?
- Were site agreements present?
- Details of agreement challenges and solutions
- Who sought ethics approval?
- Details of ethics approval
- What was the contractual responsibility of site leads and stakeholders?
- Were materials translated?
- Details of translation and communication between sites

For peer review only

3 SUMMARY OF INCLUDED STUDIES

Table S2. Summary of included studies

First author (year)	Trial name	Trial design	Population	Number of sites	Site locations	Main coordinating centre(s)	Total participants	Intervention	Primary outcome	Enrolment period
Aban (2008)[25]	MGTX	Parallel	Myasthenia gravis	79	Global	USA	126	Other	Effectiveness	June 2006 – December 2015
Aitken (2008)[32]	PROMOTION	Parallel	Coronary artery disease	5	North America, Oceania	USA	3522	Behavioural	Prevention	February 2001 – June 2006
Angus (2020)[33]	REMAP-CAP	Adaptive-platform	Severe pneumonia and COVID-19		UK/EU, North America, Oceania, Asia	Australia, Thailand		Drug	Effectiveness	April 2016 – present
Aryal (2021)[38]	REMAP-CAP	Adaptive-platform	Severe pneumonia and COVID-19		UK/EU, North America, Oceania, Asia	RCC in Australia and Thailand		Drug	Effectiveness	April 2016 – present
Antic (2015)[42]	SAVE	Parallel	Obstructive sleep apnoea	89	Oceania, North America, South America, Asia, UK/EU	RCC in Australia, Brazil, China, India and Spain	2717	Device	Prevention	September 2008 – December 2015
Babiker (2013)[18]	START	Parallel	HIV	237	North America, South America, UK/EU, Oceania, Africa	RCC in Denmark, UK, Australia, USA	4000	Drug	Efficacy	April 2009 – July 2022
Berthon-Jones (2015)[26]	ALTAIR	Parallel	HIV	36	Asia, Oceania, UK/EU, North America, South America		322	Drug	Effectiveness	February 2007 – November 2011
Bryant (2021)[49]	TBTC Study 31	Parallel	Tuberculosis	34	North America, South America, Asia, Africa	USA	2516	Drug	Effectiveness	January 2016 – May 2021
Carli (2013)[43]	SEYLE	Cluster	Suicide	11	UK/EU	Sweden	11110	Behavioural	Prevention	September 2009 – January 2012
Clasen (2020)[53]	HAPIN	Parallel	Low birth weight		Asia, North America, South America, Africa	USA		Device	Prevention	September 2017 – present
Coomarasamy (2016)[50]	PROMISE	Parallel	Recurrent miscarriage	45	UK/EU	UK	836	Drug	Efficacy	June 2008 – May 2012
Crow (2018)[19]	FOR-DMD	Parallel	Duchenne muscular dystrophy	40	North America, UK/EU	USA	196	Drug	Effectiveness	January 2013 – November 2019
del Álamo (2022)[20]										
Denholm (2022)[51]	ASCOT ADAPT	Adaptive-platform	COVID-19		Oceania, Asia			Drug	Effectiveness	February 2021 – present
Dutton (2009)[44]	CONTROL	Parallel	Trauma	75	North America, South America, UK/EU, Asia, Africa	USA	576	Drug	Efficacy	October 2005 – September 2008
Eikelboom (2022)[39]	ACT	Factorial	COVID-19	62	North America, South America, Africa, Asia		6528	Drug	Effectiveness	April 2020 – February 2022
Fogelholm (2017)[45]	PREVIEW	Factorial	Pre-diabetes	8	UK/EU, Oceania		2326	Behavioural	Prevention	June 2013 – December 2018
Franciscus (2014)[52]	TRIGR	Parallel	Type 1 diabetes	77	North America, Oceania, UK/EU	USA	5156	Other	Prevention	May 2002 – December 2006
Fulda (2023)[48]	REPRIEVE	Parallel	HIV		North America, South America, Africa, Asia, UK/EU	USA		Drug	Prevention	March 2015 – present
Goossens (2021)[27]	REMAP-CAP	Adaptive-platform	Severe pneumonia and COVID-19		UK/EU, North America, Oceania, Asia	Australia, Thailand		Drug	Effectiveness	April 2016 – present

Grarup (2015)[31]	START	Parallel	HIV	237	North America, South America, UK/EU, Oceania, Africa	RCC in Denmark, UK, Australia, USA	4000	Drug	Efficacy	April 2009 – July 2022
Hata (2020)[40]	PATHWAY	Parallel	Breast cancer	23	Asia		185	Drug	Efficacy	February 2018 – July 2022
Herrick (2012)[34]	FDTT	Parallel	Functional dyspepsia	8	North America		292	Drug	Efficacy	October 2006 – July 2013
Jeon (2016)[46]	CLEAR III	Parallel	Intracerebral haemorrhage	73	North America, South America, UK/EU, Asia	USA	500	Drug	Efficacy	September 2009 – January 2015
Kenyon (2011)[21]	STICH II	Parallel	Intracerebral haemorrhage	126	North America, Oceania, UK/EU, Asia, Africa	UK	601	Other	Efficacy	January 2007 – August 2015
Kesho Bora Study Group (2011)[35]	Kesho-Bora	Parallel	HIV	5	Africa	Switzerland	824	Drug	Efficacy	June 2005 – August 2008
Kolitsopoulos (2013)[47]	ZODIAC	Parallel	Schizophrenia	226	North America, South America, UK/EU, Asia		18240	Drug	Effectiveness	February 2002 – April 2007
Larson (2016)[22]	INSIGHT trials					RCC in UK, Denmark, USA and Australia				
Lingor (2021)[23]	ROCK-ALS / ROCK-ALS-US	Parallel	Amyotrophic lateral sclerosis		North America, UK/EU			Drug	Efficacy	February 2019 – present
Minisman (2012)[5]	MGTX	Parallel	Myasthenia gravis	79	Global	USA	126	Other	Effectiveness	June 2006 – December 2015
Murray (2022)[24]	TICO	Adaptive-platform	COVID-19		North America, UK/EU, Asia, Africa	USA with 8 RCC		Drug	Effectiveness	August 2020 – present
Neaton (2010)[9]	INSIGHT trials					RCC in UK, Denmark, USA and Australia				
Ravinetto (2013)[36]	4ABC	Parallel	Malaria	12	Africa	Belgium	4112	Drug	Efficacy	July 2007 – December 2009
Reams (2018)[41]	DOVE	Parallel	Sickle cell disease	51	North America, South America, UK/EU, Asia, Africa		341	Drug	Efficacy	April 2013 – December 2015
Seal (2006)[28]		Factorial	Endophthalmitis	24	UK/EU, Asia	UK	35000	Drug	Efficacy	September 2003 – May 2006
Spencer (2012)[54]	AWARD-5	Adaptive	Type 2 diabetes	111	North America, UK/EU, Asia		1202	Drug	Safety	August 2008 – July 2012
Sydes (2011)[29]	STAMPEDE	Adaptive-platform	Prostate cancer		UK/EU	UK		Drug	Efficacy	July 2005 – present
Zimmer (2010)[37]	BAMSG 3-01	Parallel	Cryptococcal meningitis	13	North America, Asia	USA	143	Drug	Efficacy	May 2005 – April 2008

RCC: Regional Coordinating Centre; RCT: Randomised Controlled Trial.

For peer review only

4 OPERATIONAL COMPLEXITIES REPORTED BY PUBLICATION

Table S3. Operational complexities in conducting international trials

Major barriers	Source
**Study set-up**	
Sponsorship, insurance and need for EU legal representative	Aban (2008); Babiker (2013); Berthon-Jones (2015); Crow (2018); del Álamo (2022); Goossens (2021); Kenyon (2011); Lingor (2021); Minisman (2012); Murray (2022); Neaton (2010); Seal (2006); Sydes (2011)
Funding	Aban (2008); Aitken (2008); Angus (2020); Babiker (2013); Crow (2018); del Álamo (2022); Goossens (2021); Herrick (2012); Kenyon (2011); Kesho Bora Study Group (2011); Larson (2016); Lingor (2021); Ravinetto (2013); Zimmer (2010)
Lack of harmonisation in ethics and regulatory approvals	Aban (2008); Aitken (2008); Aryal (2021); Babiker (2013); Berthon-Jones (2015); del Álamo (2022); Eikelboom (2022); Goossens (2021); Grarup (2015); Hata (2020); Kenyon (2011); Minisman (2012); Murray (2022); Neaton (2010); Ravinetto (2013); Reams (2018); Zimmer (2010)
**Site set-up**	
Training	Aitken (2008); Antic (2015); Aryal (2021); Carli (2013); Crow (2018); Dutton (2009); Fogelholm (2017); Jeon (2016); Kesho Bora Study Group (2011); Kolitsopoulos (2013); Minisman (2012)
Contracts	Babiker (2013); Crow (2018); del Álamo (2022); Goossens (2021); Grarup (2015); Larson (2016); Lingor (2021); Minisman (2012)
Site monitoring	Angus (2020); Aryal (2021); Bryant (2021); Carli (2013); Coomarasamy (2016); Denholm (2022); Dutton (2009); Franciscus (2014); Fulda (2023); Larson (2016); Ravinetto (2013); Seal (2006); Sydes (2011); Zimmer (2010)
Communication	Aitken (2008); Angus (2020); Antic (2015); Crow (2018); Denholm (2022); Fulda (2023); Herrick (2012); Minisman (2012)
Translation of materials	Aban (2008); Babiker (2013); Berthon-Jones (2015); Carli (2013); Crow (2018); Franciscus (2014); Lingor (2021); Zimmer (2010)
**Trial management**	
Trial oversight	Antic (2015); Babiker (2013); Coomarasamy (2016); Dutton (2009); Franciscus (2014); Herrick (2012); Larson (2016); Murray (2022); Spencer (2012)
Site and intervention selection	Angus (2020); Antic (2015); Coomarasamy (2016); Denholm (2022); Dutton (2009); Eikelboom (2022); Fulda (2023); Herrick (2012); Kesho Bora Study Group (2011); Kolitsopoulos (2013); Minisman (2012); Murray (2022); Reams (2018); Zimmer (2010)
Recruitment	Aitken (2008); Antic (2015); Berthon-Jones (2015); Eikelboom (2022); Franciscus (2014); Herrick (2012); Kesho Bora Study Group (2011); Kolitsopoulos (2013); Reams (2018)
**Data management**	Aitken (2008); Aryal (2021); Berthon-Jones (2015); Carli (2013); Coomarasamy (2016); Ravinetto (2013); Seal (2006)
**Intervention management**	
Drug procurement and distribution	Babiker (2013); Bryant (2021); Coomarasamy (2016); Crow (2018); del Álamo (2022); Goossens (2021); Grarup (2015); Hata (2020); Herrick (2012); Jeon (2016); Lingor (2021); Minisman (2012); Murray (2022); Ravinetto (2013); Reams (2018); Seal (2006); Spencer (2012); Zimmer (2010)
Pharmacy involvement	Aban (2008); Bryant (2021); Coomarasamy (2016); Herrick (2012); Jeon (2016); Kolitsopoulos (2013); Minisman (2012)
Monitoring safety and adverse events	Antic (2015); Bryant (2021); Coomarasamy (2016); Hata (2020); Herrick (2012); Minisman (2012); Murray (2022); Zimmer (2010)
Biospecimen processing and transport	Berthon-Jones (2015); Bryant (2021); Denholm (2022); Fogelholm (2017); Franciscus (2014); Fulda (2023); Hata (2020); Lingor (2021)
**Adaptive-specific**	Angus (2020); Aryal (2021); Denholm (2022); Murray (2022); Spencer (2012)

For peer review only

5 PRISMA CHECKLIST

Section and Topic	Item #	Checklist item	Location where item is reported
**TITLE**			
Title	1	Identify the report as a systematic review.	Page 1
**ABSTRACT**			
Abstract	2	See the PRISMA 2020 for Abstracts checklist.	Page 2
**INTRODUCTION**			
Rationale	3	Describe the rationale for the review in the context of existing knowledge.	Page 4
Objectives	4	Provide an explicit statement of the objective(s) or question(s) the review addresses.	Page 4
**METHODS**			
Eligibility criteria	5	Specify the inclusion and exclusion criteria for the review and how studies were grouped for the syntheses.	Page 5
Information sources	6	Specify all databases, registers, websites, organisations, reference lists and other sources searched or consulted to identify studies. Specify the date when each source was last searched or consulted.	Page 5
Search strategy	7	Present the full search strategies for all databases, registers and websites, including any filters and limits used.	Page 5
Selection process	8	Specify the methods used to decide whether a study met the inclusion criteria of the review, including how many reviewers screened each record and each report retrieved, whether they worked independently, and if applicable, details of automation tools used in the process.	Page 5, Figure 1
Data collection process	9	Specify the methods used to collect data from reports, including how many reviewers collected data from each report, whether they worked independently, any processes for obtaining or confirming data from study investigators, and if applicable, details of automation tools used in the process.	Page 5-6
Data items	10a	List and define all outcomes for which data were sought. Specify whether all results that were compatible with each outcome domain in each study were sought (e.g. for all measures, time points, analyses), and if not, the methods used to decide which results to collect.	Page 6
	10b	List and define all other variables for which data were sought (e.g. participant and intervention characteristics, funding sources). Describe any assumptions made about any missing or unclear information.	Page 6
Study risk of bias assessment	11	Specify the methods used to assess risk of bias in the included studies, including details of the tool(s) used, how many reviewers assessed each study and whether they worked independently, and if applicable, details of automation tools used in the process.	NA
Effect measures	12	Specify for each outcome the effect measure(s) (e.g. risk ratio, mean difference) used in the synthesis or presentation of results.	Page 5-6
Synthesis methods	13a	Describe the processes used to decide which studies were eligible for each synthesis (e.g. tabulating the study intervention characteristics and comparing against the planned groups for each synthesis (item #5)).	Page 5-6
	13b	Describe any methods required to prepare the data for presentation or synthesis, such as handling of missing summary statistics, or data conversions.	NA
	13c	Describe any methods used to tabulate or visually display results of individual studies and syntheses.	NA
	13d	Describe any methods used to synthesize results and provide a rationale for the choice(s). If meta-analysis was performed, describe the model(s), method(s) to identify the presence and extent of statistical heterogeneity, and software package(s) used.	Page 5-6
	13e	Describe any methods used to explore possible causes of heterogeneity among study results (e.g. subgroup analysis, meta-regression).	NA
	13f	Describe any sensitivity analyses conducted to assess robustness of the synthesized results.	NA
Reporting bias assessment	14	Describe any methods used to assess risk of bias due to missing results in a synthesis (arising from reporting biases).	NA
Certainty assessment	15	Describe any methods used to assess certainty (or confidence) in the body of evidence for an outcome.	NA
**RESULTS**			
Study selection	16a	Describe the results of the search and selection process, from the number of records identified in the search to the number of studies included in the review, ideally using a flow diagram.	Page 6-7
	16b	Cite studies that might appear to meet the inclusion criteria, but which were excluded, and explain why they were excluded.	Page 6-7 / Figure 1
Study characteristics	17	Cite each included study and present its characteristics.	Page 6-7 / Table 1
Risk of bias in studies	18	Present assessments of risk of bias for each included study.	NA
Results of individual studies	19	For all outcomes, present, for each study: (a) summary statistics for each group (where appropriate) and (b) an effect estimate and its precision (e.g. confidence/credible interval), ideally using structured tables or plots.	Table 2, Page 6-16

For peer review only

Section and Topic	Item #	Checklist item	Location where item is reported
Results of syntheses	20a	For each synthesis, briefly summarise the characteristics and risk of bias among contributing studies.	NA
	20b	Present results of all statistical syntheses conducted. If meta-analysis was done, present for each the summary estimate and its precision (e.g. confidence/credible interval) and measures of statistical heterogeneity. If comparing groups, describe the direction of the effect.	NA
	20c	Present results of all investigations of possible causes of heterogeneity among study results.	NA
	20d	Present results of all sensitivity analyses conducted to assess the robustness of the synthesized results.	NA
Reporting biases	21	Present assessments of risk of bias due to missing results (arising from reporting biases) for each synthesis assessed.	NA
Certainty of evidence	22	Present assessments of certainty (or confidence) in the body of evidence for each outcome assessed.	NA
**DISCUSSION**			
Discussion	23a	Provide a general interpretation of the results in the context of other evidence.	Page 13-15
	23b	Discuss any limitations of the evidence included in the review.	Page 15
	23c	Discuss any limitations of the review processes used.	Page 15
	23d	Discuss implications of the results for practice, policy, and future research.	Page 13-15
**OTHER INFORMATION**			
Registration and protocol	24a	Provide registration information for the review, including register name and registration number, or state that the review was not registered.	Page 5
	24b	Indicate where the review protocol can be accessed, or state that a protocol was not prepared.	NA
	24c	Describe and explain any amendments to information provided at registration or in the protocol.	NA
Support	25	Describe sources of financial or non-financial support for the review, and the role of the funders or sponsors in the review.	Page 3
Competing interests	26	Declare any competing interests of review authors.	Page 3
Availability of data, code and other materials	27	Report which of the following are publicly available and where they can be found: template data collection forms; data extracted from included studies; data used for all analyses; analytic code; any other materials used in the review.	

For peer review only

6 PROTOCOL

Operational best practice in international clinical trials: a systematic review protocol

Support and registration:
This systematic review has been funded by the NIHR (Application Accelerator Award reference number: NIHR153955). The funder will have no further role in any aspect of the review. This protocol has been registered with the Open Science Framework (registration DOI: https://doi.org/10.17605/OSF.IO/YVTJB) (1).

Aim:
The aim of this systematic review is to identify models of best practice and lessons learned from the set up and delivery of international clinical trials, specifically in regard to management of cross-border regulatory and logistical requirements.

Rationale:
Research practices have transformed over the last decade, evolving from large single centred trials to more global trials that promote international collaboration. International trials offer numerous advantages over single-centre including reduced operational costs particularly in developing countries, faster recruitment and expand the horizon for the availability of treatments. Despite their benefits, the process of expanding to multiple sites raises logistical challenges due to the diversity in laws, ethics, guidelines and regulations between countries.

Search strategy:
The search strategy will be designed in collaboration with an experienced information specialist and will be peer-reviewed by another information specialist using the PRESS checklist. Thesaurus headings and keywords have been used as appropriate, and the search will be translated to other sources accordingly. The search strategy will cover four areas: "international trials", "adaptive design trials", "study design", and "specific challenges" and requirements of designing and running trials. Multiple approaches to combining these aspects will be used to achieve a practical quantity of specific, relevant results, while also mitigating risks of missing relevant material. A wide range of subject headings and search terms in appropriate fields will be used for all concepts in the search, including proximity searching to cover the many variations on phrases applicable to these topics. We will also apply a date filter to include studies published after 2005 as international trials have become prominent only in the last 15-20 years. We will search Medline, Embase and Health Management Information Consortium (HMIC) for appropriate studies.

Participants or population:
As the focus is on the implementation and infrastructure of the trial, any clinical population and any condition will be considered of interest.

Intervention:
Clinical trials on any drug, device or therapeutic intervention will be considered if they are multi-centre and trial site locations are in more than one country.

Comparator:
Trials will be eligible regardless of comparator.

Study designs to be included:
Any multi-centre randomised trial design with sites in at least two different countries will be included.

Eligibility criteria:
The following types of studies will be included:

1. Any study reporting operational challenges to conducting international trials (involving two or more countries)
2. Available in English
3. Published after 2005

Conference abstracts, systematic reviews and protocols will be excluded.

Main outcome:
Any outcomes related to the best practice models and lessons learned in aspects of set up and delivery of international trials.

We will specifically look at:

- Sponsorship and funding: balancing UK, EU and other international trial regulations, policies and procedures in sponsorship of the overall trial
- Trial management structure: legal, ethical and regulatory matters, safety and monitoring standards, processes for adding and/or removing trial arms, GDPR and clinical data management system requirements
- Intervention management: pharmacy involvement, procurement, distribution, delivery of interventions

For peer review only - http://bmjopen.bmj.com/site/about/guidelines.xhtml

- Contracting: agreements of collaboration, sites, delegation, sample transfer logistics arrangements and data sharing.

Data management:
Zotero will be used to manage the studies throughout the review. Rayyan will be used to screen records.

Strategy for data synthesis:
Two reviewers will independently screen the titles and abstracts of the studies retrieved by the search. For studies deemed eligible, or studies where it is impossible to decide eligibility from the abstract, the full text will be retrieved, and two reviewers will independently assess for inclusion. Any disagreements will be resolved through discussion or if necessary, by reference to a third reviewer.

Data extraction will be undertaken by one reviewer and checked by a second, with discrepancies resolved by consultation with a third. Where studies are reported in multiple publications, we will extract relevant data from all publications but consider as one study. Where data is missing or unclear, we will contact authors to request details or clarification. The following data will be extracted from included studies:

- Citation information
- Study design variables: sample size, phase
- Study population and objective variables: age, condition, experimental interventions, primary outcome
- Regulatory information: status, location, availability of results, start and completion date
- Outcome data: Sponsorship information; funding information; trial management process information; intervention/pharmacy process information; detail on the contracting processes

In the first instance, we will present a summary of study characteristics and outcome data in a series of structured tables to give a clear picture of the available evidence.

Language:
Only studies published in English will be included.

References

1. Gumber L, Pratt A, Bardgett M, Inskip A, Still M, Phillipson J, et al. Operational best practice in international clinical trials: a systematic review protocol 2023. osf.io/yvtjb.

For peer review only

BMJ Open

Operational complexities in international clinical trials: a systematic review of challenges and proposed solutions

Journal:	*BMJ Open*
Manuscript ID	bmjopen-2023-077132.R2
Article Type:	Original research
Date Submitted by the Author:	26-Feb-2024
Complete List of Authors:	Gumber, Leher; Newcastle University, Translational and Clinical Research Institute; Northumbria Healthcare NHS Foundation Trust,<br>Agbeleye, Opeyemi; Newcastle University, Population Health Sciences Institute<br>Inskip, Alex; Newcastle University, Population Health Sciences Institute<br>Fairbairn, Ross; Newcastle University, Population Health Sciences Institute<br>Still, Madeleine; Newcastle University<br>Ouma, Luke; Newcastle University, Population Health Sciences Institute<br>Lozano-Kuehne, Jingky; Newcastle University, Population Health Sciences Institute<br>Bardgett, Michelle; Newcastle University<br>Isaacs, John; Newcastle University, Translational and Experimental Medicine Institute; Newcastle Upon Tyne Hospitals NHS Foundation Trust, Musculoskeletal Unit<br>Wason, James; Newcastle University, Population Health Sciences Institute; University of Cambridge, MRC Biostatistics Unit<br>Craig, Dawn; Newcastle University, Health Economics Group, Institute of Health and Society<br>Pratt, Arthur; Newcastle University, Translational and Clinical Research Institute; Newcastle Upon Tyne Hospitals NHS Foundation Trust, Musculoskeletal Unit
**Primary Subject Heading**:	Research methods
Secondary Subject Heading:	Research methods, Global health
Keywords:	Clinical Trial, Randomized Controlled Trial, Systematic Review

SCHOLARONE™
Manuscripts

I, the Submitting Author has the right to grant and does grant on behalf of all authors of the Work (as defined in the below author licence), an exclusive licence and/or a non-exclusive licence for contributions from authors who are: i) UK Crown employees; ii) where BMJ has agreed a CC-BY licence shall apply, and/or iii) in accordance with the terms applicable for US Federal Government officers or employees acting as part of their official duties; on a worldwide, perpetual, irrevocable, royalty-free basis to BMJ Publishing Group Ltd ("BMJ") its licensees and where the relevant Journal is co-owned by BMJ to the co-owners of the Journal, to publish the Work in this journal and any other BMJ products and to exploit all rights, as set out in our licence.

The Submitting Author accepts and understands that any supply made under these terms is made by BMJ to the Submitting Author unless you are acting as an employee on behalf of your employer or a postgraduate student of an affiliated institution which is paying any applicable article publishing charge ("APC") for Open Access articles. Where the Submitting Author wishes to make the Work available on an Open Access basis (and intends to pay the relevant APC), the terms of reuse of such Open Access shall be governed by a Creative Commons licence – details of these licences and which Creative Commons licence will apply to this Work are set out in our licence referred to above.

Other than as permitted in any relevant BMJ Author's Self Archiving Policies, I confirm this Work has not been accepted for publication elsewhere, is not being considered for publication elsewhere and does not duplicate material already published. I confirm all authors consent to publication of this Work and authorise the granting of this licence.

For peer review only

Operational complexities in international clinical trials: a systematic review of challenges and proposed solutions

Leher Gumber[1,2], Opeyemi Agbeleye[3], Alex Inskip[3], Ross Fairbairn[3], Madeleine Still[3], Luke Ouma[4], Jingky Lozano-Kuehne[4], Michelle Bardgett[5], John D Isaacs[1,6], James M. S. Wason[4], Dawn Craig[3*] and Arthur G Pratt[1,6*].

[1]Translational and Clinical Research Institute, Newcastle University, UK.
[2]Northumbria Healthcare NHS Foundation Trust, UK.
[3]NIHR Innovation Observatory, Population Health Sciences Institute, Newcastle University, UK.
[4]Population Health Sciences Institute, Newcastle University, UK
[5]Newcastle Clinical Trial Unit, Newcastle University, UK.
[6]Musculoskeletal Unit, Newcastle upon Tyne Hospitals NHS Trust, UK.

*Denotes equal contribution

Corresponding author:
Dr Arthur G Pratt
Senior Clinical Lecturer and Consultant Rheumatologist
Translational and Clinical Research Institute
Newcastle University
Newcastle upon Tyne
NE1 7RU, UK
Email: arthur.pratt@newcastle.ac.uk

For peer review only

Abstract

Objective: International trials can be challenging to operationalise due to incompatibilities between country-specific policies and infrastructures. The aim of this systematic review was to identify the operational complexities of conducting international trials and identify potential solutions for overcoming them.

Design: Systematic review.

Data sources: Medline, Embase and Health Management Information Consortium were searched from 2006 to 30th January 2023.

Eligibility criteria: All studies reporting operational challenges (e.g. site selection, trial management, intervention management, data management) of conducting international trials were included.

Data extraction and synthesis: Search results were independently screened by at least two reviewers and data were extracted into a proforma.

Results: 38 studies (35 RCTs, 2 reports and 1 qualitative study) fulfilled the inclusion criteria. The median sample size was 1202 (IQR 332 – 4056) and median number of sites was 40 (IQR 13 – 78). 88.6% of studies had an academic sponsor and 80% were funded through government sources. Operational complexities were particularly reported during trial set-up due to lack of harmonisation in regulatory approvals and in relation to sponsorship structure, with associated budgetary impacts. Additional challenges included site selection, staff training, lengthy contract negotiations, site monitoring, communication, trial oversight, recruitment, data management, drug procurement and distribution, pharmacy involvement and biospecimen processing and transport.

Conclusions: International collaborative trials are valuable in cases where recruitment may be difficult, diversifying participation and applicability. However, multiple operational and regulatory challenges are encountered when implementing a trial in multiple countries. Careful planning and communication between trials units and investigators, with an emphasis on establishing adequately resourced cross-border sponsorship structures and regulatory approvals, may help to overcome these barriers and realise the benefits of the approach.

Open Science Framework registration number: osf-registrations-yvtjb-v1.

Strengths and limitations:

- A robust search and screening strategy was utilised to maximise the identification of relevant studies.
- All abstracts were screened by at least two reviewers.
- The review focused on international trials so the findings may not be generalisable to multi-centre studies conducted within the same country.
- The possibility that some specific challenges encountered might be omitted cannot be entirely excluded, nor can an element of subjectivity when summarising potential solutions to those identified.

Word count: 4263

Funding: This study was funded by National Institute of Health and Care Research (NIHR153955). Infrastructural support was provided by the National Institute of Health and Care Research (NIHR) Newcastle Biomedical Research Centre. JMSW is funded by a NIHR Research Professorship (NIHR301614). LG is funded by a NIHR Academic Clinical Fellowship. The views expressed are those of the author(s) and not necessarily those of the NIHR or the Department of Health and Social Care.

For peer review only

Competing interests statement: None declared.

Contribution of authors: LG was involved in conception of the study, screening, data extraction, analysis and writing up the manuscript. OA was involved in screening and writing up the manuscript. AI was involved in developing search strategy and revising it critically for important intellectual content. RF, MS were involved in screening and revising it critically for important intellectual content. MB was involved in developing search strategy and revising it critically for important intellectual content. LO, JLK, JDI and JW reviewed the manuscript for important intellectual content. Together with DC, AGP conceived and oversaw the project, revising the manuscript for important intellectual content.

Acknowledgements: We would like to thank Chris Price, Andrew Johnston, Julia Philipson and Ruth Wood for providing insights into outcome measures.

For peer review only

Introduction

The development and deployment of international clinical trials that enrol participants from more than one nation or jurisdiction, continues to increase [1,2]. Motivated by advancements in technology, globalisation and insufficient accrual rates using traditional approaches[3], recent examples increasingly adopt master protocols that allow treatment arms to be added and dropped adaptively over time – so-called "platform" trials – and other innovative designs [4]. International trials offer numerous advantages over single-nation approaches by increasing access to potentially eligible participants, enabling faster recruitment and/or larger sample sizes whilst encouraging representation of diverse ethnic, biological and socio-cultural groups[3,5–7]. They promote best practice globally and expand horizons for treatment availability in countries that may not otherwise have access to particular interventions[7]. The reduced operational cost of running a trial in developing compared to developed countries may be an additional consideration [8]. They furthermore foster collaboration and development of closer relationships among academics globally[3,5,7,9,10].

The conduct of international trials nonetheless presents unique challenges. Their success requires adherence to local and international laws, regulations and ethical requirements, availability of Good Clinical Practice (GCP)-trained researchers, adequate infrastructure at clinical sites, and close monitoring and oversight across multiple jurisdictions[7,11,12]. Incompatibilities between country-specific policies and challenges in the trial setup were previously identified as factors that extend study timelines and inflating costs[13]. Other contributing factors including site selection, insurance, logistics, regulatory requirements and sponsorship have been highlighted in a narrative review [14]. Platform trials present further complexity, potentially involving adaptations that incur ethical and/or regulatory review, sample size re-estimations, site capacity and data management challenges[15,16]. In recognition of these issues, we conducted a systematic review that aimed to identify: (1) the operational challenges of conducting international clinical trials and (2) potential practical solutions for overcoming them. Our overarching objective was to deliver a reference of practical value to prospective trialists planning to set up international trials in the modern era, with the potential to inform best practice guidelines.

Methods

For peer review only

This study was reported in accordance with the Preferred Reporting Items for Systematic Reviews and Meta-analyses (PRISMA) guidelines[17]. The study was registered with the Open Science Framework (Identifier: osf-registrations-yvtjb-v1)[18] and funded by National Institute of Health and Care Research (NIHR153955).

Search strategy and study selection

A search strategy was developed in collaboration with an information specialist (AI) using a combination of key words and MeSH terms. Medline and Embase were the primary bibliographic database sources, but a variation of the search was also run on the Health Management Information Consortium (HMIC) database. The search covered four broad concepts: (i) international trials, (ii) adaptive trials (in recognition that such trials frequently adopt international enrolment strategies not explicit in titles/abstracts), (iii) specific challenges to conducting trials and (iv) a focus on study design methods. These concepts were utilised in several different combinations to achieve a practicable quantity of relevant results, whilst mitigating the risk of omitting relevant material. Studies conducted prior to 2006 were considered less likely to reflect current legislation and hence a limit was applied to exclude them in the initial search strategy, as were systematic reviews and studies not available in English. A full description of the search strategy is provided in the supplement.

After de-duplication of records, titles and abstracts were rigorously double screened by at least two reviewers. The following studies were excluded: protocols, abstracts, multi-centre trials in a single country, studies published before 2006, studies not in English, systematic reviews and studies which did not report operational challenges. Operational challenges broadly encompassed issues related to approvals, opening sites, recruitment, and data management, and are further detailed in the supplement.

For studies deemed eligible, or where it was deemed impossible to decide eligibility from the abstract, the full text was retrieved. Full-text articles were screened for inclusion by one reviewer and 20% of the full-text articles underwent screening by a second reviewer. In case of discrepancy, the decision was taken by consensus of all the reviewers. Rayyan (Qatar Computing Research Institute), a systematic review web-based application, was used for screening, with record management facilitated throughout using the Zotero reference management tool.

Data extraction

For peer review only

Data were extracted by one reviewer using a pre-designed proforma (supplementary Table S1) and extractions were checked by a second reviewer. Aside from general trial characteristics, data extraction was under the broad headings: sponsorship; funding; regulatory considerations; trial management; intervention; biospecimens; agreements. Due to the nature of the review, a formal quality assessment was not performed.

Definition of variables and data analysis

The sponsor was determined as either academic (universities, hospitals or the government), or industry (pharmaceutical or device companies). The funder was defined as academic (university or hospital), government, industry or charity; where studies were co-funded by more than one source, the predominant funder was reported. Location of studies was categorised by continent into: UK/Europe (EU), Asia, Africa, North America, South America or Oceania. Primary outcome measures were considered as efficacy, effectiveness, prevention or safety. Quantitative data were described using numbers, percentages, median and interquartile range. Qualitative data were summarised into overarching themes which described the main challenges of conducting international trials. In relation to specific challenges identified during data extractions, solutions given by the authors were also recorded where available in each case and synthesised for reporting purposes.

Patient and public involvement

No patients were involved.

Results

The search identified 5215 records, of which 1588 were duplicates and removed. After title and abstract screening of the remaining 3627 articles, a further 3374 were excluded. Full text screening was conducted on 253 articles from which 38 were included (Figure 1). Characteristics of included studies are summarised in Table 1 and further details are available in supplementary Table S2.

Study characteristics

For peer review only

Of the 38 included studies, 35 (92.1%) were RCTs, 2 (5.3%) were reports and 1 (2.6%) was a qualitative study. Of the 35 RCTs, 24 (68.6%) had a parallel design, 7 (20.0%) were adaptive, 3 (8.6%) were factorial and 1 (2.9%) was cluster. Over half of the RCTs were open-label. Most RCTs evaluated a drug intervention (74.3%) and measured efficacy (40.0%) as their primary outcome. The median number of sites and sample size among closed trials was 40 (IQR 13 – 78) and 1202 (IQR 332 – 4056) respectively. The majority of studies (n=31, 88.6%) were sponsored by an academic institution and the most common funding source was government (80.0%). The continents most commonly represented by sites included in studies were North America (n=27, 77.1%), and UK/EU (n=25, 71.4%). Design characteristics of the included studies are summarised in Table 2. Operational complexities of conducting international trials were reported broadly at six stages of the trial process including study set-up, site set-up, trial management, data management, intervention management and adaptive specific features (Figure 2; supplementary Table S3). We structure our findings accordingly herein, summarising recurring themes and potential approaches arising to address them in Table 3.

Study set-up

Sponsorship, insurance and need for EU legal representative

Thirteen studies described variations in sponsorship and insurance requirements between countries which created delays[11,19–31]. This was particularly notable for UK/EU sites. The EU Clinical Trials Directive which governs the conduct of clinical trials in EU, requires the presence of an EU legal representative for trials sponsored by a non-EU institution[32]. After identification of an EU legal representative, repeated negotiations were necessary to clarify roles and responsibilities between the sponsor and legal representative resulting in further delays[19–21,33]. Country-specific variations in insurance and indemnity requirements within the EU were a further hurdle. For example, two studies noted that the minimum compensation in Germany was 500,000 euros and the insurance provided by the sponsor did not meet these limits. Additional insurance coverage could not be provided locally and, consequently, those sites were either dropped or authors had to run two parallel trials in different continents[23,24].

Funding

Fourteen studies described funding as a challenge[19–24,26,28,34–39]. Interestingly, all of these were funded through government sources. Limitations in funding meant that some investigators had to seek additional sources of

support and in cases where this was unsuccessful, there was a delay in trial start up[20,21,37,38]. Additionally, trials funded by the US had a requirement for non-US sites to obtain departmental clearance and approval from their respective countries before funds could be transferred. A lack of familiarity with this process prevented timely transfer of funds and thus delayed recruitment[26]. Predicting accurate budget projections and variations in currency exchange rates were additional challenges for trials spanning several years[34]. Prolonged negotiations between the funder, site and sponsor regarding site set-up costs led to further delays[33].

Lack of harmonisation in ethics and regulatory approvals

A lack of harmonisation in international legal and ethical systems was a reoccurring theme[11,19,21,22,25–29,33,34,38–43]. The time from initiation of regulatory procedures to the start of the trial ranged from 3 to 18 months. Hurdles included: lack of a centralised system and therefore the requirement for single-centre approvals[7,19,22,24,26,34,41], country-specific differences in requirements[7,22,24,26,38,42,43], infrequent ethics committee meetings[22,35], ethics review fee[22,38], translation of essential documents[42], protocol amendments[37,38] and lack of familiarity among ethics committees with trial design or conduct[22,41]. Additionally, multinational trials funded in total or in part by US National Institute of Health required Federalwide Assurance (FWA) approvals and annual reviews alongside adherence to country-specific regulations. Although this process was familiar to US sites, non-US sites found this challenging[7,11,23,26].

Site set-up

Training

In general, staff training was not considered a challenge[20,29,34,37,40,44–49]. Studies used a combination of in-person and online workshops which covered a broad range of topics including: study protocol, Good Clinical Practice, data management, intervention delivery and safety reporting[20,26,35,47,48]. One trial adopted a system of "train-the-trainer," whereby site leads would receive centralised training then train all personnel at their local sites[45].

Contracts

Contracts were formed at many levels including site agreements, funding agreements, sponsorship agreements, agreements with pharmaceutical companies and data sharing. Several studies described contract negotiations as a time consuming and lengthy process[19–21,23,24,28,29,33]. Contributing factors to this were: translation of

For peer review only

contracts[26], conflict with country-specific legal terminology and interpretation[20,21], clauses of indemnification[19,20] and administrative bureaucracy in legal departments[33]. Data sharing across borders requires additional levels of consent and protection. Trials with EU sites described how all participating sites were required to comply with the EU General Data Protection Regulations (GDPR)[21,24]. This required additional administrative steps[24]. To overcome these challenges, INSIGHT (International Network for Strategic Initiatives in Global HIV Trials) established coordinating centres in several countries that were geographically closer to sites, familiar with local regulatory requirements and fluent in local languages which improved the contract negotiation process[23].

Site monitoring

Monitoring and auditing of sites was conducted periodically and included checking for compliance with the protocol and standard operating procedures (SOPs), data consistency and missing data. Most studies adopted a combination of remote and in-person quality assurance visits and created summary reports that were circulated to sites[35,40,50–52]. Underperforming sites were asked to submit an action plan and provide additional staff training[50,52]. A clear escalation procedure was established for ensuring timely improvement of site performances[50]. Studies reported the importance of planning adequate human and financial resources for these tasks [30,38].

Communication

Regular communication with sites and investigators was critical[20,29,34–36,44,50,53]. Multiple communication channels were utilised to ensure consistency across sites, overcome time zone and cross-cultural differences. These included emails for study protocols, newsletters, progress reports and site score cards, and regular verbal communication in the form of monthly site teleconferences, outreach calls with leadership team and biannual or annual face-to-face meetings[7,20,34,36,44,50,53]. Communications were usually managed by a contract research organization, clinical trials unit or the site selection committee[48,50]. One study described in detail the guidelines they created to streamline the management of site queries and thereby limit unnecessary or excessive email traffic[34].

Trial management

For peer review only

Trial oversight

A number of management “tiers” could be identified, according to their scope of responsibility for trial oversight. Most studies described a Central Coordinating Centre (also referred to as a “trial coordinating centre”, “main operational centre” or “clinical or data coordinating centre”; CCC) with over-arching responsibility for overseeing trial setup and conduct in accordance with the protocol, SOPs and regulatory approvals[11,23,25,26,29,38,39,45,48,50–53]. The CCC worked with at least one national coordinating centre (also referred to as an “country coordinating centre” or “national project manager”) that supported regulatory approvals, set-up of sites and delivery of the trial in each country[19,25,33,44]. In trials spanning multiple continents, an additional level of management with an international coordinating centre worked closely with the CCC and was responsible for oversight of several national coordinating centres[11,23,54]. This arrangement ensured that there were data and regulatory consistencies between countries or research networks[44,51–53]. In general, the CCC consisted of the chief investigator, biostatistician, and support staff, being responsible for regulatory oversight, recruitment/retention [36], trial monitoring[19,33,45,48,51,52], secondary analyses and communication[11,23,36,50]. Other roles carried out by the CCC included overseeing finance and logistics including drug distribution, storage and analyses of specimen and community support[11,23,25]. Independent overall oversight of was provided by Data Safety Monitoring Board (or Committee; DSMB) and Trial Steering Committee (TSC) [53]. Most study reports referred to the International Conference on Harmonization Good Clinical Practice guidelines (ICH-GCP)[28,35,40,42,43,48,51,52].

Site and intervention selection

Site selection was often based upon initial feasibility assessments[43,46,49]. Several factors were considered during the assessment including local capacity or infrastructure[7,37,39,43,53], prevalence or burden of the disease in question[7,39,43], local ethics committees and regulatory processes[35,40,43,49], individual sites’ interest in participating[7], level of expertise available, and insight from local sites representatives[50]. The choice of intervention was usually based on expert reviews[7,44] or a systematic literature review[25,52] which was overseen by the TSC. Some studies also considered treatment guidelines[19,33] and expert consensus where evidence was sparse[41].

Recruitment

For peer review only - http://bmjopen.bmj.com/site/about/guidelines.xhtml

Recruitment strategies varied across the studies. Factors contributing to the difficulty in enrolling and retaining participants were complex and lengthy screening processes[27,49], financial constraints[37], difficulty in maintaining long-term site cooperation[49] and the rigour of navigating varied health systems and setups across countries. Country-specific laws further hampered recruitment: in a German study, participants could only enrol in one trial at a time[33]. Reported strategies that helped to overcome these challenges were frequent communication with participants through a combination of letters, phone calls, mailing of local medical publications containing the trial details and increasing study visibility through the use of websites[34,36,49,54] as well as implementation of the intervention at a convenient location[34]. Some studies organised educational sessions in the form of grand rounds and presentations to healthcare providers which increased visibility[34,36]. Reimbursement of transport costs and keeping visits to a minimum also increased recruitment[36,43].

For peer review only

Data management

Data management was not a major challenge and was generally overseen by the Data Coordinating Centre (DCC)[27,30,34,38,40,45,52]. Data was usually collected at trial sites using data collection forms and stored locally or in a web-based system. Studies which used the local storage model requested trial sites to securely send data to the DCC where it was amalgamated at regular intervals[34,38,45]. In contrast, use of a web-based system provided real time data entry, study updates and improved access[47,51,52,54]. Some studies reported technical challenges including problems with internet or firewalls, delays in data collection and missing data[34,40]. This was managed by ensuring sites had access to technical and training support[27,40].

Intervention management

Drug procurement and distribution

Pharmaceutical companies were the most common source of drug procurement[7,19,26,27,33,38,52,54], and in some cases the investigational medicinal product (IMP) was supplied free of charge[19,33,54]. In instances where there were drug import restrictions, local pharmacies and suppliers were used[7,36]. Most studies established a clinical coordinating pharmacy to oversee the translation, labelling, repackaging, shipping, and coordination of the drug distribution to the study sites[19,24,25,33,39,48]. Country-specific repository centres could be created, particularly in cross-continent studies. These handled the different regulatory and import requirements in addition to providing oversight and support for the distribution of IMPs across sites in their jurisdiction[19,33,48]. More

often, studies were faced with difficulty in navigating complex regulatory procedures between countries. For example, some studies in the EU were refused waiver for labelling requirements of a repurposed drug whereas a similar waiver request was granted in the UK[19,20,28,33]. Also, there were country-specific differences in import requirements and issues around “qualified person” drug release specifications[19,52]. Site pharmacies were involved in drug dispensing in some studies[26,29,36,48,49,51,52]. SOPs and training were provided to hospital pharmacists at each study site on drug procurement, dispensing, storage, maintenance of accountability logs and disposal of unused or expired drugs[7,51,52].

Safety monitoring and adverse events

Serious and adverse events (SAEs) at sites were reported either directly to the CCC or via an electronic data capture or web-based system, then to sponsor who reported to the relevant ethical and regulatory authority. One study ensured that a study coordinator, physician and safety officer were available at all times to manage emergency situations[36].

All 38 studies established a DSMB for monitoring drug safety and efficacy and safeguarding trial participants. SAEs were reviewed by the independent DSMB at varying intervals based on risk. [7,36,41,44]. The timing for These ranged from monthly, to biannually and annually. Some studies used a predetermined interval, for example, in one study the DSMB met every time at least 25-30 participants had a particular data point collected[25]. If there were concerns or SAEs, these meetings were brought forward. All monitoring activities were generally followed by reports which were circulated to all sites[25,53].

Biospecimen processing and transport

A variety of biospecimens were often processed in a different country[27,47,51,54,55]. Several studies asked sites to ship the specimen to the designated central facility for analysis[27,47,51,54,55]. Where trials spanned multiple continents, separate facilities were established in each [24,42,55]. Where a central facility for specimen storage was not feasible, a “virtual biobank” was sometimes established[53]. The collection, handling and processing of specimens were performed in accordance with SOPs[51,53]. Over-collection or under-collection of specimens, increased shipping costs for collection of additional samples, shipments delays, staff shortages and inadequate training were cited as challenges[27,50]. As most of the specimens were shipped to another country for analysis,

For peer review only

regulatory and administrative approvals, coupled with difficulties securing informed consent for international transfer presented additional hurdles[24,42]. Use of an electronic inventory for real-time monitoring of samples, ongoing laboratory training and efficient trial oversight were described as effective mitigation methods.

Adaptive-specific issues for international trials

Adaptive trial design has become a popular means of increasing the flexibility with which interventions may be interrogated with respect to particular disease indications and/or outcomes, and the efficiency with which this may be achieved (for example through interim analyses in the context of platform trials)[15]. They formed a substantial minority of the trials identified in the current review, reflecting challenges raised when implementing them across national boundaries. For example, compared to traditional trials, the need for significantly more documentation to provide clarity on the adaptive process was necessary, including treatment arms, plans for data collection and interim analyses[25,56]. Managing protocol amendments posed a further challenge. Studies reported that using an overarching master protocol and consent form and limiting IMP-specific information to appendices significantly expedited the time for ethics approval when adding or removing treatment arms[25,53]. Handling drug supply for multiple treatment arms whilst minimising waste was another recurring theme. Studies found it helpful to use pharmacies that could serve multiple clinical sites within a close geographical area, a centralised drug distribution system[25] and choosing countries with similar drug labelling requirements[56]. Data systems that enabled rapid data entry and analyses was another important consideration. Studies reported that setting up a streamlined system with pre-specified stopping rules for each treatment arm was helpful[35,53]. They also had predefined timelines for the frequency of data analysis [40,53,56].

Discussion

We conducted a systematic review to determine the operational complexities of conducting international trials, with the aim of providing a useful resource for researchers considering such an approach. Our search strategy employed an extensive array of search terms, themselves falling under four broad “concepts” and organised to optimise our ability to capture all relevant articles; this approach then necessitated significant refinement during the screening phase, and rigorous data collection methods. To this end, each abstract was reviewed by at least two authors for

For peer review only

inclusion and 20% of the full-text articles were screened by a second author. Our review highlights that there are various, consistent challenges in the planning, setup, delivery and close out phases of the 38 international trials. Some of the greatest challenges are posed by ethical and regulatory obstacles, a lack of harmonisation within EU and between EU and other developed countries being a key element. Similar findings have been highlighted in another systematic review on conducting trials in developing countries[57]. Streamlining funding and regulatory processes is one possible solution. The EU Clinical Trials Regulation launched a portal in 2022 that enables registration of trials with sites in up to 30 EU countries in a single platform. This system also resolves data sharing issues, providing a one-off consent and enabling national regulators to collaboratively process approvals[58]. However, there is no obligation for EU countries to participate until 2025 and this process does not provide a solution for collaboration with other continents. Furthermore, this portal does not extend to UK sites following its withdrawal from the EU. Possible interim solutions to global collaboration include defining responsibilities of sponsors and legal representatives, ascertaining insurance requirements prior to country selection and ensuring all sites are provided with a framework for the regulatory review process. In addition, working with a specialist insurance broker and careful country selection based on previous successful collaborations have also been suggested[59]. Establishing a national support group to provide mentoring to less experienced trialists for the entire trial process may also aid in the process[57]. By contrast, previous literature has described other key challenges including lack of infrastructure, poor data quality and lack of training particularly in resource-limited settings. These were not a particular challenge in our systematic review[57,60].

Budgetary constraints and contract negotiations were described as further hurdles. Over the last few years, several networks have been formed to increase funding opportunities. For example, INSIGHT was created to provide governance and funding support for multi-national HIV treatment trials but has expanded to also include influenza and COVID-19[61]. NeuroNEXT (Network for Excellence in Neuroscience Clinical Trials) promotes partnership in neurological disease trials by providing a centralised system for ethics review, contracting agreements and data management but is limited to multi-centre studies in the US[62]. Use of these networks, establishing site set-up costs during the trial planning phase and timely contract negotiations may assist in this process.

Managing drug distribution across international borders remains a challenge, as has also been previously reported[21]. Tailoring supply chains whilst minimising drug wastage is clearly desirable. Establishment of central

For peer review only

drug repositories in each continent and using pharmacies which can serve multiple sites rather than the traditional one-pharmacy-one-site model has been shown to be effective[19,48]. Involving pharmaceutical partners in drug distribution is another possibility which also eliminates the need for time-consuming vendor selection[63].

Our search strategy was employed an extensive array of search terms, themselves falling under four broad "concepts" and organised to optimise our ability to capture all relevant articles; this approach then necessitated significant refinement during the screening phase to arrive at robust and our data collection methods rigorous. Each abstract was reviewed by at least two authors for inclusion and 20% of the full-text articles were screened by a second author.

Limitations and future work.

The extent to which international facets of clinical trials is made explicit in their description, and the terminology used in the literature to describe clinical trials in general, have evolved over time, and this observation is reflected in the pragmatic search and screening strategy we adopted. As with any systematic review, we cannot exclude the possibility that decisions about individual terms included in, or excluded from, our searches may have influenced the precise range of papers pertaining to individual international trials that would have been captured in our systematic review – and hence the challenges and solutions identified during data extraction. Linked to this, not all investigators routinely publish information on challenges encountered in trial design and conduct, meaning our findings cannot be said to be exhaustive. Moreover, our systematic review only focused on trials conducted in more than one country and studies published after 2005 and therefore our results may not be generalisable to multi-centre studies conducted within the same country. For example, ethics regulations, sponsor responsibilities and insurance requirements are less likely to be a hurdle in single nation trials[63]. With respect to potential solutions to the challenges described, these were extracted from included papers where identified in association with specific challenges; whilst every effort was made to mandate this link, an element of subjectivity when summarising such solutions in the current report cannot be excluded. Finally, we did not assess clinical outcomes of included studies, considering this beyond the scope of our endeavour. Further research in the form of a qualitative or Delphi study with trialists and key stakeholders may provide more in-depth information on these challenges and possible actions to mitigate them. We also urge researchers involved in conducting international trials to routinely report operational challenges.

For peer review only

Conclusion

International trials address many unmet needs in trial design but their proponents still face operational challenges at every level, ranging from difficulties with funding and obtaining regulatory approvals to site contracts and drug distribution. Careful planning and communication with sites and key stakeholders during the trial planning phase can overcome delays presented by some of these challenges. More generally, given the upsurge in global trials particularly since the COVID-19 pandemic, recognition by policymakers of the potential rewards that regulatory harmonisation between nations could bring for the delivery of more efficient and cost-effective research should positively impact the health and wellbeing of their citizens. National and international organisations should continue to work collaboratively to develop infrastructures that support international trials.

Ethical approval

Not applicable.

Data sharing

No additional data available.

For peer review only

Tables

Table 1. Summary of included studies

First author (year)	Trial name	Trial design	Population	Number of sites	Site locations	Main coordinating centre(s)	Total participants	Intervention
Aban (2008)[25]	MGTX	Parallel	Myasthenia gravis	79	Global	USA	126	Other
Aitken (2008)[32]	PROMOTION	Parallel	Coronary artery disease	5	North America, Oceania	USA	3522	Behavioural
Angus (2020)[33]	REMAP-CAP	Adaptive-platform	Severe pneumonia and COVID-19		UK/EU, North America, Oceania, Asia	Australia, Thailand		Drug
Aryal (2021)[38]	REMAP-CAP	Adaptive-platform	Severe pneumonia and COVID-19		UK/EU, North America, Oceania, Asia	RCC in Australia and Thailand		Drug
Antic (2015)[42]	SAVE	Parallel	Obstructive sleep apnoea	89	Oceania, North America, South America, Asia, UK/EU	RCC in Australia, Brazil, China, India and Spain	2717	Device
Babiker (2013)[18]	START	Parallel	HIV	237	North America, South America, UK/EU, Oceania, Africa	RCC in Denmark, UK, Australia, USA	4000	Drug
Berthon-Jones (2015)[26]	ALTAIR	Parallel	HIV	36	Asia, Oceania, UK/EU, North America, South America		322	Drug
Bryant (2021)[49]	TBTC Study 31	Parallel	Tuberculosis	34	North America, South America, Asia, Africa	USA	2516	Drug
Carli (2013)[43]	SEYLE	Cluster	Suicide	11	UK/EU	Sweden	11110	Behavioural
Clasen (2020)[53]	HAPIN	Parallel	Low birth weight		Asia, North America, South America, Africa	USA		Device
Coomarasamy (2016)[50]	PROMISE	Parallel	Recurrent miscarriage	45	UK/EU	UK	836	Drug
Crow (2018)[19]	FOR-DMD	Parallel	Duchenne muscular dystrophy	40	North America, UK/EU	USA	196	Drug
del Álamo (2022)[20]								
Denholm (2022)[51]	ASCOT ADAPT	Adaptive-platform	COVID-19		Oceania, Asia			Drug
Dutton (2009)[44]	CONTROL	Parallel	Trauma	75	North America, South America, UK/EU, Asia, Africa	USA	576	Drug
Eikelboom (2022)[39]	ACT	Factorial	COVID-19	62	North America, South America, Africa, Asia		6528	Drug
Fogelholm (2017)[45]	PREVIEW	Factorial	Pre-diabetes	8	UK/EU, Oceania		2326	Behavioural
Franciscus (2014)[52]	TRIGR	Parallel	Type 1 diabetes	77	North America, Oceania, UK/EU	USA	5156	Other

For peer review only

Fulda (2023)[48]	REPRIEVE	Parallel	HIV		North America, South America, Africa, Asia, UK/EU	USA		Drug
Goossens (2021)[27]	REMAP-CAP	Adaptive-platform	Severe pneumonia and COVID-19		UK/EU, North America, Oceania, Asia	Australia, Thailand		Drug
Grarup (2015)[31]	START	Parallel	HIV	237	North America, South America, UK/EU, Oceania, Africa	RCC in Denmark, UK, Australia, USA	4000	Drug
Hata (2020)[40]	PATHWAY	Parallel	Breast cancer	23	Asia		185	Drug
Herrick (2012)[34]	FDTT	Parallel	Functional dyspepsia	8	North America		292	Drug
Jeon (2016)[46]	CLEAR III	Parallel	Intracerebral haemorrhage	73	North America, South America, UK/EU, Asia	USA	500	Drug
Kenyon (2011)[21]	STICH II	Parallel	Intracerebral haemorrhage	126	North America, Oceania, UK/EU, Asia, Africa	UK	601	Other
Kesho Bora Study Group (2011)[35]	Kesho-Bora	Parallel	HIV	5	Africa	Switzerland	824	Drug
Kolitsopoulos (2013)[47]	ZODIAC	Parallel	Schizophrenia	226	North America, South America, UK/EU, Asia		18240	Drug
Larson (2016)[22]	INSIGHT trials					RCC in UK, Denmark, USA and Australia		
Lingor (2021)[23]	ROCK-ALS / ROCK-ALS-US	Parallel	Amyotrophic lateral sclerosis		North America, UK/EU			Drug
Minisman (2012)[5]	MGTX	Parallel	Myasthenia gravis	79	Global	USA	126	Other
Murray (2022)[24]	TICO	Adaptive-platform	COVID-19		North America, UK/EU, Asia, Africa	USA with 8 RCC		Drug
Neaton (2010)[9]	INSIGHT trials					RCC in UK, Denmark, USA and Australia		
Ravinetto (2013)[36]	4ABC	Parallel	Malaria	12	Africa	Belgium	4112	Drug
Reams (2018)[41]	DOVE	Parallel	Sickle cell disease	51	North America, South America, UK/EU, Asia, Africa		341	Drug
Seal (2006)[28]		Factorial	Endophthalmitis	24	UK/EU, Asia	UK	35000	Drug
Spencer (2012)[54]	AWARD-5	Adaptive	Type 2 diabetes	111	North America, UK/EU, Asia		1202	Drug
Sydes (2011)[29]	STAMPEDE	Adaptive-platform	Prostate cancer		UK/EU	UK		Drug
Zimmer (2010)[37]	BAMSG 3-01	Parallel	Cryptococcal meningitis	13	North America, Asia	USA	143	Drug

RCC: Regional Coordinating Centre; RCT: Randomised Controlled Trial.

For peer review only

Table 2. Design characteristics of included trials (n=35)	
**Features**	**n (%)**
**Design**	
Parallel	24 (68.6)
Adaptive	7 (20.0)
Cluster	1 (2.9)
Factorial	3 (8.6)
**Masking**	
Open label	19 (54.3)
Blinded	16 (45.7)
**Number of sites among closed trials**: median (IQR)	40 (13 – 78)
**Continents of site locations**	
Africa	14 (40.0)
Asia	22 (62.9)
North America	27 (77.1)
Oceania	14 (40.0)
South America	14 (40.0)
UK/Europe	25 (71.4)
**Trial status**	
Open	9 (25.7)
Closed	25 (71.4)
Terminated	1 (2.9)
**Sample size among closed trials**: median (IQR)	1202 (332 – 4056)
**Intervention**	
Drug	26 (74.3)
Device	2 (5.7)
Behavioural	3 (8.6)
Other	4 (11.4)
**Primary outcome**	
Efficacy	14 (40.0)
Effectiveness	12 (34.3)
Safety	2 (5.7)
Prevention	7 (20.0)
**Sponsor**	
Academic	31 (88.6)
Industry	3 (8.6)
Unknown	1 (2.9)
**Main funder**	
Academic	1 (2.9)
Government	28 (80.0)
Industry	5 (14.3)
Charity	1 (2.9)

For peer review only

Table 3. Challenges and proposed solutions

	Challenges	Proposed solutions
Study set-up	Identification of appropriate sponsor and agreements	• Clarify roles and responsibilities between sponsor, co-sponsor and legal representative in advance<br>• Ascertain insurance policy requirements prior to country and site selection<br>• Identify a lead site for each country/continent that is familiar with local regulations to act as a coordinating centre for all local sites
	Budget considerations	• Establish site set-up costs early on (including start-up fees, pharmacy dispensing, lab, ethics, administration, archiving and close out)<br>• Consideration of changes in currency exchange rates<br>• Accurate budget predictions for trials spanning several years<br>• Translation costs
	Regulatory and ethical approvals	• Ensure all sites are familiar with the process required for regulatory approvals
Site set-up	Contracts with collaborating sites	• Begin contract negotiations early<br>• Establish coordinating sites or centres within each country or region to manage negotiations<br>• Allocate time and costs for translation<br>• Coordination with legal representatives to clarify country-specific terminology and interpretation of legal responsibilities and GDPR
	Site monitoring	• Plan adequate financial and human resources for in-person visits<br>• Clear escalation procedures and remedial actions for sites which are underperforming
	Channels of communication	• Regular communication with sites and investigators using multiple channels including emails, monthly teleconferences, outreach calls and annual face-to-face meetings<br>• Develop a hierarchical process for managing site queries to avoid unnecessary emails
	Translation	• Plan adequate financial resources and time for translation of essential documents, contracts and drug packaging
Trial management	Site and intervention selection	• Conduct site feasibility assessments during trial planning phase<br>• Use a systematic process to guide intervention selection and removal particularly for adaptive trials
	Recruitment and retention	• Frequent, often weekly communication with study participants using different channels (e.g. letters, phone calls, emails)<br>• Reimbursement of transport costs<br>• Keeping study visits to a minimum
Data management	Technological challenges including internet bandwidth, institutional firewalls and device availability	• Access to technical expertise and availability of real-time troubleshooting all the time<br>• Use of a software with an option for offline data entry
	Delays in data entry and missing data	• Training and retraining of researchers in data entry and protocol compliance
Intervention management	Difficulty in getting import permits	• Use local commercial or pharmacy suppliers
	Drug wastage	• Distribute smaller numbers but frequently replenish study sites
	Shipment delays and errors	• Establish an electronic inventory that allows real time monitoring of drug stock, biospecimen collection and distribution<br>• Staff training
Adaptive specific	Protocol amendments	• Use an overarching master protocol and consent form and add agent-specific information in appendices to speed up ethical reviews
	Handling drug supply for multiple arms	• Use pharmacies that can serve multiple sites within a close geographical area<br>• Centralised drug distribution system<br>• Choose countries with similar drug labelling requirements
	Data entry and analyses	• Predefine stopping rules for each treatment arm<br>• Predefine frequency of data analysis

For peer review only

Figure legends

Figure 1. PRISMA flow diagram.

Figure 2. Operational complexities of international trials.

References

1 Glickman SW, McHutchison JG, Peterson ED, *et al.* Ethical and Scientific Implications of the Globalization of Clinical Research. *N Engl J Med*. 2009;360:816–23.

2 Weigmann K. The ethics of global clinical trials: In developing countries, participation in clinical trials is sometimes the only way to access medical treatment. What should be done to avoid exploitation of disadvantaged populations? *EMBO Rep*. 2015;16:566–70.

3 Barrios CH, Werutsky G, Martinez-Mesa J. The global conduct of cancer clinical trials: challenges and opportunities. *Am Soc Clin Oncol Educ Book Am Soc Clin Oncol Annu Meet*. 2015;e132-139.

4 World Health Organization. WHO COVID-19 Solidarity Therapeutics Trial. 2021. https://www.who.int/emergencies/diseases/novel-coronavirus-2019/global-research-on-novel-coronavirus-2019-ncov/solidarity-clinical-trial-for-covid-19-treatments (accessed 23 March 2023)

5 Lang T, Siribaddana S. Clinical trials have gone global: is this a good thing? *PLoS Med*. 2012;9:e1001228.

6 Thiers FA, Sinskey AJ, Berndt ER. Trends in the globalization of clinical trials. *Nat Rev Drug Discov*. 2008;7:13–4.

7 Minisman G, Bhanushali M, Conwit R, *et al.* Implementing clinical trials on an international platform: challenges and perspectives. *J Neurol Sci*. 2012;313:1–6.

8 Jeong S, Sohn M, Kim JH, *et al.* Current globalization of drug interventional clinical trials: characteristics and associated factors, 2011–2013. *Trials*. 2017;18:288.

9 The Adaptive Platform Trials Coalition. Adaptive platform trials: definition, design, conduct and reporting considerations. *Nat Rev Drug Discov*. 2019;18:797–807.

10 Pocock S, Calvo G, Marrugat J, *et al.* International differences in treatment effect: do they really exist and why?†. *Eur Heart J*. 2013;34:1846–52.

11 Neaton JD, Babiker A, Bohnhorst M, *et al.* Regulatory impediments jeopardizing the conduct of clinical trials in Europe funded by the National Institutes of Health. *Clin Trials*. 2010;7:705–18.

12 Tang M, Joensuu H, Simes RJ, *et al.* Challenges of international oncology trial collaboration-a call to action. *Br J Cancer*. 2019;121:515–21.

13 Kurzrock R, Pilat S, Bartolazzi M, *et al.* Project Zero Delay: a process for accelerating the activation of cancer clinical trials. *J Clin Oncol Off J Am Soc Clin Oncol*. 2009;27:4433–40.

14 Lai J, Forney L, Brinton DL, *et al.* Drivers of Start-Up Delays in Global Randomized Clinical Trials. *Ther Innov Regul Sci*. 2021;55:212–27.

15 Chow S-C, Corey R. Benefits, challenges and obstacles of adaptive clinical trial designs. *Orphanet J Rare Dis*. 2011;6:79.

For peer review only

16 Brown LC, Graham J, Fisher D, *et al.* Experiences of running a stratified medicine adaptive platform trial: Challenges and lessons learned from 10 years of the FOCUS4 trial in metastatic colorectal cancer. *Clin Trials*. 2022;19:146–57.

17 Page MJ, McKenzie JE, Bossuyt PM, *et al.* The PRISMA 2020 statement: an updated guideline for reporting systematic reviews. *BMJ*. 2021;n71.

18 Gumber L, Pratt A, Bardgett M, *et al.* Operational best practice in international clinical trials: a systematic review protocol. Published Online First: 30 January 2023. doi: 10.17605/OSF.IO/YVTJB

19 Babiker AG, Emery S, Fatkenheuer G, *et al.* Considerations in the rationale, design and methods of the Strategic Timing of AntiRetroviral Treatment (START) study. *Clin Trials*. 2013;10:S5–36.

20 Crow RA, Hart KA, McDermott MP, *et al.* A checklist for clinical trials in rare disease: obstacles and anticipatory actions-lessons learned from the FOR-DMD trial. *Trials Electron Resour*. 2018;19:291.

21 Del Alamo M, Buhrer C, Fisher D, *et al.* Identifying obstacles hindering the conduct of academic-sponsored trials for drug repurposing on rare-diseases: an analysis of six use cases. *Trials Electron Resour*. 2022;23:783.

22 Kenyon GM, Mendelow AD, Gregson BA, *et al.* Obtaining regulatory approval for multicentre randomised controlled trials: experiences in the STICH II trial. *Br J Neurosurg*. 2011;25:352–6.

23 Larson GS, Carey C, Grarup J, *et al.* Lessons learned: Infrastructure development and financial management for large, publicly funded, international trials. *Clin Trials*. 2016;13:127–36.

24 Lingor P, Koch JC, Statl, *et al.* Challenges and opportunities for Multi-National Investigator-Initiated clinical trials for ALS: European and United States collaborations. *Amyotroph Lateral Scler Front Degener*. 2021;22:419–25.

25 Murray DD, Babiker AG, Baker JV, *et al.* Design and implementation of an international, multi-arm, multi-stage platform master protocol for trials of novel SARS-CoV-2 antiviral agents: Therapeutics for Inpatients with COVID-19 (TICO/ACTIV-3). *Clin Trials*. 2022;19:52–61.

26 Aban IB, Wolfe GI, Cutter GR, *et al.* The MGTX experience: challenges in planning and executing an international, multicenter clinical trial. *J Neuroimmunol*. 2008;201:80–4.

27 Berthon-Jones N, Courtney-Vega K, Donaldson A, *et al.* Assessing site performance in the Altair study, a multinational clinical trial. *Trials Electron Resour*. 2015;16:138.

28 Goossens H, Derde L, Horby P, *et al.* The European clinical research response to optimise treatment of patients with COVID-19: lessons learned, future perspective, and recommendations. *Lancet Infect Dis*. 2022;22:e153–8.

30 Seal DV, Barry P, Gettinby G, *et al.* ESCRS study of prophylaxis of postoperative endophthalmitis after cataract surgery. Case for a European multicenter study. *J Cataract Refract Surg*. 2006;32:396–406.

31 Sydes MR, Parmar MK, Mason MD, *et al.* Flexible trial design in practice - stopping arms for lack-of-benefit and adding research arms mid-trial in STAMPEDE: a multi-arm multi-stage randomized controlled trial. *Trials Electron Resour*. 2012;13:168.

32 Official Journal of the European Union. Regulation (EU) No 536/2014 of the European Parliament and of the Council of 16 April 2014 on clinical trials on medicinal products for human use, and repealing Directive 2001/20/EC Text with EEA relevance. 2022. https://eur-lex.europa.eu/legal-content/EN/TXT/?uri=CELEX%3A02014R0536-20221205 (accessed 4 May 2023)

For peer review only

33 Grarup J, Rappoport C, Engen NW, *et al.* Challenges, successes and patterns of enrolment in the INSIGHT Strategic Timing of AntiRetroviral Treatment (START) trial. *HIV Med*. 2015;16:14–23.

34 Aitken LM, Pelter MM, Carlson B, *et al.* Effective strategies for implementing a multicenter international clinical trial. *J Nurs Scholarsh*. 2008;40:101–8.

35 Angus DC, Berry S, Lewis RJ, *et al.* The REMAP-CAP (Randomized Embedded Multifactorial Adaptive Platform for Community-acquired Pneumonia) Study. Rationale and Design. *Ann Am Thorac Soc*. 2020;17:879–91.

36 Herrick LM, Locke 3rd GR, Zinsmeister AR, *et al.* Challenges and lessons learned in conducting comparative-effectiveness trials. *Am J Gastroenterol*. 2012;107:644–9.

37 Kesho Bora Study G. Safety and effectiveness of antiretroviral drugs during pregnancy, delivery and breastfeeding for prevention of mother-to-child transmission of HIV-1: the Kesho Bora Multicentre Collaborative Study rationale, design, and implementation challenges. *Contemp Clin Trials*. 2011;32:74–85.

38 Ravinetto RM, Talisuna A, De Crop M, *et al.* Challenges of non-commercial multicentre North-South collaborative clinical trials. *Trop Med Int Health*. 2013;18:237–41.

39 Zimmer LO, Nolen TL, Pramanpol S, *et al.* International collaboration between US and Thailand on a clinical trial of treatment for HIV-associated cryptococcal meningitis. *Contemp Clin Trials*. 2010;31:34–43.

40 Aryal D, Beane A, Dondorp AM, *et al.* Operationalisation of the Randomized Embedded Multifactorial Adaptive Platform for COVID-19 trials in a low and lower-middle income critical care learning health system. *Wellcome Open Res*. 2021;6:14.

41 Eikelboom J, Rangarajan S, Jolly SS, *et al.* The Anti-Coronavirus Therapies (ACT) Trials: Design, Baseline Characteristics, and Challenges. *CJC Open*. 2022;4:568–76.

42 Hata T, Nakamura K, Yonemori K, *et al.* Regulatory and operational challenges in conducting Asian International Academic Trial for expanding the indications of cancer drugs. *Clin Transl Sci*. 2021;14:1015–25.

43 Reams SG, Messana L, Brown PB, *et al.* Clinical trial conduct in special populations and developing regions: An overview of the DOVE study in pediatric patients with sickle cell disease. *Contemp Clin Trials*. 2018;64:88–94.

44 Antic NA, Heeley E, Anderson CS, *et al.* The Sleep Apnea cardioVascular Endpoints (SAVE) Trial: Rationale, Ethics, Design, and Progress. *Sleep*. 2015;38:1247–57.

45 Carli V, Wasserman C, Wasserman D, *et al.* The saving and empowering young lives in Europe (SEYLE) randomized controlled trial (RCT): methodological issues and participant characteristics. *BMC Public Health*. 2013;13:479.

46 Dutton R, Hauser C, Boffard K, *et al.* Scientific and logistical challenges in designing the CONTROL trial: recombinant factor VIIa in severe trauma patients with refractory bleeding. *Clin Trials*. 2009;6:467–79.

47 Fogelholm M, Larsen TM, Westerterp-Plantenga M, *et al.* PREVIEW: Prevention of Diabetes through Lifestyle Intervention and Population Studies in Europe and around the World. Design, Methods, and Baseline Participant Description of an Adult Cohort Enrolled into a Three-Year Randomised Clinical Trial. *Nutrients*. 2017;9:20.

For peer review only

48 Jeon JE, Mighty J, Lane K, *et al.* Participation of a coordinating center pharmacy in a multicenter international study. *Am J Health Syst Pharm*. 2016;73:1859–68.

49 Kolitsopoulos FM, Strom BL, Faich G, *et al.* Lessons learned in the conduct of a global, large simple trial of treatments indicated for schizophrenia. *Contemp Clin Trials*. 2013;34:239–47.

50 Fulda ES, Fichtenbaum CJ, Kileel EM, *et al.* The importance of methods for site performance evaluation in REPRIEVE, a longitudinal, global, multicenter trial. *Contemp Clin Trials*. 2023;124. doi: 10.1016/j.cct.2022.107035

51 Bryant KE, Yuan Y, Engle M, *et al.* Central monitoring in a randomized, open-label, controlled phase 3 clinical trial for a treatment-shortening regimen for pulmonary tuberculosis. *Contemp Clin Trials*. 2021;104. doi: 10.1016/j.cct.2021.106355

52 Coomarasamy A, Williams H, Truchanowicz E, *et al.* PROMISE: First-trimester progesterone therapy in women with a history of unexplained recurrent miscarriages - A randomised, double-blind, placebo-controlled, international multicentre trial and economic evaluation. *Health Technol Assess*. 2016;20:7–91.

53 Denholm JT, Venkatesh B, Davis J, *et al.* ASCOT ADAPT study of COVID-19 therapeutics in hospitalised patients: an international multicentre adaptive platform trial. *Trials Electron Resour*. 2022;23:1014.

54 Franciscus M, Nucci A, Bradley B, *et al.* Recruitment and retention of participants for an international type 1 diabetes prevention trial: a coordinators' perspective. *Clin Trials*. 2014;11:150–8.

55 Clasen T, Checkley W, Peel JL, *et al.* Design and Rationale of the HAPIN Study: A Multicountry Randomized Controlled Trial to Assess the Effect of Liquefied Petroleum Gas Stove and Continuous Fuel Distribution. *Environ Health Perspect*. 2020;128:47008.

56 Spencer K, Colvin K, Braunecker B, *et al.* Operational challenges and solutions with implementation of an adaptive seamless phase 2/3 study. *J Diabetes Sci Technol*. 2012;6:1296–304.

57 Alemayehu C, Mitchell G, Nikles J. Barriers for conducting clinical trials in developing countries- a systematic review. *Int J Equity Health*. 2018;17:37.

58 European Medicines Agency. Clinical Trials Regulation. Eur. Med. Agency. 2022. https://www.ema.europa.eu/en/human-regulatory/research-development/clinical-trials/clinical-trials-regulation (accessed 25 April 2023)

59 Fox L, Toms C, Kernaghan S, *et al.* Conducting non-commercial international clinical trials: the ICR-CTSU experience. *Trials Electron Resour*. 2017;18:440.

60 Mbuagbaw L, Thabane L, Ongolo-Zogo P, *et al.* The challenges and opportunities of conducting a clinical trial in a low resource setting: the case of the Cameroon mobile phone SMS (CAMPS) trial, an investigator initiated trial. *Trials*. 2011;12:145.

61 International Network for Strategic Initiative in Global HIV Trials. New science in the International Network for Strategic Initiatives in Global HIV Trials (INSIGHT): Why, What and How. 2011. http://insight.ccbr.umn.edu/governance_committees/ssc/WhyWhatWhoToDoResearch.pdf (accessed 9 March 2023)

62 Love MN, Fathallh-Shaykh H. Network for Excellence in Neuroscience Clinical Trials: NeuroNEXT. *JAMA Neurol*. Published Online First: 5 August 2013. doi: 10.1001/jamaneurol.2013.3663

For peer review only

63 Snowdon C, Kernaghan S, Moretti L, *et al.* Operational complexity versus design efficiency: challenges of implementing a phase IIa multiple parallel cohort targeted treatment platform trial in advanced breast cancer. *Trials*. 2022;23:372.

For peer review only

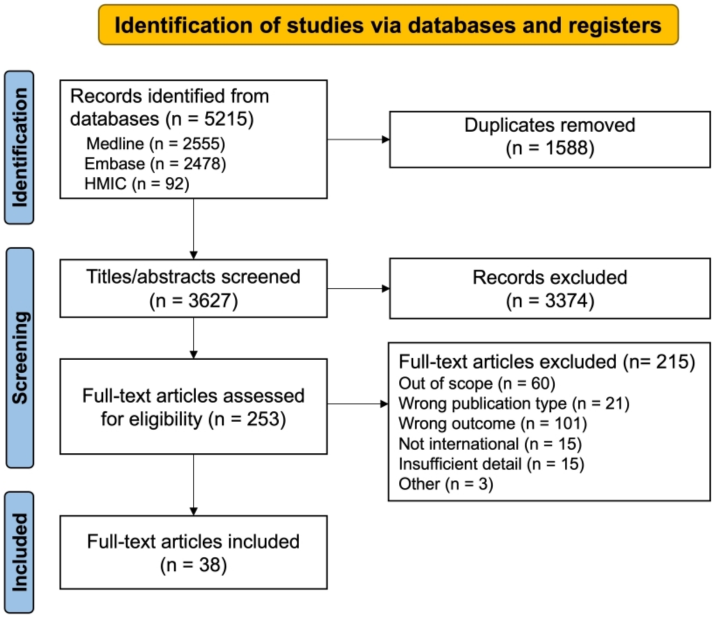

PRISMA Flow diagram

254x190mm (400 x 400 DPI)

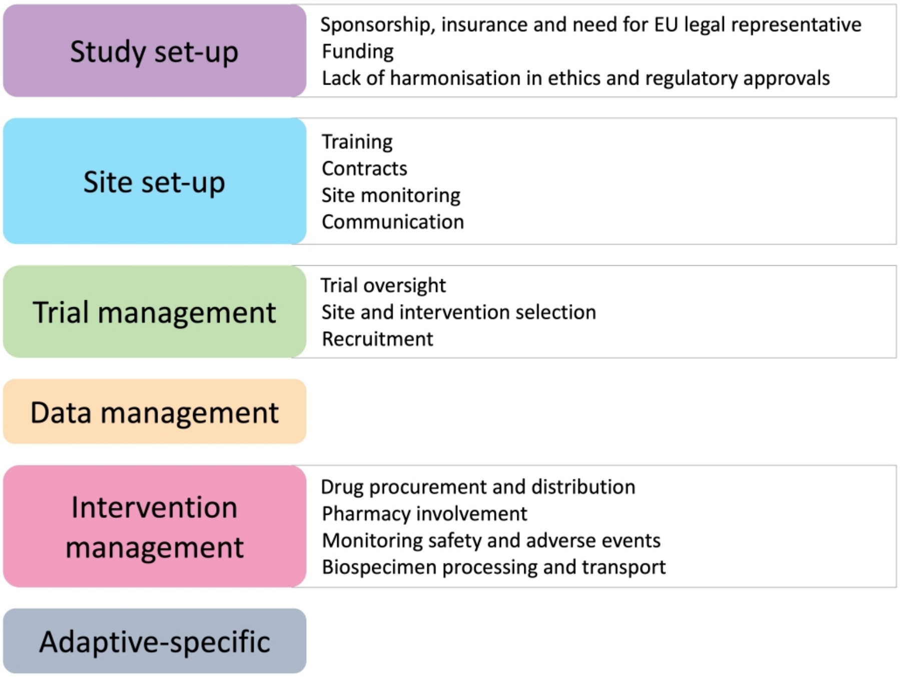

Figure 2. Operational complexities of international trials

254x190mm (400 x 400 DPI)

Supplementary Material

1 LITERATURE SEARCH STRATEGY

A summary of the search and selection strategy is first depicted for quick reference (a); examples of the kinds of search terms that were used to identify each of four concepts, as described in the main manuscript are then provided in (b). Full and detailed database search strategies used in Medline, Embase and HMIC are then also provided (c) for reference.

a) Search and selection strategy

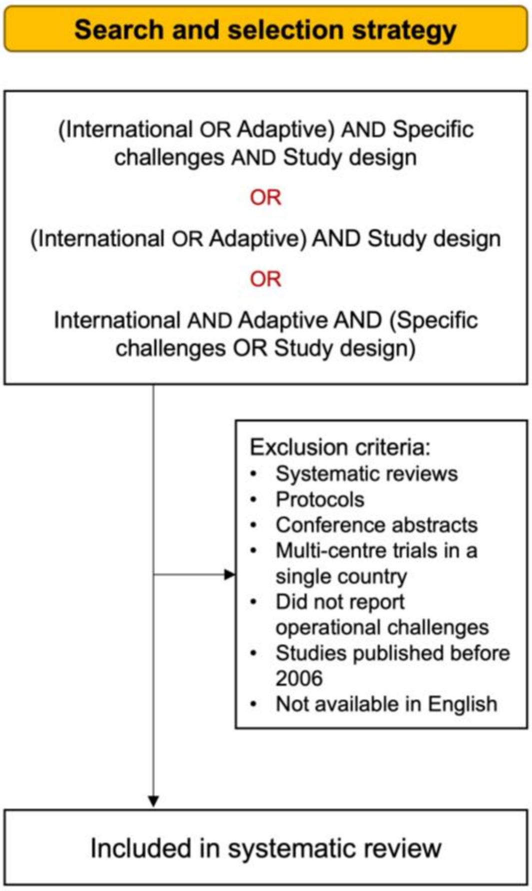

b) Examples of search terms used for each concept

International
International, multinational, intercontinental, multicentre, multisite, pan-European.

Adaptive
Adaptive, platform, umbrella, basket, bucket, master protocol.

Specific challenges
legal, operational, workforce, data management, insurance, indemnity, add or remove arms, gdpr, infrastructure, recruitment, sponsor, contract research, law, regulation, budget, finance, resource allocation, resource management, policy, Brexit, intervention, investigational, supply, logistics, distribution, delivery, monitoring, safety, capacity building, contract, funding, delegation, agreement, ethics, insurance, data sharing.

Study design
lessons, pitfalls, study design, methodology, challenge, operational, practical, guidelines, recommendations, rationale, implement, workflow, framework.

For peer review only - http://bmjopen.bmj.com/site/about/guidelines.xhtml

c) Detailed database search strategy.

Database: MEDLINE (Ovid), 31 January 2023

1	adaptive clinical trial/	37
2	((adaptive or umbrella or basket or bucket) adj2 (design or trial or trials or study or studies or protocol$)).mp.	4428
3	(trial$ platform$ or platform design or platform trial$ or platform clinical trial$ or platform study or platform studies or platform protocol$).mp.	856
4	((adaptive adj6 design$) and (trial or trials or study or studies or protocol$)).mp.	3077
5	(complex innovative adj3 (trial$ or design$ or protocol$)).mp.	17
6	master protocol$.mp.	242
7	response adaptive randomi$.mp.	139
8	(single centre or single site).mp.	33986
9	(or/1-7) not 8 [adaptive design - not single-site]	6597
10	(international adj5 (trial or trials or protocol$ or multicent$ or multi-cent$ or multisite or multi-site or multi-arm or multiarm or multi-stage or multistage)).mp.	19111
11	(international clinical$ trial$ regist$ or international clinical$ trial$ platform$ or international standard randomi$ control$ trial$ number$).mp.	3897
12	international.mp. /freq=2	93924
13	10 not (11 not 12) [gets rid of results with 'international trial' that only refer to the WHO ICTRP]	15678
14	((multinational or multi-national or intercontinental or inter-continental or pan-europ$) adj8 (trial or trials or protocol$ or multicent$ or multi-cent$ or multisite or multi-site)).mp.	3011
15	((trial or trials or protocol$) and (((multicent$ or multi-cent$ or multisite or multi-site) and sites) or centres or centers) and countries).mp.	5427
16	((UK or United Kingdom) and (EU or Europ$) and (trial$ or protocol$)).mp.	2982
17	(europe/ or european alpine region/ or andorra/ or austria/ or balkan peninsula/ or belgium/ or exp europe, eastern/ or exp france/ or exp germany/ or gibraltar/ or greece/ or ireland/ or exp italy/ or liechtenstein/ or luxembourg/ or exp mediterranean region/ or monaco/ or netherlands/ or portugal/ or san marino/ or exp "scandinavian and nordic countries"/ or spain/ or switzerland/ or exp transcaucasia/ or exp ussr/ or vatican city/) and exp United Kingdom/ and (trial$ or protocol$).mp.	1404
18	(exp africa/ or exp americas/ or exp asia/ or exp oceania/) and exp europe/ and (trial$ or protocol$).mp.	9851
19	or/13-18 [main international trial requirement]	35699
20	Methods/	231754
21	Research Design/	122769
22	challenge$.mp.	870696
23	(((trial$ or design) adj5 efficiency) or efficiencies).mp.	54394
24	((complexit$ or guidance or guidelines or recommendations or considerations or issues or obstacles or barriers) and trial$).ti.	5122
25	((lesson or lessons or pitfall or pitfalls) and (design$ or plan$)).mp.	27395
26	(trial$ adj5 (manag$ or run or running or conducting)).mp.	15671
27	(design$ or rationale$ or implement$ or methodol$).ti.	292767
28	(trial$ and (framework$ or regulations)).ti.	488
29	(trials and (Europ$ or EU or multinational or multi-national or intercontinental or inter-continental or international or multi-cent$ or multicent$ or multi-site or multisite)).ti.	2853
30	(trial$ adj1 (approval or authorisation or authorization or regulation)).mp.	341
31	"organization and administration"/ or capacity building/ or decision making, organizational/ or efficiency/ or efficiency, organizational/ or organizational culture/ or workforce/ or models, organizational/ or organizational innovation/ or change management/ or organizational objectives/ or personnel management/ or leadership/ or personnel selection/ or "personnel staffing and scheduling"/ or staff development/ or planning techniques/ or strategic planning/ or program development/ or total quality management/	306062
32	Multicenter Studies as Topic/	22031
33	exp *Clinical Trials as Topic/ or exp Clinical Trials as Topic/mt, st	68990
34	(trial$ and design$).kf.	2312

For peer review only - http://bmjopen.bmj.com/site/about/guidelines.xhtml

35	(((good or best) adj3 practice$) and trial$).ti.	204
36	(trial$ adj2 (authoris$ or authoriz$)).mp.	110
37	or/20-36 [general study design/methodology terms]	1888239
38	((trial$ adj5 insur$) or insuring or indemni$).mp.	2674
39	((add$ or remov$) adj5 (arm or arms)).mp.	3631
40	gdpr.mp.	365
41	exp *ethics/ or ethic$.ti.	121779
42	((data adj8 secur$) or (data adj3 (share$ or sharing)) or data management or data governance).mp. or computer security/ or data anonymization/	33659
43	data accuracy/	3708
44	(trial$ adj3 infrastructure).mp.	197
45	exp *models, statistical/ or (exp models, statistical/ and ((statistical adj3 (model$ or framework$ or plan$)) or estimand$).ti,ab.) or (statistical adj1 design$).mp.	59261
46	software/ or database management systems/ or software design/ or software validation/	133275
47	(reporting guideline$ or outcome reporting).kf. or reporting.ti. or (reporting adj4 (quality or guideline$)).mp.	37866
48	terminology/	0
49	(recruit$ adj4 (effective$ or model$ or pause$ or pausing)).mp. or recruit$.ti. or *Patient Selection/	50660
50	((decision$ adj2 algorithm$).mp.	2965
51	sponsor$.ti.	3863
52	fund$.ti.	43682
53	resourcing.mp.	1190
54	exp financial management/ or financing, organized/ or taxes/ or (financ$ not incentiv$).ti.	118701
55	economic$.ti.	57184
56	(trial$ adj1 cost$).mp. or (costs or costing).ti.	39270
57	contract research org$.mp.	428
58	(regulations or (regulat$ adj3 framework$)).mp.	60029
59	Government Regulation/	21840
60	jurisprudence/ or confidentiality/ or personally identifiable information/ or international law/ or legal services/ or liability, legal/ or mandatory reporting/ or legislation, drug/	80153
61	((legal$ or law or laws or legislat$ or policy or policies or rule or rules) adj5 (UK or United Kingdom or brit$ or EU or Europe$ or framework$ or national or government$)).mp.	50560
62	(clinical trial regulation or "536/2014").mp.	95
63	((EU or Europe or European) adj4 representative$).mp.	810
64	collaborat$.ti.	37346
65	(harmonis$ or harmoniz$).mp.	20656
66	brexit.mp.	771
67	(safety adj3 (monitor$ or governance)).mp. or Patient Safety/	31531
68	(((intervention or IMP or investigational medicin$ or drug$) adj6 (supply$ or inventory or export$ or import$)) or procur$).mp.	130269
69	(treatment selection or site selection).mp.	8819
70	workload$.mp.	51329
71	Contracts/ or (contract or contracts).mp.	37008
72	((fund$ or collaborat$ or delegat$ or site or sites or research or sponsor$) adj5 (agreement or agreements)).mp.	3553
73	((operational or practical or legal or administrative or financial or procedural or ethical or methodolog$ or statistical or recruitment or sponsor$ or logistic$ or design) adj4 (complexit$ or guidance or guidelines or recommendations or considerations or issues or challenges or obstacles or barriers or difficulties or advantages or disadvantages)).mp.	135867
74	exp *Clinical Trials as Topic/es, lj, og, st, sd [Ethics, Legislation & Jurisprudence, Organization & Administration, Supply & Distribution]	11020
75	or/38-74 [any of the various specific challenges]	1238686
76	(9 or 19) and 37 and 75	2818
77	(9 or 19) and (*Research Design/ or *Methods/)	1335
78	9 and 19 and (37 or 75)	72
79	76 or 77 or 78	3675
80	limit 79 to yr="2006 -Current"	3065
81	80 and (exp clinical trial/ or trial$.mp.)	2686

For peer review only

82	limit 81 to ("review" or "scientific integrity review" or "systematic review")	498
83	81 not (82 and (systematic and review).ti.)	2630
84	83 not (exp animals/ not humans.sh.)	2625
85	limit 84 to english language	2555

Database: EMBASE, 31 January 2023

1	adaptive clinical trial/	377
2	((adaptive or umbrella or basket or bucket) adj2 (design or trial or trials or study or studies or protocol$)).mp.	6949
3	(trial$ platform$ or platform design or platform trial$ or platform clinical trial$ or platform study or platform studies or platform protocol$).mp.	1567
4	((adaptive adj6 design$) and (trial or trials or study or studies or protocol$)).mp.	4842
5	(complex innovative adj3 (trial$ or design$ or protocol$)).mp.	29
6	master protocol$.mp.	439
7	response adaptive randomi$.mp.	207
8	(single centre or single site).mp.	66742
9	(or/1-7) not 8 [adaptive design - not single-site]	10465
10	(international adj5 (trial or trials or protocol$ or multicent$ or multi-cent$ or multisite or multi-site or multi-arm or multiarm or multi-stage or multistage)).mp.	32583
11	(international clinical$ trial$ regist$ or international clinical$ trial$ platform$ or international standard randomi$ control$ trial$ number$).mp.	4034
12	international.mp. /freq=2	165479
13	10 not (11 not 12) [gets rid of results with 'international trial' that only refer to the WHO ICTRP]	29033
14	((multinational or multi-national or intercontinental or inter-continental or pan-europ$) adj8 (trial or trials or protocol$ or multicent$ or multi-cent$ or multisite or multi-site)).mp.	5901
15	((trial or trials or protocol$) and (((multicent$ or multi-cent$ or multisite or multi-site) and sites) or centres or centers) and countries).mp.	9439
16	((UK or United Kingdom) and (EU or Europ$) and (trial$ or protocol$)).mp.	8673
17	(western europe/ or austria/ or exp belgium/ or benelux/ or exp france/ or germany/ or ireland/ or liechtenstein/ or luxembourg/ or monaco/ or netherlands/ or exp scandinavia/ or switzerland/ or exp Eastern Europe/ or exp Southern Europe/) and exp United Kingdom/ and (trial$ or protocol$).mp.	5785
18	((exp Eastern Europe/ and exp Southern Europe/) or (exp Eastern Europe/ and exp Western Europe/) or (exp Western Europe/ and exp Southern Europe/)) and (trial$ or protocol$).mp.	6768
19	(exp western hemisphere/ or exp africa/ or exp asia/ or exp "australia and new zealand"/) and exp Europe/ and (trial$ or protocol$).mp.	25734
20	or/13-19 [main international trial requirement]	77781
21	methodology/	1630438
22	study design/	54726
23	challenge$.mp.	1076548
24	(((trial$ or design) adj5 efficiency) or efficiencies).mp.	59423
25	((complexit$ or guidance or guidelines or recommendations or considerations or issues or obstacles or barriers) and trial$).ti.	6537
26	((lesson or lessons or pitfall or pitfalls) and (design$ or plan$)).mp.	35412
27	(trial$ adj5 (manag$ or run or running or conducting)).mp.	25255
28	(design$ or rationale$ or implement$ or methodol$).ti.	345887
29	(trial$ and (framework$ or regulations)).ti.	629
30	(trials and (Europ$ or EU or multinational or multi-national or intercontinental or inter-continental or international or multi-cent$ or multicent$ or multi-site or multisite)).ti.	4222
31	(trial$ adj1 (approval or authorisation or authorization or regulation)).mp.	579
32	management/ or joint venture/ or total quality management/ or work schedule/ or workflow/	183307
33	"multicenter study (topic)"/	38773
34	exp *"clinical trial (topic)"/	21048
35	(trial$ and design$).kf.	4092
36	(((good or best) adj3 practice$) and trial$).ti.	301

37	(trial$ adj2 (authoris$ or authoriz$)).mp.	223
38	or/21-37 [general study design/methodology terms]	3300880
39	((trial$ adj5 insur$) or insuring or indemni$).mp.	3566
40	((add$ or remov$) adj5 (arm or arms)).mp.	6264
41	gdpr.mp.	528
42	exp *ethics/ or ethic$.ti.	134708
43	((data adj8 secur$) or (data adj3 (share$ or sharing)) or data management or data governance).mp. or data protection/ or anonymization/ or data privacy/ or encryption/	37340
44	data quality/ or data accuracy/ or data availability/ or data completeness/ or data consistency/ or data validity/	8842
45	(trial$ adj3 infrastructure).mp.	316
46	*statistical model/ or *statistical analysis/ or ((statistical model/ or statistical analysis/) and ((statistical adj3 (model$ or framework$ or plan$)) or estimand$).ti,ab.) or statistical design/ or (statistical adj1 design$).mp.	55949
47	software/ or software design/ or general software/ or exp data analysis software/	203465
48	(reporting guideline$ or outcome reporting).kf. or reporting.ti. or (reporting adj4 (quality or guideline$)).mp.	48775
49	nomenclature/	60593
50	(recruit$ adj4 (effective$ or model$ or pause$ or pausing)).mp. or recruit$.ti. or *patient selection/	51069
51	(decision$ adj2 algorithm$).mp.	4194
52	sponsor$.ti.	4495
53	funding/ or fund$.ti.	114234
54	resourcing.mp.	1588
55	financial management/ or accounting/ or "billing and claims"/ or budget/ or finance/ or corporate finance/ or public finance/ or purchasing/ or exp tax/ or resource management/ or resource allocation/ or (financ$ not incentiv$).ti.	233201
56	economic evaluation/ or economic$.ti.	81922
57	(trial$ adj1 cost$).mp. or (costs or costing).ti.	52721
58	contract research org$.mp.	934
59	(regulations or (regulat$ adj3 framework$)).mp.	77414
60	*european medicines agency/ or *"medicines and healthcare products regulatory agency"/	604
61	legal aspect/ or government regulation/ or legal evidence/ or legal liability/ or legal procedure/ or legal service/ or medical liability/ or medicolegal aspect/ or jurisprudence/	281458
62	((legal$ or law or laws or legislat$ or policy or policies or rule or rules) adj5 (UK or United Kingdom or brit$ or EU or Europe$ or framework$ or national or government$)).mp.	68360
63	(clinical trial regulation or "536/2014").mp.	168
64	((EU or Europe or European) adj4 representative$).mp.	1071
65	collaborat$.ti.	45453
66	(harmonis$ or harmoniz$).mp.	31555
67	brexit.mp.	929
68	(safety adj3 (monitor$ or governance)).mp.	11693
69	(((intervention or IMP or investigational medicin$ or drug$) adj6 (supply$ or inventory or export$ or import$)) or procur$).mp.	114343
70	(treatment selection or site selection).mp.	12860
71	workload$.mp.	72251
72	contract/ or (contract or contracts).mp.	38623
73	((fund$ or collaborat$ or delegat$ or site or sites or research or sponsor$) adj5 (agreement or agreements)).mp.	5107
74	((operational or practical or legal or administrative or financial or procedural or ethical or methodolog$ or statistical or recruitment or sponsor$ or logistic$ or design) adj4 (complexit$ or guidance or guidelines or recommendations or considerations or issues or challenges or obstacles or barriers or difficulties or advantages or disadvantages)).mp.	168395
75	or/39-74 [any of the various specific challenges]	1787225
76	(9 or 20) and 38 and 75	4552
77	(9 or 20) and (*study design/ or *methodology/)	932
78	9 and 20 and (38 or 75)	192

For peer review only

79	76 or 77 or 78	5246
80	limit 79 to conference abstract	1834
81	79 not 80	3412
82	limit 81 to yr="2006 -Current"	3004
83	82 and (trial$.mp. or clinical research/ or exp clinical trial/)	2633
84	limit 83 to ("systematic review" or "review")	656
85	83 not (84 and (systematic and review).ti.)	2567
86	85 not ((exp animal/ or animal experiment/ or nonhuman/) not (exp human/ or human experiment/))	2557
87	limit 86 to english language	2478

Database: Health Management Information Consortium, 31 January 2023

1	((adaptive or umbrella or basket or bucket) adj2 (design or trial or trials or study or studies or protocol$)).mp.	15
2	(trial$ platform$ or platform design or platform trial$ or platform clinical trial$ or platform study or platform studies or platform protocol$).mp.	4
3	((adaptive adj6 design$) and (trial or trials or study or studies or protocol$)).mp.	8
4	(complex innovative adj3 (trial$ or design$ or protocol$)).mp.	0
5	master protocol$.mp.	1
6	response adaptive randomi$.mp.	0
7	(single centre or single site).mp.	129
8	(or/1-6) not 7 [adaptive design - not single-site]	22
9	(international adj5 (trial or trials or protocol$ or multicent$ or multi-cent$ or multisite or multi-site or multi-arm or multiarm or multi-stage or multistage)).mp.	114
10	(international clinical$ trial$ regist$ or international clinical$ trial$ platform$ or international standard randomi$ control$ trial$ number$).mp.	29
11	international.mp. /freq=2	3011
12	9 not (10 not 11) [gets rid of results with 'international trial' that only refer to the WHO ICTRP]	90
13	((multinational or multi-national or intercontinental or inter-continental or pan-europ$) adj8 (trial or trials or protocol$ or multicent$ or multi-cent$ or multisite or multi-site)).mp.	17
14	((trial or trials or protocol$) and (((multicent$ or multi-cent$ or multisite or multi-site) and sites) or centres or centers) and countries).mp.	26
15	((UK or United Kingdom) and (EU or Europ$) and (trial$ or protocol$)).mp.	113
16	(europe/ or albania/ or alps/ or andorra/ or austria/ or balkans/ or baltic countries/ or belarus/ or belgium/ or bosnia herzegovina/ or bulgaria/ or caucasus/ or central europe/ or croatia/ or cyprus/ or czechoslovakia/ or danube river/ or eastern europe/ or france/ or fyr macedonia/ or germany/ or gibraltar/ or greece/ or hungary/ or iberian peninsula/ or italy/ or liechtenstein/ or luxembourg/ or malta/ or mediterranean/ or moldova/ or monaco/ or montenegro/ or netherlands/ or papal states/ or poland/ or "republic of ireland"/ or romania/ or russia/ or san marino/ or scandinavia/ or serbia/ or slovenia/ or soviet union/ or switzerland/ or turkey/ or ukraine/ or yugoslavia/) and united kingdom/ and (trial$ or protocol$).mp.	22
17	(exp africa/ or exp asia/ or exp middle east/ or exp americas/ or exp oceania/) and exp europe/ and (trial$ or protocol$).mp.	64
18	or/12-17 [main international trial requirement]	303
19	methodology/	153
20	exp research strategies/	16557
21	challenge$.mp.	14704
22	(((trial$ or design) adj5 efficiency) or efficiencies).mp.	270
23	((complexit$ or guidance or guidelines or recommendations or considerations or issues or obstacles or barriers) and trial$).ti.	87
24	((lesson or lessons or pitfall or pitfalls) and (design$ or plan$)).mp.	1351
25	(trial$ adj4 (manag$ or run or running or conducting)).mp.	208
26	(design$ or rationale$ or implement$ or methodol$).ti.	7553
27	management/ or business management/ or corporate management/ or development management/ or joint management/ or office management/ or operational management/ or process management/ or programme management/ or project	9141

For peer review only

	management/ or quality management/ or research management/ or strategic management/ or team management/ or work organisation/ or administration/ or leadership/ or management communication/ or management operations/ or management planning/ or management practice/ or management process/ or management techniques/	
28	contract management/ or facilities management/ or financial management/ or human resources management/ or information management/ or knowledge management/ or materials & supplies management/ or physical distribution management/ or resource management/ or risk management/	10733
29	(((good or best) adj3 practice$) and trial$).ti.	13
30	(trial$ adj2 (authoris$ or authoriz$)).mp.	1
31	or/19-30	55720
32	((trial$ adj5 insur$) or insuring or indemni$).mp.	171
33	((add$ or remov$) adj5 (arm or arms)).mp.	7
34	gdpr.mp.	16
35	exp ethics/ or ethic$.ti.	4861
36	ethics committees/	135
37	((data adj8 secur$) or (data adj3 (share$ or sharing)) or data management or data governance).mp.	839
38	(trial$ adj3 infrastructure).mp.	1
39	statistical model/ or statistical analysis/ or statistical design/ or (statistical adj1 design$).mp.	551
40	software.mp.	1768
41	reporting.ti. or (reporting adj4 (quality or guideline$)).mp.	1107
42	(recruit$ adj4 (effective$ or model$ or pause$ or pausing)).mp. or recruit$.ti.	1058
43	(decision$ adj2 algorithm$).mp.	10
44	sponsor$.ti.	190
45	fund$.ti.	5155
46	exp financing/ or (financ$ not incentiv$).ti.	16452
47	resourcing.mp.	358
48	economic evaluation/ or economic.ti.	3423
49	(trial$ adj1 cost$).mp. or (costs or costing).ti.	3347
50	contract research org$.mp.	4
51	(regulations or (regulat$ adj3 framework$)).mp. or drug regulations/ or regulations/	6548
52	((legal$ or law or laws or legislat$ or policy or policies or rule or rules) adj5 (UK or United Kingdom or brit$ or EU or Europe$ or framework$ or national or government$)).mp.	14895
53	(clinical trial regulation or "536/2014").mp.	3
54	((EU or Europe or European) adj4 representative$).mp.	25
55	collaborat$.ti.	1607
56	(harmonis$ or harmoniz$).mp.	377
57	brexit.mp.	193
58	(safety adj3 (monitor$ or governance)).mp. or drug safety/	191
59	(((intervention or IMP or investigational medicin$ or drug$) adj6 (supply$ or inventory or export$ or import$)) or procur$).mp.	2378
60	(treatment selection or site selection).mp.	27
61	workload$.mp.	3445
62	exp contracts/ or (contract or contracts).mp.	7573
63	((fund$ or collaborat$ or delegat$ or site or sites or research or sponsor$) adj5 (agreement or agreements)).mp.	112
64	((operational or practical or legal or administrative or financial or procedural or ethical or methodolog$ or statistical or recruitment or sponsor$ or logistic$ or design) adj4 (complexit$ or guidance or guidelines or recommendations or considerations or issues or challenges or obstacles or barriers or difficulties or advantages or disadvantages)).mp.	5882
65	or/32-64	71491
66	(8 or 18) and (31 or 65)	186
67	limit 66 to yr="2006 -Current"	110
68	limit 67 to english	110
69	68 not systematic review.ti.	92

For peer review only

2 DATA EXTRACTION TEMPLATE

Data was extracted on the elements listed in Table S1 for all identified studies.

Table S1. Data extracted from included studies

- First author
- Publication year
- Study type
- Trial acronym
- Trial phase
- Blinding status
- Trial status
- Start date
- End date
- Number and location of sites
- Total sample size
- What condition was the study on?
- Type of intervention
- Number of arms and interventions in each arm
- What is the primary outcome?

Sponsorship
- Type of sponsor
- Was it the same sponsor across all sites?
- Details of additional sponsorship

Funding
- Main funding source
- Details of what was funded
- Did they fund all sites?
- Any funding challenges or solutions?
- Insurance details
- Was there a standard operating procedure?

Regulatory
- Were any guidelines used in oversight, if so what were they?
- Was there an EU representative?
- Details of GDPR
- Details of trial monitoring and oversight
- Details of auditing
- Were site specific documents prepared?

Trial management
- Name of randomisation system
- Process of adding and removing arms
- Process of adding and removing sites
- How were interventions chosen?
- Was there a trial steering committee present?
- Recruitment challenges and solutions
- Details of protocol amendments
- Staff training
- Name of data management system
- Any reported data management issues?

Intervention
- Who supplied the intervention?
- Was it the same suppler across all sites?
- Details of procurement and distribution
- Were pharmacies/pharmacists involved?
- How were adverse events monitored?
- Details of intervention licensing

Biospecimens
- What specimens were collected?
- Were they collected at all sites?
- Where were specimens processed?
- Details of specimen transport between sites

Agreements
- Was a data sharing agreement present?
- Who was the agreement between?
- Were site agreements present?
- Details of agreement challenges and solutions
- Who sought ethics approval?
- Details of ethics approval
- What was the contractual responsibility of site leads and stakeholders?
- Were materials translated?
- Details of translation and communication between sites

For peer review only

3 SUMMARY OF INCLUDED STUDIES

Table S2. Summary of included studies

First author (year)	Trial name	Trial design	Population	Number of sites	Site locations	Main coordinating centre(s)	Total participants	Intervention	Primary outcome	Enrolment period
Aban (2008)[25]	MGTX	Parallel	Myasthenia gravis	79	Global	USA	126	Other	Effectiveness	June 2006 – December 2015
Aitken (2008)[32]	PROMOTION	Parallel	Coronary artery disease	5	North America, Oceania	USA	3522	Behavioural	Prevention	February 2001 – June 2006
Angus (2020)[33]	REMAP-CAP	Adaptive-platform	Severe pneumonia and COVID-19		UK/EU, North America, Oceania, Asia	Australia, Thailand		Drug	Effectiveness	April 2016 – present
Aryal (2021)[38]	REMAP-CAP	Adaptive-platform	Severe pneumonia and COVID-19		UK/EU, North America, Oceania, Asia	RCC in Australia and Thailand		Drug	Effectiveness	April 2016 – present
Antic (2015)[42]	SAVE	Parallel	Obstructive sleep apnoea	89	Oceania, North America, South America, Asia, UK/EU	RCC in Australia, Brazil, China, India and Spain	2717	Device	Prevention	September 2008 – December 2015
Babiker (2013)[18]	START	Parallel	HIV	237	North America, South America, UK/EU, Oceania, Africa	RCC in Denmark, UK, Australia, USA	4000	Drug	Efficacy	April 2009 – July 2022
Berthon-Jones (2015)[26]	ALTAIR	Parallel	HIV	36	Asia, Oceania, UK/EU, North America, South America		322	Drug	Effectiveness	February 2007 – November 2011
Bryant (2021)[49]	TBTC Study 31	Parallel	Tuberculosis	34	North America, South America, Asia, Africa	USA	2516	Drug	Effectiveness	January 2016 – May 2021
Carli (2013)[43]	SEYLE	Cluster	Suicide	11	UK/EU	Sweden	11110	Behavioural	Prevention	September 2009 – January 2012
Clasen (2020)[53]	HAPIN	Parallel	Low birth weight		Asia, North America, South America, Africa	USA		Device	Prevention	September 2017 – present
Coomarasamy (2016)[50]	PROMISE	Parallel	Recurrent miscarriage	45	UK/EU	UK	836	Drug	Efficacy	June 2008 – May 2012
Crow (2018)[19]	FOR-DMD	Parallel	Duchenne muscular dystrophy	40	North America, UK/EU	USA	196	Drug	Effectiveness	January 2013 – November 2019
del Álamo (2022)[20]										
Denholm (2022)[51]	ASCOT ADAPT	Adaptive-platform	COVID-19		Oceania, Asia			Drug	Effectiveness	February 2021 – present
Dutton (2009)[44]	CONTROL	Parallel	Trauma	75	North America, South America, UK/EU, Asia, Africa	USA	576	Drug	Efficacy	October 2005 – September 2008
Eikelboom (2022)[39]	ACT	Factorial	COVID-19	62	North America, South America, Africa, Asia		6528	Drug	Effectiveness	April 2020 – February 2022
Fogelholm (2017)[45]	PREVIEW	Factorial	Pre-diabetes	8	UK/EU, Oceania		2326	Behavioural	Prevention	June 2013 – December 2018
Franciscus (2014)[52]	TRIGR	Parallel	Type 1 diabetes	77	North America, Oceania, UK/EU	USA	5156	Other	Prevention	May 2002 – December 2006
Fulda (2023)[48]	REPRIEVE	Parallel	HIV		North America, South America, Africa, Asia, UK/EU	USA		Drug	Prevention	March 2015 – present
Goossens (2021)[27]	REMAP-CAP	Adaptive-platform	Severe pneumonia and COVID-19		UK/EU, North America, Oceania, Asia	Australia, Thailand		Drug	Effectiveness	April 2016 – present

For peer review only

Grarup (2015)[31]	START	Parallel	HIV	237	North America, South America, UK/EU, Oceania, Africa	RCC in Denmark, UK, Australia, USA	4000	Drug	Efficacy	April 2009 – July 2022
Hata (2020)[40]	PATHWAY	Parallel	Breast cancer	23	Asia		185	Drug	Efficacy	February 2018 – July 2022
Herrick (2012)[34]	FDTT	Parallel	Functional dyspepsia	8	North America		292	Drug	Efficacy	October 2006 – July 2013
Jeon (2016)[46]	CLEAR III	Parallel	Intracerebral haemorrhage	73	North America, South America, UK/EU, Asia	USA	500	Drug	Efficacy	September 2009 – January 2015
Kenyon (2011)[21]	STICH II	Parallel	Intracerebral haemorrhage	126	North America, Oceania, UK/EU, Asia, Africa	UK	601	Other	Efficacy	January 2007 – August 2015
Kesho Bora Study Group (2011)[35]	Kesho-Bora	Parallel	HIV	5	Africa	Switzerland	824	Drug	Efficacy	June 2005 – August 2008
Kolitsopoulos (2013)[47]	ZODIAC	Parallel	Schizophrenia	226	North America, South America, UK/EU, Asia		18240	Drug	Effectiveness	February 2002 – April 2007
Larson (2016)[22]	INSIGHT trials					RCC in UK, Denmark, USA and Australia				
Lingor (2021)[23]	ROCK-ALS / ROCK-ALS-US	Parallel	Amyotrophic lateral sclerosis		North America, UK/EU			Drug	Efficacy	February 2019 – present
Minisman (2012)[5]	MGTX	Parallel	Myasthenia gravis	79	Global	USA	126	Other	Effectiveness	June 2006 – December 2015
Murray (2022)[24]	TICO	Adaptive-platform	COVID-19		North America, UK/EU, Asia, Africa	USA with 8 RCC		Drug	Effectiveness	August 2020 – present
Neaton (2010)[9]	INSIGHT trials					RCC in UK, Denmark, USA and Australia				
Ravinetto (2013)[36]	4ABC	Parallel	Malaria	12	Africa	Belgium	4112	Drug	Efficacy	July 2007 – December 2009
Reams (2018)[41]	DOVE	Parallel	Sickle cell disease	51	North America, South America, UK/EU, Asia, Africa		341	Drug	Efficacy	April 2013 – December 2015
Seal (2006)[28]		Factorial	Endophthalmitis	24	UK/EU, Asia	UK	35000	Drug	Efficacy	September 2003 – May 2006
Spencer (2012)[54]	AWARD-5	Adaptive	Type 2 diabetes	111	North America, UK/EU, Asia		1202	Drug	Safety	August 2008 – July 2012
Sydes (2011)[29]	STAMPEDE	Adaptive-platform	Prostate cancer		UK/EU	UK		Drug	Efficacy	July 2005 – present
Zimmer (2010)[37]	BAMSG 3-01	Parallel	Cryptococcal meningitis	13	North America, Asia	USA	143	Drug	Efficacy	May 2005 – April 2008

RCC: Regional Coordinating Centre; RCT: Randomised Controlled Trial.

For peer review only

4 OPERATIONAL COMPLEXITIES REPORTED BY PUBLICATION

Table S3. Operational complexities in conducting international trials

Major barriers	Source
**Study set-up**	
Sponsorship, insurance and need for EU legal representative	Aban (2008); Babiker (2013); Berthon-Jones (2015); Crow (2018); del Álamo (2022); Goossens (2021); Kenyon (2011); Lingor (2021); Minisman (2012); Murray (2022); Neaton (2010); Seal (2006); Sydes (2011)
Funding	Aban (2008); Aitken (2008); Angus (2020); Babiker (2013); Crow (2018); del Álamo (2022); Goossens (2021); Herrick (2012); Kenyon (2011); Kesho Bora Study Group (2011); Larson (2016); Lingor (2021); Ravinetto (2013); Zimmer (2010)
Lack of harmonisation in ethics and regulatory approvals	Aban (2008); Aitken (2008); Aryal (2021); Babiker (2013); Berthon-Jones (2015); del Álamo (2022); Eikelboom (2022); Goossens (2021); Grarup (2015); Hata (2020); Kenyon (2011); Minisman (2012); Murray (2022); Neaton (2010); Ravinetto (2013); Reams (2018); Zimmer (2010)
**Site set-up**	
Training	Aitken (2008); Antic (2015); Aryal (2021); Carli (2013); Crow (2018); Dutton (2009); Fogelholm (2017); Jeon (2016); Kesho Bora Study Group (2011); Kolitsopoulos (2013); Minisman (2012)
Contracts	Babiker (2013); Crow (2018); del Álamo (2022); Goossens (2021); Grarup (2015); Larson (2016); Lingor (2021); Minisman (2012)
Site monitoring	Angus (2020); Aryal (2021); Bryant (2021); Carli (2013); Coomarasamy (2016); Denholm (2022); Dutton (2009); Franciscus (2014); Fulda (2023); Larson (2016); Ravinetto (2013); Seal (2006); Sydes (2011); Zimmer (2010)
Communication	Aitken (2008); Angus (2020); Antic (2015); Crow (2018); Denholm (2022); Fulda (2023); Herrick (2012); Minisman (2012)
Translation of materials	Aban (2008); Babiker (2013); Berthon-Jones (2015); Carli (2013); Crow (2018); Franciscus (2014); Lingor (2021); Zimmer (2010)
**Trial management**	
Trial oversight	Antic (2015); Babiker (2013); Coomarasamy (2016); Dutton (2009); Franciscus (2014); Herrick (2012); Larson (2016); Murray (2022); Spencer (2012)
Site and intervention selection	Angus (2020); Antic (2015); Coomarasamy (2016); Denholm (2022); Dutton (2009); Eikelboom (2022); Fulda (2023); Herrick (2012); Kesho Bora Study Group (2011); Kolitsopoulos (2013); Minisman (2012); Murray (2022); Reams (2018); Zimmer (2010)
Recruitment	Aitken (2008); Antic (2015); Berthon-Jones (2015); Eikelboom (2022); Franciscus (2014); Herrick (2012); Kesho Bora Study Group (2011); Kolitsopoulos (2013); Reams (2018)
**Data management**	Aitken (2008); Aryal (2021); Berthon-Jones (2015); Carli (2013); Coomarasamy (2016); Ravinetto (2013); Seal (2006)
**Intervention management**	
Drug procurement and distribution	Babiker (2013); Bryant (2021); Coomarasamy (2016); Crow (2018); del Álamo (2022); Goossens (2021); Grarup (2015); Hata (2020); Herrick (2012); Jeon (2016); Lingor (2021); Minisman (2012); Murray (2022); Ravinetto (2013); Reams (2018); Seal (2006); Spencer (2012); Zimmer (2010)
Pharmacy involvement	Aban (2008); Bryant (2021); Coomarasamy (2016); Herrick (2012); Jeon (2016); Kolitsopoulos (2013); Minisman (2012)
Monitoring safety and adverse events	Antic (2015); Bryant (2021); Coomarasamy (2016); Hata (2020); Herrick (2012); Minisman (2012); Murray (2022); Zimmer (2010)
Biospecimen processing and transport	Berthon-Jones (2015); Bryant (2021); Denholm (2022); Fogelholm (2017); Franciscus (2014); Fulda (2023); Hata (2020); Lingor (2021)
**Adaptive-specific**	Angus (2020); Aryal (2021); Denholm (2022); Murray (2022); Spencer (2012)

For peer review only

5 PRISMA CHECKLIST

Section and Topic	Item #	Checklist item	Location where item is reported
**TITLE**			
Title	1	Identify the report as a systematic review.	Page 1
**ABSTRACT**			
Abstract	2	See the PRISMA 2020 for Abstracts checklist.	Page 2
**INTRODUCTION**			
Rationale	3	Describe the rationale for the review in the context of existing knowledge.	Page 4
Objectives	4	Provide an explicit statement of the objective(s) or question(s) the review addresses.	Page 4
**METHODS**			
Eligibility criteria	5	Specify the inclusion and exclusion criteria for the review and how studies were grouped for the syntheses.	Page 5
Information sources	6	Specify all databases, registers, websites, organisations, reference lists and other sources searched or consulted to identify studies. Specify the date when each source was last searched or consulted.	Page 5
Search strategy	7	Present the full search strategies for all databases, registers and websites, including any filters and limits used.	Page 5
Selection process	8	Specify the methods used to decide whether a study met the inclusion criteria of the review, including how many reviewers screened each record and each report retrieved, whether they worked independently, and if applicable, details of automation tools used in the process.	Page 5, Figure 1
Data collection process	9	Specify the methods used to collect data from reports, including how many reviewers collected data from each report, whether they worked independently, any processes for obtaining or confirming data from study investigators, and if applicable, details of automation tools used in the process.	Page 5-6
Data items	10a	List and define all outcomes for which data were sought. Specify whether all results that were compatible with each outcome domain in each study were sought (e.g. for all measures, time points, analyses), and if not, the methods used to decide which results to collect.	Page 6
	10b	List and define all other variables for which data were sought (e.g. participant and intervention characteristics, funding sources). Describe any assumptions made about any missing or unclear information.	Page 6
Study risk of bias assessment	11	Specify the methods used to assess risk of bias in the included studies, including details of the tool(s) used, how many reviewers assessed each study and whether they worked independently, and if applicable, details of automation tools used in the process.	NA
Effect measures	12	Specify for each outcome the effect measure(s) (e.g. risk ratio, mean difference) used in the synthesis or presentation of results.	Page 5-6
Synthesis methods	13a	Describe the processes used to decide which studies were eligible for each synthesis (e.g. tabulating the study intervention characteristics and comparing against the planned groups for each synthesis (item #5)).	Page 5-6
	13b	Describe any methods required to prepare the data for presentation or synthesis, such as handling of missing summary statistics, or data conversions.	NA
	13c	Describe any methods used to tabulate or visually display results of individual studies and syntheses.	NA
	13d	Describe any methods used to synthesize results and provide a rationale for the choice(s). If meta-analysis was performed, describe the model(s), method(s) to identify the presence and extent of statistical heterogeneity, and software package(s) used.	Page 5-6
	13e	Describe any methods used to explore possible causes of heterogeneity among study results (e.g. subgroup analysis, meta-regression).	NA
	13f	Describe any sensitivity analyses conducted to assess robustness of the synthesized results.	NA
Reporting bias assessment	14	Describe any methods used to assess risk of bias due to missing results in a synthesis (arising from reporting biases).	NA
Certainty assessment	15	Describe any methods used to assess certainty (or confidence) in the body of evidence for an outcome.	NA
**RESULTS**			
Study selection	16a	Describe the results of the search and selection process, from the number of records identified in the search to the number of studies included in the review, ideally using a flow diagram.	Page 6-7
	16b	Cite studies that might appear to meet the inclusion criteria, but which were excluded, and explain why they were excluded.	Page 6-7 / Figure 1
Study characteristics	17	Cite each included study and present its characteristics.	Page 6-7 / Table 1
Risk of bias in studies	18	Present assessments of risk of bias for each included study.	NA
Results of individual studies	19	For all outcomes, present, for each study: (a) summary statistics for each group (where appropriate) and (b) an effect estimate and its precision (e.g. confidence/credible interval), ideally using structured tables or plots.	Table 2, Page 6-16

For peer review only

Section and Topic	Item #	Checklist item	Location where item is reported
Results of syntheses	20a	For each synthesis, briefly summarise the characteristics and risk of bias among contributing studies.	NA
	20b	Present results of all statistical syntheses conducted. If meta-analysis was done, present for each the summary estimate and its precision (e.g. confidence/credible interval) and measures of statistical heterogeneity. If comparing groups, describe the direction of the effect.	NA
	20c	Present results of all investigations of possible causes of heterogeneity among study results.	NA
	20d	Present results of all sensitivity analyses conducted to assess the robustness of the synthesized results.	NA
Reporting biases	21	Present assessments of risk of bias due to missing results (arising from reporting biases) for each synthesis assessed.	NA
Certainty of evidence	22	Present assessments of certainty (or confidence) in the body of evidence for each outcome assessed.	NA
**DISCUSSION**			
Discussion	23a	Provide a general interpretation of the results in the context of other evidence.	Page 13-15
	23b	Discuss any limitations of the evidence included in the review.	Page 15
	23c	Discuss any limitations of the review processes used.	Page 15
	23d	Discuss implications of the results for practice, policy, and future research.	Page 13-15
**OTHER INFORMATION**			
Registration and protocol	24a	Provide registration information for the review, including register name and registration number, or state that the review was not registered.	Page 5
	24b	Indicate where the review protocol can be accessed, or state that a protocol was not prepared.	NA
	24c	Describe and explain any amendments to information provided at registration or in the protocol.	NA
Support	25	Describe sources of financial or non-financial support for the review, and the role of the funders or sponsors in the review.	Page 3
Competing interests	26	Declare any competing interests of review authors.	Page 3
Availability of data, code and other materials	27	Report which of the following are publicly available and where they can be found: template data collection forms; data extracted from included studies; data used for all analyses; analytic code; any other materials used in the review.	

For peer review only

6 PROTOCOL

Operational best practice in international clinical trials: a systematic review protocol

Support and registration:
This systematic review has been funded by the NIHR (Application Accelerator Award reference number: NIHR153955). The funder will have no further role in any aspect of the review. This protocol has been registered with the Open Science Framework (registration DOI: https://doi.org/10.17605/OSF.IO/YVTJB) (1).

Aim:
The aim of this systematic review is to identify models of best practice and lessons learned from the set up and delivery of international clinical trials, specifically in regard to management of cross-border regulatory and logistical requirements.

Rationale:
Research practices have transformed over the last decade, evolving from large single centred trials to more global trials that promote international collaboration. International trials offer numerous advantages over single-centre including reduced operational costs particularly in developing countries, faster recruitment and expand the horizon for the availability of treatments. Despite their benefits, the process of expanding to multiple sites raises logistical challenges due to the diversity in laws, ethics, guidelines and regulations between countries.

Search strategy:
The search strategy will be designed in collaboration with an experienced information specialist and will be peer-reviewed by another information specialist using the PRESS checklist. Thesaurus headings and keywords have been used as appropriate, and the search will be translated to other sources accordingly. The search strategy will cover four areas: "international trials", "adaptive design trials", "study design", and "specific challenges" and requirements of designing and running trials. Multiple approaches to combining these aspects will be used to achieve a practical quantity of specific, relevant results, while also mitigating risks of missing relevant material. A wide range of subject headings and search terms in appropriate fields will be used for all concepts in the search, including proximity searching to cover the many variations on phrases applicable to these topics. We will also apply a date filter to include studies published after 2005 as international trials have become prominent only in the last 15-20 years. We will search Medline, Embase and Health Management Information Consortium (HMIC) for appropriate studies.

Participants or population:
As the focus is on the implementation and infrastructure of the trial, any clinical population and any condition will be considered of interest.

Intervention:
Clinical trials on any drug, device or therapeutic intervention will be considered if they are multi-centre and trial site locations are in more than one country.

Comparator:
Trials will be eligible regardless of comparator.

Study designs to be included:
Any multi-centre randomised trial design with sites in at least two different countries will be included.

Eligibility criteria:
The following types of studies will be included:

1. Any study reporting operational challenges to conducting international trials (involving two or more countries)
2. Available in English
3. Published after 2005

Conference abstracts, systematic reviews and protocols will be excluded.

Main outcome:
Any outcomes related to the best practice models and lessons learned in aspects of set up and delivery of international trials.

We will specifically look at:

- Sponsorship and funding: balancing UK, EU and other international trial regulations, policies and procedures in sponsorship of the overall trial
- Trial management structure: legal, ethical and regulatory matters, safety and monitoring standards, processes for adding and/or removing trial arms, GDPR and clinical data management system requirements
- Intervention management: pharmacy involvement, procurement, distribution, delivery of interventions

For peer review only - http://bmjopen.bmj.com/site/about/guidelines.xhtml

- Contracting: agreements of collaboration, sites, delegation, sample transfer logistics arrangements and data sharing.

Data management:
Zotero will be used to manage the studies throughout the review. Rayyan will be used to screen records.

Strategy for data synthesis:
Two reviewers will independently screen the titles and abstracts of the studies retrieved by the search. For studies deemed eligible, or studies where it is impossible to decide eligibility from the abstract, the full text will be retrieved, and two reviewers will independently assess for inclusion. Any disagreements will be resolved through discussion or if necessary, by reference to a third reviewer.

Data extraction will be undertaken by one reviewer and checked by a second, with discrepancies resolved by consultation with a third. Where studies are reported in multiple publications, we will extract relevant data from all publications but consider as one study. Where data is missing or unclear, we will contact authors to request details or clarification. The following data will be extracted from included studies:

- Citation information
- Study design variables: sample size, phase
- Study population and objective variables: age, condition, experimental interventions, primary outcome
- Regulatory information: status, location, availability of results, start and completion date
- Outcome data: Sponsorship information; funding information; trial management process information; intervention/pharmacy process information; detail on the contracting processes

In the first instance, we will present a summary of study characteristics and outcome data in a series of structured tables to give a clear picture of the available evidence.

Language:
Only studies published in English will be included.

References

1. Gumber L, Pratt A, Bardgett M, Inskip A, Still M, Phillipson J, et al. Operational best practice in international clinical trials: a systematic review protocol 2023. osf.io/yvtjb.

For peer review only
